# Classification of Calcium-Dependent Protein Kinases and Their Transcriptional Response to Abiotic Stresses in Halophyte *Nitraria sibirica*

**DOI:** 10.3390/plants14193091

**Published:** 2025-10-07

**Authors:** Lu Lu, Ting Chen, Tiangui Yang, Chunxia Han, Jingbo Zhang, Jinhui Chen, Tielong Cheng

**Affiliations:** 1 State Key Laboratory of Tree Genetics and Breeding, Co-Innovation Center for Sustainable Forestry in Southern China, Nanjing Forestry University, Nanjing 210037, China; lulu2020@njfu.edu.cn (L.L.); chenting20001209@126.com (T.C.); YL1181399tiangui@163.com (T.Y.); chenjh@njfu.edu.cn (J.C.); 2 Key Laboratory of Forest Genetics and Biotechnology of Ministry of Education, Nanjing Forestry University, Nanjing 210037, China; 3 Experimental Center of Desert Forestry, Chinese Academy of Forestry, Dengkou 015200, China; hanchunxia0116@126.com (C.H.); nmzhangjb@126.com (J.Z.); 4 College of Ecology and Environment, Nanjing Forestry University, Nanjing 210037, China

**Keywords:** calcium-dependent protein kinase, halophyte, *Nitraria sibirica*, abiotic stress, salinity

## Abstract

Calcium-dependent protein kinases (CDPKs) are key Ca^2+^ sensors in plants, mediating responses to abiotic stresses via phosphorylation signaling. In the halophyte *Nitraria sibirica*, which thrives in saline soils, we identified 19 *CDPK* genes (*NsCDPK*s) and classified them into four canonical angiosperm clades, highlighting conserved functional modules. Promoter analysis revealed diverse *cis*-acting elements responsive to light, hormones (ABA, MeJA, auxin, GA, SA), and abiotic stresses (drought, cold, wounding), along with numerous MYB binding sites, suggesting complex transcriptional regulation. Transcriptome profiling under salt stress (100 and 400 mM NaCl) showed induction of most *NsCDPK*s, with several genes significantly upregulated in roots and stems, indicating coordinated whole-plant activation. These salt-responsive *NsCDPK*s were also upregulated by cold but repressed under PEG-simulated drought, indicating stress-specific regulatory patterns. Fifteen MYB transcription factors, differentially expressed under salt stress, were predicted to interact with *NsCDPK* promoters, implicating them as upstream regulators. This study identified a potential salt- and cold-responsive *CDPK* regulatory module and a MYB-mediated transcriptional hierarchy in *N. sibirica*, providing insights into the molecular mechanisms of salinity adaptation and highlighting candidate genes that could be explored for improving salt tolerance in crop species.

## 1. Introduction

To survive the continual biotic and abiotic stresses present in their environments, plants have evolved highly sophisticated defense mechanisms, with diverse signal transduction pathways playing a central role. Among these pathways, calcium ions (Ca^2+^) are recognized as ubiquitous and pivotal second messengers, mediating the perception and transduction of a wide spectrum of environmental and developmental signals [1,2,3,4]. The remarkable complexity and versatility of Ca^2+^ signaling position it as a central hub in the regulation of plant growth, development, and resilience to both abiotic and biotic stresses [5,6]. A critical question in plant biology is how specificity of response is achieved during Ca^2+^-mediated signal transduction. Accumulating evidences indicate that distinct stimuli elicit unique calcium signatures, characterized by variations in the kinetics, amplitude, and subcellular origin of Ca^2+^ influx [7]. Unlike most other ions, Ca^2+^ does not freely diffuse within the cytoplasm, necessitating tightly regulated transport and compartmentalization [8]. Dynamic changes in cytosolic Ca^2+^ concentration encode precise information, which is subsequently decoded by a diverse array of Ca^2+^-binding proteins, such as C2 domain containing proteins [9,10]. These proteins modulate downstream signaling cascades, ultimately orchestrating cellular responses that are critical for plant survival and adaptation [11,12].

In plants, Ca^2+^ sensors mainly include calmodulins (CaMs), CaM-like proteins (CMLs), calcineurin B-like proteins (CBLs) and calcium-dependent protein kinases (CDPKs, also known as CPKs) [13,14,15,16,17,18]. CDPKs represent a unique family of serine/threonine kinases that directly couple Ca^2+^ binding to protein phosphorylation, thereby transducing Ca^2+^ signals into specific cellular outcomes [4,7]. CDPKs possess both a kinase domain and a calmodulin-like regulatory domain within a single polypeptide, enabling rapid and efficient signal transduction, which are characterized by a modular structure comprising four principal domains: a N-terminal variable region, a serine/threonine kinase domain, an autoinhibitory junction region, and a calmodulin-like domain containing four EF-hand Ca^2+^-binding motifs [4,19]. The N-terminal domain often harbors N-myristylation and/or S-palmitoylation sites, which influence subcellular localization and protein–protein interactions [20,21]. The kinase domain catalyzes phosphorylation of target substrates, while the autoinhibitory region regulates kinase activity in a Ca^2+^-dependent manner [22]. The EF-hand motifs are responsible for Ca^2+^ sensing, and their occupancy induces conformational changes that relieve autoinhibition and activate the kinase [23].

Despite its conservation, the number of *CDPK* genes varies significantly among species, primarily due to lineage-specific evolutionary events such as gene expansion, tandem and segmental duplications, and gene loss [7,24,25]. In model dicot plant *Arabidopsis thaliana* (*A. thaliana*) and *Populus trichocarpa* (*P. trichocarpa*), 34 and 30 *CDPK* genes have been identified, respectively [7,25]. In monocot species such as rice (*Oryza sativa*, *O. sativa*) harbors 31 *CDPK* genes [26], while wheat (*Triticum aestivum*, *T. aestivum*) only possesses 20 *CDPK*s [27]. In economically important crops, cotton (*Gossypium hirsutum*, *G. hirsutum*) contains 41 *CDPK*s [28], while cucumber (*Cucumis sativus*, *C. sativus*) and tomato (*Solanum lycopersicum*, *S. lycopersicum*) contain 19 and 29 *CDPK*s, respectively [29,30].

*CDPK*s have been implicated in a wide array of physiological processes, including growth and development, hormone signaling, pathogen defense, and abiotic stress tolerance [31,32,33,34,35]. The involvement of *CDPK*s in abiotic stress signaling has been extensively documented. *CDPK*s regulate the synthesis of osmoprotectants and antioxidants, thereby enhancing cellular tolerance to osmotic and oxidative stress [7,36]. Recent studies in *A. thaliana* have elucidated a critical role for CDPK16 protein in modulating plant responses to hypoxic stress. CDPK16 mediates hypoxia tolerance by phosphorylating the plasma membrane-associated NADPH oxidase, respiratory burst oxidase homolog D (RBOHD), thereby regulating the production of reactive oxygen species (ROS) [37]. Physiological analyses demonstrated that overexpression of cotton *CDPK16* in *Arabidopsis* significantly enhances drought stress tolerance. Transgenic plants exhibited improved osmotic adjustment, as evidenced by increased accumulation of osmoprotectants, alongside elevated activities of key antioxidant enzymes [38]. Rice calcium-dependent protein kinase isoform *OsCDPK10* has been identified as a positive regulator of stress tolerance, functioning primarily through enhancement of the plant’s antioxidant capacity [39]. In rice, overexpression of *OsCDPK12* confers improved salt tolerance by activating the antioxidant system and reducing Na^+^ accumulation [40]. Ectopic expression of cucumber *CDPK6* significantly improved survival rates and reduced stomatal apertures of transgenic plants under salt stress conditions [41]. Under salt stress, CDPK proteins modulate ion transporters, such as the plasma membrane H^+^-ATPase and the Na^+^/H^+^ antiporter, to maintain ionic homeostasis [19,42].

The transcriptional regulation of *CDPK* genes is mediated by complex networks of *cis*-acting elements and trans-acting factors. Promoter regions of *CDPK*s contain diverse *cis*-acting elements responsive to light, phytohormones, and environmental stresses [43,44,45]. These elements facilitate precise spatial and temporal control of *CDPK* expression in response to endogenous and exogenous cues. Abscisic acid (ABA)-responsive elements are particularly prominent in *CDPK* promoters, reflecting the central role of ABA in abiotic stress signaling [46]. The interplay between ABA and Ca^2+^ signaling pathways enables plants to fine-tune their responses to osmotic and ionic stress [7,42]. MYBs are involved in the regulation of secondary metabolism, cell wall biosynthesis, and stress responses, which represent a major class of potential upstream regulators that bind to specific motifs in CDPK promoters and modulate their expression under stress conditions [47,48].

Halophytes are plants that thrive in saline environments, exhibiting remarkable physiological and molecular adaptations to high salt, drought, and temperature extremes [49,50]. These adaptations include enhanced ion compartmentalization, osmoprotectant accumulation, antioxidant defenses, and robust stress signaling networks. *Nitraria sibirica* (*N. sibirica*) is a perennial halophyte native to arid and semi-arid regions, renowned for its exceptional tolerance to salinity, drought, and temperature fluctuations [51,52,53]. Its ability to survive and reproduce in extreme environments makes it an ideal model for dissecting the molecular mechanisms underlying stress resilience in desert plants. Despite the ecological and agricultural significance of halophytes, the genomic and functional characterization of key stress signaling components, such as CDPK proteins, remains limited. Previous studies have focused primarily on model glycophytes like *Arabidopsis* and rice, leaving a critical knowledge gap regarding the evolution, structure, and regulatory networks of *CDPK*s in halophytic species [49,50].

To give the central role of *CDPK*s in stress signaling and the unique adaptive strategies of halophytes, this study offers new insights on the full complement of *CDPK* genes in the *N. sibirica* genome. The structural features, conserved motifs, and phylogenetic relationships of NsCDPK proteins were analyzed. To identify the functional characterization, the expression pattern of *NsCDPK*s under salt, cold, and drought stress were examined. To identify and functionally analyze upstream regulators, *cis*-acting elements in *NsCDPK* promoters were profiled to elucidate their potential regulatory roles. Furthermore, the potential upstream transcription factors of salt-responsive *NsCDPK*s were identified. By elucidating the genomic architecture, evolutionary conservation, and regulatory networks of *CDPK*s, this study would offer new insights into the adaptive mechanisms of halophytes and identifies promising genetic targets for engineering stress resilience in crops.

## 2. Results

### 2.1. Identification and Identity Analysis of N. sibirica CDPK Family Members

CDPK proteins from *A. thaliana* (AtCDPKs) were used as query sequences to search against the *N. sibirica* proteome. As a result, 19 *CDPK* genes were identified in the *N. sibirica* genome. The identity of these proteins was further validated through Pfam search and conserved domain analyses. Detailed protein sequence information of the identified NsCDPKs is provided in the Supplementary Data S1. The predicted NsCDPK proteins range in length from 498 to 979 amino acids, with an average length of approximately 582 amino acids. Correspondingly, their molecular weights span from 56.00 to 110.78 kDa. Most NsCDPKs have isoelectric points (pI) below 7.0, except for NISI07G3121 (pI = 9.25), with the average pI value being 6.22 (Table 1).

Analysis of protein stability revealed that 13 NsCDPKs have instability index (II) values below 40, indicating they are relatively stable proteins (proteins with II values below 40 are considered stable) [54]. The aliphatic index (AI), which is positively correlated with protein thermostability, ranges from 74.41 to 89.07 among NsCDPK proteins [55]. All NsCDPKs exhibit negative grand average of hydropathicity (GRAVY) values, with an average of -0.45, indicating that these proteins are generally hydrophilic (negative GRAVY values suggest enhanced protein-water interactions [56]) (Table 1).

Secondary structure prediction showed that alpha helices (36.25–47.74%) and random coils (30.13–42.60%) are the predominant secondary structural elements in all NsCDPKs, followed by extended strands (8.96–15.45%) and beta turns (7.14–9.70%) (Table 2).

### 2.2. Conserved Motifs Analysis and Phylogenetic Study for NsCDPK Proteins

**Figure 1:**
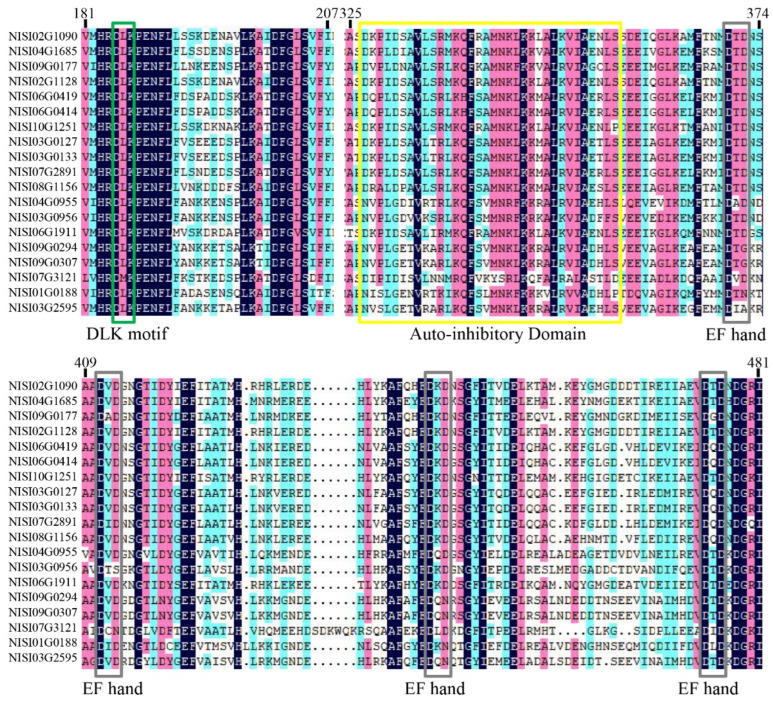
Multiple sequence alignment and conserved domain analysis of *N. sibirica* calcium-dependent protein kinases (NsCDPKs). Sequence alignment and conserved domain analysis of the 19 NsCDPK proteins sequences. Dark blue shading represents identical residues; pink and light blue shading indicates similar residues. The boxes with different colors represent the corresponding conserved domains.

**Figure 2:**
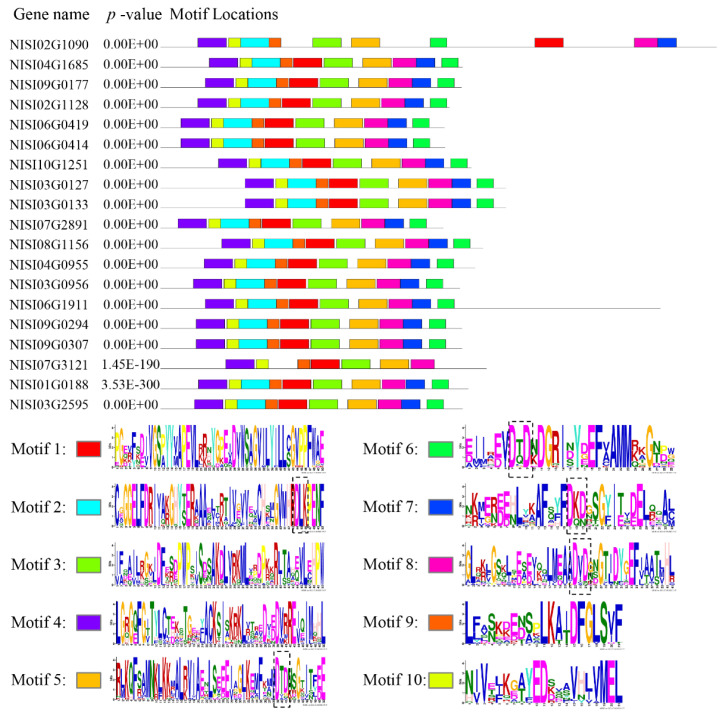
Conserved motif analysis for NsCDPK proteins. Conserved motif distribution of NsCDPK proteins. 10 motifs were set as the cut-off for conserved motif search. 10 boxes with different colors at the right side indicate the 10 motifs following with the corresponding conservative sequences, respectively. Dashed box in Motif 2 shows the DLK motif. Dashed boxes in Motifs 5–8 represent EF hand motif.

To further characterize the structural features of the identified CDPK proteins, conserved domain analyses were performed using DNAMAN and MEME online tools. Multiple sequence alignment with DNAMAN revealed that all NsCDPKs possess a DLK motif, an auto-inhibitory domain, and four Ca^2+^-binding EF-hand motifs (Figure 1). Complementary motif analysis with MEME identified ten conserved motifs within the NsCDPK protein sequences. Notably, all NsCDPKs contain motifs corresponding to the DLK and EF-hand domains, underscoring their structural conservation (Figure 2).

**Figure 3:**
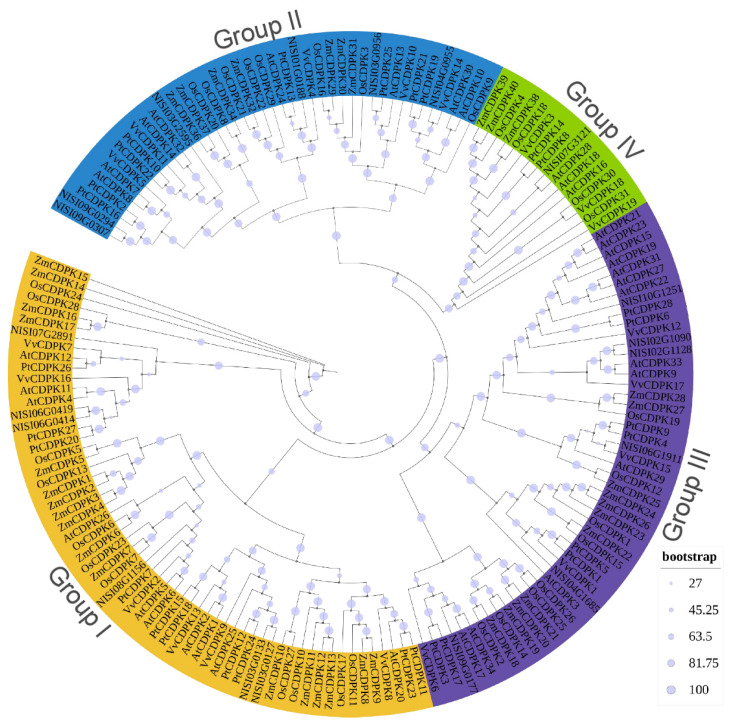
Phylogenetic tree of CDPK proteins. 19 *Nitraria sibirica* CDPK protein (NsCDPK) sequences, 34 *Arabidopsis thaliana* CDPKs (AtCDPKs), 19 *Vitis vinifera* CDPKs (VvCDPKs), 28 *Populus trichocarpa* CDPKs (PtCDPKs), 31 *Oryza sativa* CDPKs (OsCDPKs), and 38 *Zea mays* CDPKs (ZmCDPKs) were used for phylogenetic analysis. NsCDPK protein sequences are listed in the Supplementary Data S1. Phylogenetic tree was constructed in TBtools using “One step build a ML tree” method with default parameters [57]. The tree is divided into four groups (Group I–IV), consistent with the classification of AtCDPK proteins.

To elucidate the phylogenetic relationships of CDPK proteins across different plant species, a Maximum Likelihood (ML) phylogenetic tree was constructed based on 169 CDPK protein sequences, including 19 NsCDPKs of *N. sibirica*, 34 AtCDPKs of *A. thaliana*, 19 CDPKs of *V. vinifera*, 28 CDPKs of *P. trichocarpa*, 31 CDPKs of *O. sativa*, and 38 CDPKs of *Zea mays* (*Z. mays*) (Figure 3). Based on the previous classification of AtCDPK proteins, the 19 NsCDPKs were grouped into four distinct clades, designated as Groups I–IV. Specifically, Group I–III contains 6 NsCDPKs for each, while Group IV contains 1 NsCDPK. These results suggest a high degree of conservation in the distribution of CDPK protein groups among these plant species, indicating potential evolutionary and functional similarities among them.

### 2.3. Chromosomal Location and Synteny Analysis of NsCDPKs

**Figure 4:**
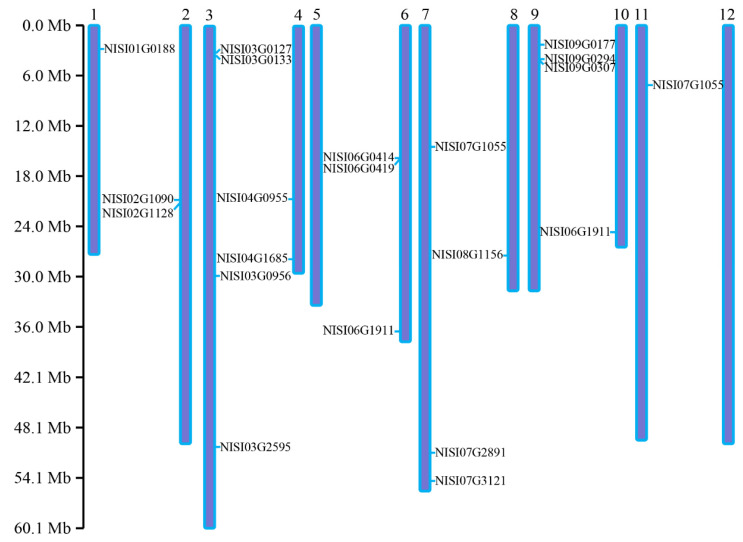
Location of *NsCDPK*s on *N. sibirica* chromosomes. Chromosomal locations of 19 *NsCDPK*s are shown on corresponding chromosomes from top (start) to bottom (end) according to genome annotation. Number 1–12 on the chromosomes indicate.

All *NsCDPK* genes were mapped to the 12 chromosomes of *N. sibirica* to analyze their chromosomal distribution. A total of 19 *NsCDPK*s were located on chromosomes 1–4 and 6–11. Specifically, chromosome 3 harbors the highest number of *NsCDPK*s (four), followed by chromosome 6, 7 and 9 with three *NsCDPK*s, and chromosomes 2 and 4 with two *NsCDPK*s each. While chromosomes 1, 8, 10 and 11 contain a single *NsCDPK* each. No *NsCDPK*s were identified on chromosomes 5 and 12 (Figure 4). These results indicate that *NsCDPK*s are unevenly and apparently randomly distributed across the *N. sibirica* chromosomes.

**Figure 5:**
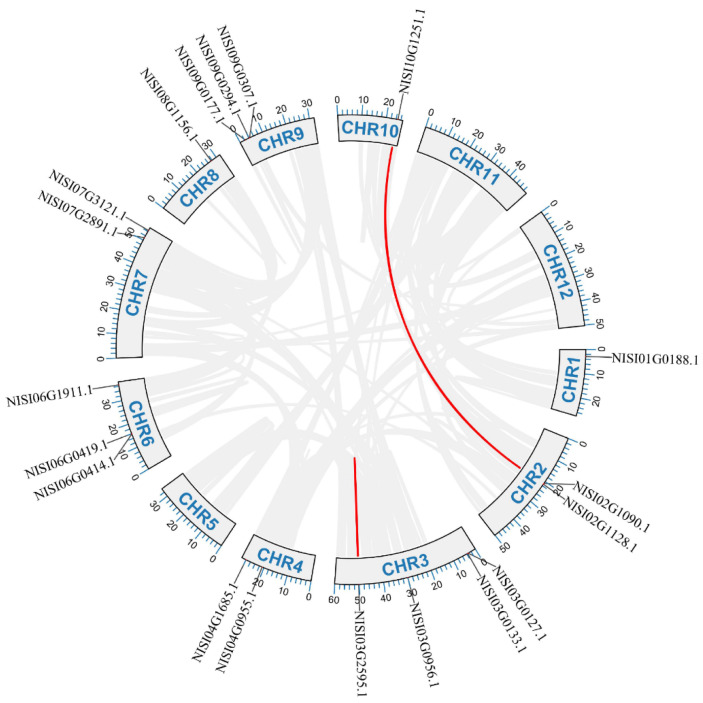
Genome-wide synteny analysis of *CDPK* gene family in *N. sibirica*. Distribution and segmental duplication of *CDPK* genes in *N. sibirica*. The gray panels with blue “CHR1–12” words show the 12 chromosomes of *N. sibirica*. The gray lines among chromosomes indicate the homologous gene pairs in *N. sibirica* genome. Red lines connect homologous *NsCDPK*s. CHR: chromosome.

To investigate gene duplication events among *NsCDPK*s, a comprehensive analysis of chromosomal segmental duplication within the *N. sibirica* genome was conducted. The results revealed that four *NsCDPK* genes are likely products of duplication events, distributed across chromosomes 2, 3, and 10 (Figure 5). Chromosome 3 contains two duplicated segments: NISI03G2595 (532 amino acids) and its shorter counterpart NISI03G2589 (83 amino acids). While NISI03G2589 is identical to the C-terminal region of NISI03G2595, it lacks the complete structural features of a functional CDPK protein. Whereas chromosomes 2 and 10 each possess one duplicated segment. No duplicated *NsCDPK* gene segments were detected on the remaining chromosomes (Figure 5). These findings indicate that segmental duplication has contributed to the expansion of the *NsCDPK* gene family in *N. sibirica*.

**Figure 6:**
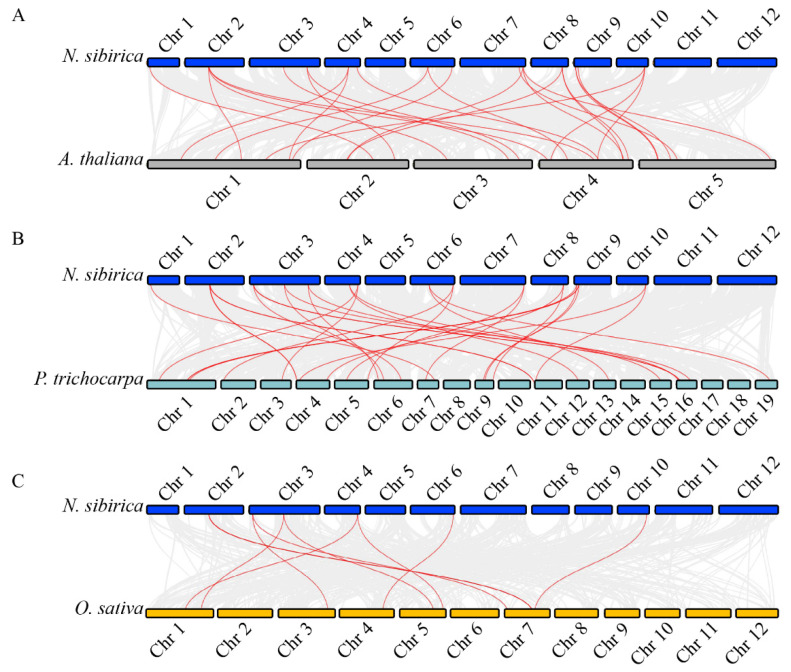
Genome-wide synteny analysis of *CDPK* gene family between *N. sibirica* and other three plant species. (**A**–**C**) Synteny analysis of *CDPK* genes between *N. sibirica* and *A. thaliana* (**A**), *P. trichocarpa* (**B**), and *O. sativa* (**C**), respectively. Blue, yellow, purple and pink bars represent chromosomes of *N. sibirica*, *A. thaliana*, *P. trichocarpa*, and *O. sativa* as indicating, respectively. Gray lines in the background between each two groups of chromosomes mean the collinear blocks between two genomes, while the red lines highlight the syntenic *CDPK* gene pairs.

Additionally, synteny analysis was performed to compare *CDPK* genes between *N. sibirica* and three other plant species: *A. thaliana*, *P. trichocarpa*, and *O. sativa*. A total of 27 orthologous *CDPK* gene pairs were identified between *N. sibirica* and *A. thaliana*, while 31 pairs were detected between *N. sibirica* and *P. trichocarpa*. In contrast, only ten orthologous *CDPK* gene pairs were found between *N. sibirica* and *O. sativa* (Figure 6). These results suggest that *NsCDPK*s share a higher degree of sequence similarity and evolutionary conservation with *CDPK*s from *A. thaliana* and *P. trichocarpa* than with those from *O. sativa*.

### 2.4. Cis-Acting Element Analysis of NsCDPKs Promoters

**Figure 7:**
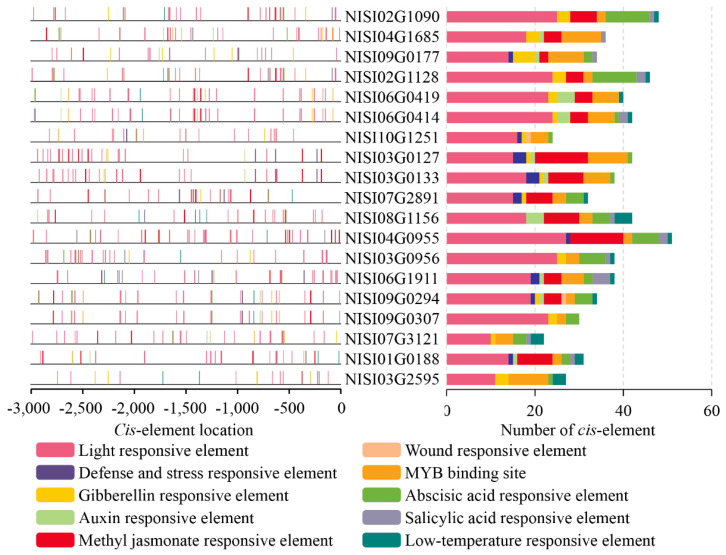
Specific *cis*-elements were identified from the promoter sequence region of *CDPK* genes from *N. sibirica*. The left side of gene ID shows *cis*-acting element distribution in the 3 kb upstream sequences of start codon (zero position) of *NsCDPK*s. *Cis*-acting elements that respond to light, wound, drought, gibberellin (GA), abscisic acid (ABA), auxin, salicylic acid (SA), methyl jasmonate (MeJA) and low-temperature are displayed with differently colored boxes. The right side of gene ID shows the number of *cis*-acting elements identified in the promoter sequences.

*Cis*-acting elements serving as binding sites for trans-acting regulatory factors, play a pivotal role in the initiation and regulation of gene transcription, mediating precise spatial and temporal control of gene expression in response to developmental cues and environmental stimuli [44]. Therefore, investigation of these regulatory elements in *NsCDPK*s is essential for elucidating the functional roles of *CDPK* genes in *N. sibirica* and may provide critical insights into their regulatory mechanisms and biological significance within this species. To analyze the transcriptional regulation of *NsCDPK* genes, 3 kb upstream promoter regions of the open reading frames (ORFs) were extracted from the *N. sibirica* genome for *cis*-acting element analysis. The promoter sequences are provided in Supplementary Data S2. Comprehensive examination of the *NsCDPK* promoter sequences revealed the presence of diverse *cis*-acting elements, including those responsive to light, various phytohormones (such as auxin, ABA, salicylic acid (SA), gibberellin (GA), and methyl jasmonate (MeJA)), as well as elements associated with abiotic stress responses (including wound, drought, and low temperature) (Figure 7). These findings indicate that *NsCDPK* genes are potentially involved in a wide range of regulatory pathways mediating plant responses to environmental stresses.

### 2.5. NsCDPKs Positively Respond to Salt Treatment

**Figure 8:**
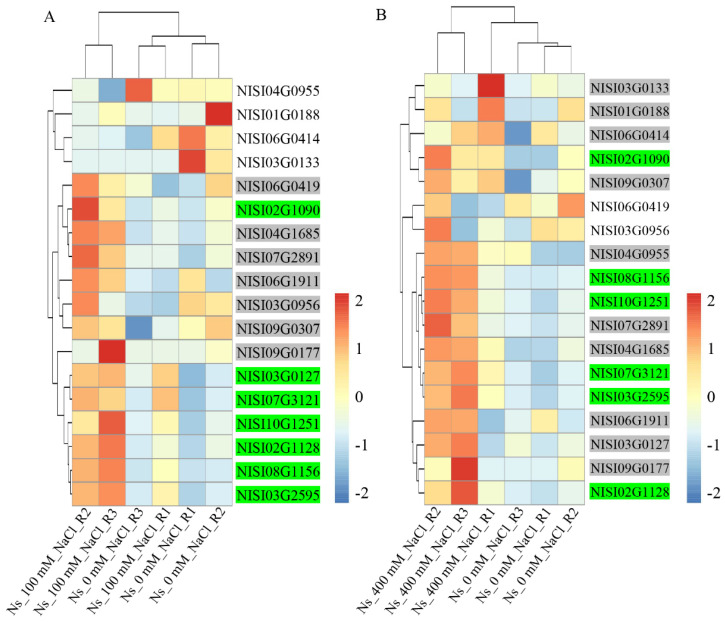
*NsCDPK*s differentially responded to salinity in *N. sibirica* leaves. (**A**,**B**) Clustering analysis (left) and heat map representation (right) of total *CDPK* genes from transcriptome data of *N. sibirica* leaves treated with 100 mM NaCl (**A**) or 400 mM NaCl (**B**) for three days [58]. The gene expression trends are shown as colored boxes with Transcripts per million (TPM) values (TPM = (readCount × 1,000,000)/libsize) scaling by row. The “0” TPM values have been replaced with 0.09–0.1 for scaling by row. “Ns_0 mM_NaCl”, “Ns_100 mM_NaCl” and “Ns_400 mM_NaCl” means 0 mM NaCl, 100 mM NaCl or 400 mM NaCl solution treated *N. sibirica* leaves, respectively. R1–R3 represent three biological repeats. Gene IDs with green shadow represent the significantly upregulated genes under salt treatment. Gene IDs with gray shadow indicate that genes positively responded to salt treatment.

To investigate the response of *NsCDPK* genes to salt stress in *N. sibirica*, transcriptome data from leaves treated with 100 mM and 400 mM NaCl (NCBI accession number: GSE113246) were retrieved and analyzed to profile gene expression changes. The results revealed that the expression patterns of most *NsCDPK*s were altered in response to both 100 mM and 400 mM NaCl treatments compared to the untreated control. Specifically, 14 *NsCDPK*s (~74%) and 16 *NsCDPK*s (~84%) exhibited increased expression following 100 mM and 400 mM NaCl treatments, respectively. Among these, 7 *NsCDPK*s and 6 *NsCDPK*s were significantly upregulated after three days of exposure to 100 mM and 400 mM NaCl, respectively (Figure 8A and 8B). These findings suggest that *NsCDPK* genes may play important roles in mediating *N. sibirica*’s adaptive responses to salt stress.

**Figure 9:**
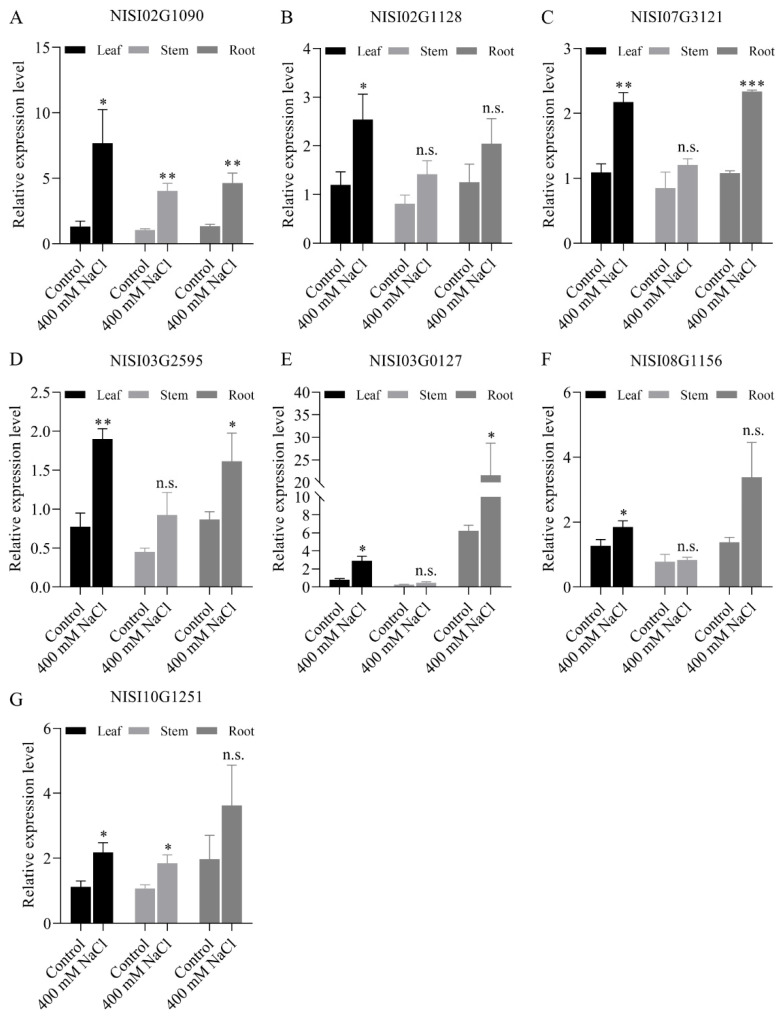
*NsCDP*Ks response to salt in different *N. sibirica* tissues. (**A**–**G**) Quantitative real-time PCR (qPCR) was performed to validate the expression patterns of *NsCDPK* genes that were significantly upregulated according to transcriptome analysis. The expression levels of NISI02G1090 (**A**), NSIS02G1128 (**B**), NSIS07G3121 (**C**), NISI03G2595 (**D**), NISI03G0127 (**E**), NISI08G1156 (**F**), and NISI10G1251 (**G**) were assessed in the leaf, stem, and root tissues of *N. sibirica* following treatment with either 0 mM or 400 mM NaCl for 6 h. Data represent means ± standard deviation (SD) from three biological replicates. One-way ANOVA test was used for statistical analysis, ‘*’ *p* < 0.05, ‘**’ *p* < 0.01, ‘***’ *p* < 0.001, ‘n.s.’ indicates non-significant.

To comprehensively characterize the tissue-specific expression of salt-responsive *NsCDPK*s, transcript levels of the significantly upregulated genes were further examined in the leaves, stems, and roots of *N. sibirica* (Figure 9). The results demonstrated that these *NsCDPK*s exhibited enhanced expression not only in leaves, but also in stems and roots following salt treatment. Notably, the expression levels of NISI02G1090, NSIS07G3121, NISI03G2595, and NISI03G0127 in salt-treated roots were elevated by 4.65-, 2.33-, 1.61-, and 21.57-fold, respectively, compared to leaves under normal conditions, and were significantly higher than those observed in roots under control conditions (Figure 9A,C–E). Similarly, the expression of NISI02G1090 and NISI10G1251 in salt-treated stems increased by 4.04- and 3.63-fold, respectively, relative to leaves under normal conditions, and was also significantly greater than that in stems under control conditions (Figure 9A,G). These findings indicate that the upregulated *NsCDPK*s exhibit a robust and positive response to salt stress across multiple tissues in *N. sibirica*, suggesting their involvement in the species’ adaptive mechanisms to salinity.

### 2.6. Salt-Responsive NsCDPKs Altered Expression in Response to Cold and Drought Treatment

**Figure 10:**
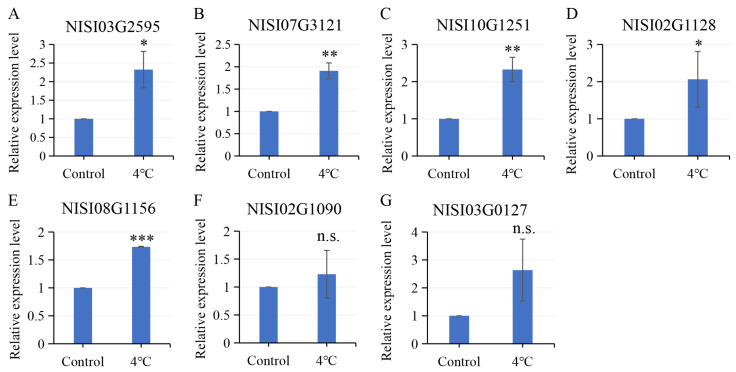
Cold induced positive expression of *NsCDPK*s in *N. sibirica* seedlings. (**A**–**G**) qPCR test was employed to assess the expression levels of salt-responsive *NsCDPK*s including NISI03G2595 (**A**), NISI07G3121 (**B**), NISI10G1251 (**C**), NISI02G1128 (**D**), NISI08G1156 (**E**), NISI02G1090 (**F**) and NISI03G0127 (**G**) in whole *N. sibirica* seedlings subjected to 4 °C treatment for 6 h. Data are presented as means ± standard deviation (SD) from three independent biological replicates. One-way ANOVA test was performed for statistical analysis, ‘*’ *p* < 0.05, ‘**’ *p* < 0.01, ‘***’ *p* < 0.001, ‘n.s.’ indicates non-significant.

To investigate the response of *NsCDPK*s to cold stress, *N. sibirica* seedlings were subjected to 4 °C treatment and subsequently analyzed by qPCR. The *NsCDPK* genes that were upregulated under salt stress also exhibited increased expression in response to cold stress. Specifically, the expression levels of NISI03G2595, NISI07G3121, NISI10G1251, NISI02G1128, and NISI08G1156 were significantly elevated following cold treatment (Figure 10A–E). Compared to seedlings under normal conditions, these genes showed 2.32-, 1.91-, 2.33-, 2.06-, and 1.73-fold increases in expression, respectively (Figure 10A–E). NISI02G1090 and NISI03G0127 also show positive response to cold treatment in *N. sibirica* seedlings (Figure 10F,G). These findings suggest that *NsCDPK*s might play a positive regulatory role in the response of *N. sibirica* to cold stress, making these respective *CDPK*s interesting candidates for further studies on mechanism in dealing with cold.

**Figure 11:**
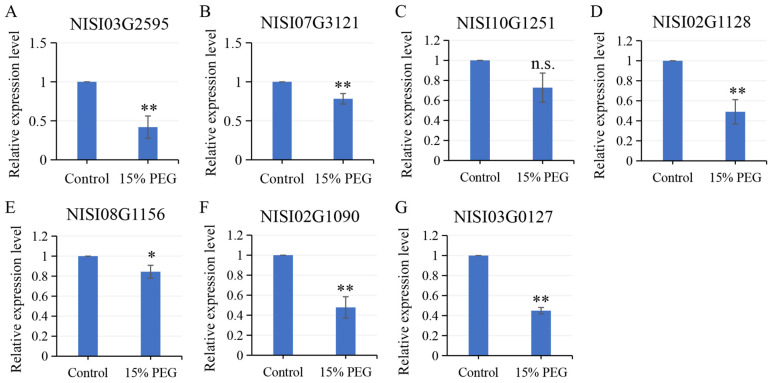
Drought induced negative expression of *NsCDPK*s in *N. sibirica* seedlings. (**A**–**G**) qPCR test was employed to assess the expression levels of salt-responsive *NsCDPK*s including NISI03G2595 (**A**), NISI07G3121 (**B**), NISI10G1251 (**C**), NISI02G1128 (**D**), NISI08G1156 (**E**), NISI02G1090 (**F**) and NISI03G0127 (**G**) in whole *N. sibirica* seedlings subjected to 15% PEG6000 treatment for 6 h. Data are presented as means ± standard deviation (SD) from three independent biological replicates. One-way ANOVA test was performed for statistical analysis, ‘*’ *p* < 0.05, ‘**’ *p* < 0.01, ‘n.s.’ indicates non-significant.

To investigate whether these salt-responsive *CDPK*s also contribute to drought adaptation of *N. sibirica*, we examined the expression profiles of them in *N. sibirica* seedlings subjected to drought stress, simulated by treatment with 15% PEG6000. The results indicated that the expression levels of the analyzed *NsCDPK*s were significantly downregulated under drought conditions (Figure 11). Specifically, the relative expression levels of NISI03G2595, NISI07G3121, NISI02G1128, NISI08G1156, NISI02G1090, and NISI03G0127 in drought-treated seedlings were reduced to 0.42, 0.78, 0.49, 0.84, 0.48, and 0.45 times those observed in seedlings under normal conditions, respectively (Figure 11A,B,D,E, and G). These findings reveal contrasting expression patterns of *NsCDPK*s in response to different abiotic stresses, suggesting that these genes may have distinct roles in mediating physiological adaptation to diverse environmental challenges in *N. sibirica*.

### 2.7. Identification of Candidate Transcription Factors Regulating NISI02G1090 Transcription

**Figure 12:**
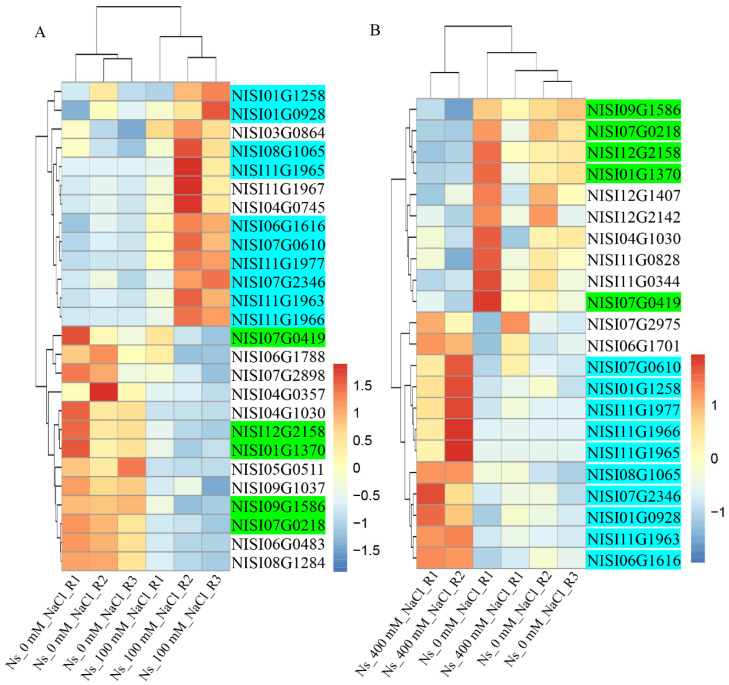
*NsMYB*s exhibited a various response to different salinity levels in *N. sibirica* leaves. (**A**,**B**) Clustering analysis (left) and heat map representation (right) of significantly up- and downregulated *MYB* genes from transcriptome data of *N. sibirica* leaves treated with 100 mM NaCl (**A**) or 400 mM NaCl (**B**) for three days [58]. The gene expression trends are shown as colored boxes with Transcripts per million (TPM) values (TPM = (readCount × 1,000,000)/libsize) scaling by row. The “0” TPM values have been replaced with 0.09–0.1 for scaling by row. “Ns_0 mM_NaCl”, “Ns_100 mM_NaCl” and “Ns_400 mM_NaCl” means 0 mM NaCl, 100 mM NaCl or 400 mM NaCl solution treated *N. sibirica* leaves, respectively. R1–R3 represent three biological repeats. Blue gene IDs denote genes exhibiting a significantly positive response to salt treatment, while green gene IDs represent genes that are negatively regulated under salt stress conditions.

*Cis*-acting element analysis revealed that the promoters of *NsCDPK*s contain 2 to 9 MYB binding sites, suggesting potential regulation of *NsCDPK*s by NsMYBs (Figure 7). To further investigate the functional roles of NsMYBs in modulating the expression of salt-responsive *NsCDPK*s, a total of 105 *NsMYB* genes were identified from the *N. sibirica* genome. Transcriptome data from *N. sibirica* leaves were utilized to analyze their expression patterns under salt stress, revealing that *NsMYB*s exhibit differential responses to salt treatment (Supplementary Figure S1). Specifically, the expression level of 26 and 22 *NsMYB*s was differentially expressed in leaves exposed to 100 mM and 400 mM NaCl, respectively (Figure 12). Among these, 10 *NsMYB*s were significantly upregulated and 5 *NsMYB*s were significantly downregulated under both salt concentrations, indicating their potential involvement in the salt tolerance mechanisms of *N. sibirica* (Figure 12).

**Figure 13:**
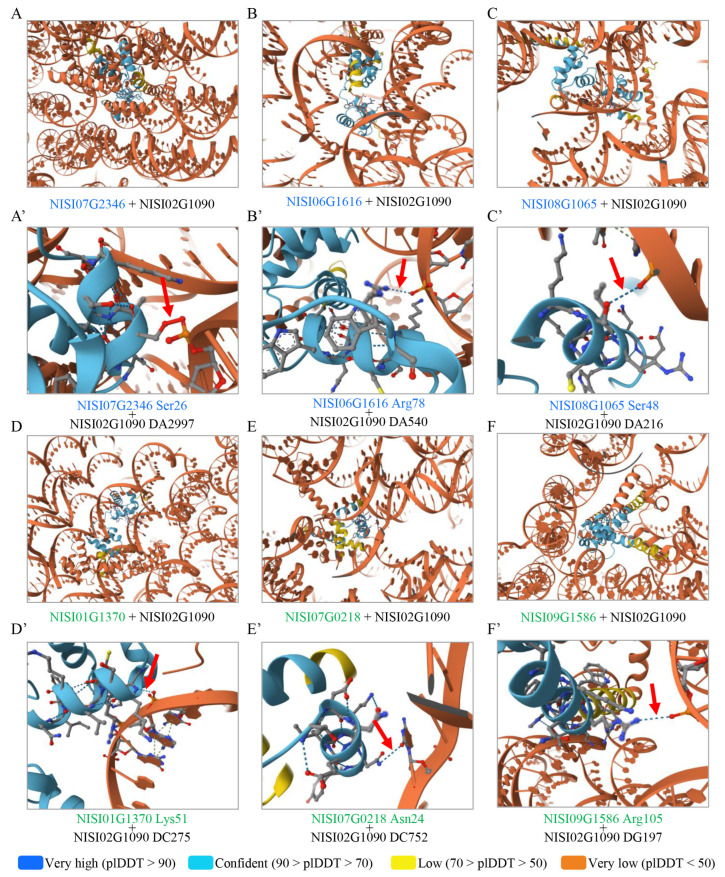
Predicted binding of salt-responsive NsMYB to the *NsCDPK* promoter. (**A**–**F**) Structural models generated using AlphaFold illustrate the interaction between NsMYB transcription factors and the *NsCDPK* (NISI02G1090) promoter. (**A’**–**F’**) The chemical bonds between the NsMYB protein and the *NsCDPK* promoter corresponding to panels (**A**–**F**) are indicated with red arrowheads. The predicted local difference distance test (pLDDT) scores were generated by AlphaFold Server. Blue gene IDs indicate upregulated expressing *NsMYB*s. Green gene IDs indicate downregulated expressions of NsMYBs.

To further elucidate the potential interactions between salt-responsive *NsMYB*s and *NsCDPK* promoters, the protein sequences of the 15 differentially expressed *NsMYB*s and the promoter sequence of one *NsCDPK* gene (NISI02G1090) were analyzed using the AlphaFold Server (Supplementary Figure S2). The results demonstrated that the NsMYBs could potentially bind to the NISI02G1090 promoter sequence ((Supplementary Figure S2, Figure 13). Collectively, these findings suggest that NsMYBs may act as key upstream regulators of salt-responsive *NsCDPK*s, providing new insights into the molecular mechanisms underlying the extreme salt tolerance observed in *N. sibirica*.

## 3. Discussion

CDPKs are pivotal signaling components in plants, mediating responses to diverse abiotic stresses such as salinity, drought, and cold. In this study, the transcriptomic analysis of *NsCDPK* genes in *N. sibirica* revealed dynamic changes in their expression profiles under salt, cold, and drought stress conditions. These findings provide valuable insights into the molecular mechanisms underlying the adaptive responses of *N. sibirica* to environmental challenges, highlighting the potential roles of *NsCDPK*s in stress tolerance.

### 3.1. NsCDPKs Responded to Various Abiotic Stresses in N. sibirica

The results of this study demonstrated that most *NsCDPK*s exhibited altered expression patterns in response to salt stress, with a majority showing significant upregulation following treatment with 100 mM and 400 mM NaCl. Notably, several *NsCDPK*s, including NISI02G1090, NSIS07G3121, NISI03G2595, and NISI03G0127, displayed robust expression across multiple tissues, such as leaves, stems, and roots. This tissue-specific response suggests that *NsCDPK*s are involved in coordinating systemic adaptive mechanisms to salinity. The enhanced expression of *NsCDPK*s in roots under salt stress, particularly the dramatic upregulation of NISI03G0127 (21.57-fold), implies their critical role in root-mediated salt tolerance. Roots are the primary site of salt perception and ion uptake, and the activation of *NsCDPK*s in this tissue may facilitate ion homeostasis and osmotic adjustment. Similarly, the upregulation of NISI02G1090 and NISI10G1251 in stems suggests their involvement in vascular tissue responses, potentially aiding in the transport of ions and osmolytes to maintain cellular homeostasis.

These findings align with previous studies on *CDPK*s in other plants, such as wheat and oat, which demonstrated the importance of *CDPK*s in regulating salt stress-related pathways, including ion transport and reactive oxygen species (ROS) scavenging [59,60]. *CDPK*s are activated by calcium influxes triggered by abiotic stress signals, leading to phosphorylation of target proteins involved in stress responses. Under salt stress, NsCDPKs may phosphorylate ion transporters such as high-affinity potassium transporters (HKT) and salt overly sensitive 1 (SOS1), facilitating ionic balance and salt tolerance. Additionally, NsCDPKs might regulate ROS-scavenging enzymes, such as superoxide dismutase (SOD) and catalase (CAT), to mitigate oxidative damage caused by salt stress. Recent findings have revealed that OsCDPK5 and OsCDPK13 could activate key mitogen-activated protein kinase (MAPK) involved in stress signaling to enhance plant salt tolerance, thereby contributing to its adaptive response to high salinity environments [61].

Cold stress is another major abiotic factor affecting plant growth and development. The upregulation of salt-responsive *NsCDPK*s under cold stress suggests their multifunctional roles in abiotic stress adaptation. Specifically, genes such as NISI03G2595, NISI07G3121, and NISI10G1251 exhibited significant increases in expression following exposure to 4 °C, indicating their involvement in cold-induced signaling pathways. CDPKs are known to regulate cold stress responses by modulating calcium signaling and activating downstream transcription factors (TFs). *Arabidopsis* CDPK28 has been reported to phosphorylate and facilitate the nuclear translocation of NIN-LIKE PROTEIN 7 (NLP7), a transcription factor responsible for regulating cold-responsive gene sets. This mechanism allows *Arabidopsis* to integrate cold-induced calcium signals with transcriptional changes, enabling an efficient and rapid response to cold stress [20]. Therefore, the observed upregulation of *NsCDPK*s under cold stress may similarly activate cold-responsive TFs, thereby enhancing the expression of genes associated with cold tolerance, such as those involved in membrane stabilization and ROS detoxification.

### 3.2. The Prediction of Potential Upstream TF Regulating NsCDPKs

*Cis*-acting elements, as binding sites for trans-acting regulatory factors, are crucial for the precise spatial and temporal regulation of gene transcription in plants. These elements mediate the integration of developmental cues and environmental stimuli into gene expression networks, enabling plants to adapt dynamically to changing environmental conditions [44]. The identification and functional characterization of *cis*-acting elements in promoters of *CDPK* genes provide critical insights into how these genes contribute to abiotic stress responses. *CDPK*s are well-established as key mediators of calcium signaling, transducing environmental signals into cellular responses by phosphorylating downstream targets [62,63]. In halophytes like *N. sibirica*, which thrive in extreme environments, *CDPK*s are hypothesized to play pivotal roles in mediating physiological adaptations to abiotic stresses such as salinity, drought, and cold stress.

The analysis of 3 kb upstream promoter regions of *NsCDPK* genes revealed a diverse array of *cis*-acting elements associated with light responses, phytohormone signaling and abiotic stress responses. These findings indicate that *NsCDPK*s are integrated into multiple regulatory pathways, enabling *N. sibirica* to respond effectively to environmental challenges. Notably, the presence of 2 to 9 MYB binding sites in the promoters of *NsCDPK*s suggests a potential regulatory interaction between NsMYB proteins and *NsCDPK* genes, forming a transcriptional network that may underlie stress tolerance mechanisms. MYB transcription factors are widely recognized as key regulators of abiotic stress responses, particularly in modulating gene expression during salt and drought stress [64,65]. The identification of MYB binding sites in *NsCDPK* promoters provides a foundation for exploring the hierarchical regulatory networks that govern abiotic stress responses in *N. sibirica*. The upregulation of *NsMYB*s under salt stress suggests that these transcription factors may activate *NsCDPK*s to mediate calcium signaling, thereby enhancing salt tolerance. Conversely, the downregulation of certain *NsMYB*s may reflect feedback inhibition mechanisms that fine-tune stress responses to prevent excessive energy expenditure under prolonged stress conditions.

Future research should focus on elucidating the downstream targets of *NsCDPK*s under different stress conditions to identify the specific signaling pathways they regulate. Functional characterization of NsMYBs and their interactions with *NsCDPK* promoters will provide deeper insights into the transcriptional networks underlying salt stress tolerance. Additionally, comparative studies of *NsCDPK*s and *MYB*s in other halophytes and glycophytes may reveal conserved and species-specific mechanisms of abiotic stress adaptation. Such studies will not only advance our understanding of stress signaling in halophytes but also inform strategies for improving stress tolerance in crop species through genetic engineering or breeding.

## 4. Materials and Methods

### 4.1. Plant Materials and Treatments

*N. sibirica* seeds were generously provided by the Experimental Center for Desert Forestry, Chinese Academy of Forestry. Seeds were stratified in moist sand at 4 °C for two months and subsequently germinated in soil-filled pots under controlled conditions. One-month-old seedlings were subjected to stress treatments with 400 mM NaCl, 15% polyethylene glycol (PEG6000), or exposure to 4 °C for 6 h. Following each treatment, samples were immediately flash-frozen in liquid nitrogen and stored at −80 °C for RNA extraction. For analysis of tissue-specific expression of *NsCDPK*s under salt stress, roots, stems, and leaves were harvested separately from seedlings treated with 400 mM NaCl. For drought and cold treatments, whole seedlings were collected for gene expression analysis. Seedlings grown under normal conditions without stress were harvested as controls. Each treatment included three plants as independent biological replicates.

### 4.2. Identification of N. sibirica CDPK Family Members

To identify CDPK proteins in *N. sibirica*, the protein sequences of *A. thaliana* CDPKs (AtCDPKs) were retrieved from the TAIR database and used as query sequences for BLASTP searches against the *N. sibirica* proteome. Candidate CDPK sequences with significant similarity to AtCDPKs were collected for further analysis. Multiple sequence alignment of the putative CDPK proteins was performed using DNAMAN Version 9 software to assess sequence similarity and ensure sequence integrity. To confirm the presence of characteristic CDPK domains, conserved domain verification was conducted using the NCBI CD-search tool. Furthermore, conserved motif analysis was performed using the MEME Suite 5.5.7 online software (http://meme-suite.org/tools/meme, accessed on 1 March 2025) [66], with the maximum number of motifs set to ten and all other parameters set to default values, to identify typical CDPK motifs within the candidate sequences. Through this comprehensive approach, a total of 19 *CDPK* genes were successfully identified in the *N. sibirica* genome.

### 4.3. Basic Information of NsCDPK Proteins

The physicochemical properties of the identified NsCDPK proteins, including amino acid (AA) length, molecular weight (MW), isoelectric point (pI), instability index (II), aliphatic index (AI), and grand average of hydropathicity (GRAVY), were analyzed using the ProtParam tool available on the ExPASy website (https://web.expasy.org/protparam/, accessed on 17 January 2023) [67]. For secondary structure prediction, the SOPMA online software (http://npsa-pbil.ibcp.fr/cgi-bin/npsa_automat.pl?page= npsa_sopma.html, accessed on 17 January 2023) was employed, following the default parameters [68].

### 4.4. Phylogenetic Study for NsCDPK Proteins

For evolutionary analysis, 34 CDPK protein sequences of *A. thaliana* were retrieved from the TAIR database (https://www.arabidopsis.org/, accessed on 17 January 2023), sequences of *O. sativa*, *Z. mays*, *V. vinifera*, and *P. trichocarpa* CDPKs were obtained from Phytozome (http://www.phytozome.net/, accessed on 17 January 2023), respectively; 19 CDPK protein sequences from *N. sibirica* were obtained as described above. The full list of NsCDPK protein sequences is provided in Supplementary Data S1. Phylogenetic relationships among these CDPKs were inferred using the “One Step Build a ML Tree” function in TBtools [57] with default parameters. The resulting maximum likelihood tree was subsequently visualized and annotated using the Interactive Tree of Life (iTOL) online tool [69].

### 4.5. Collinearity Analysis of CDPKs Between N. sibirica and Other Plants

Genome-wide synteny analysis of *CDPK* genes in *N. sibirica* was conducted using TBtools-II v2.357 software to investigate homologous relationships among *CDPK* family members [57]. Genome sequences for *A. thaliana*, *P. trichocarpa*, and *O. sativa* were obtained from the Phytozome 13 database (https://phytozome-next.jgi.doe.gov/, accessed on 19 January 2023). Collinearity analyses between *NsCDPK*s and their homologous counterparts in *A. thaliana*, *P. trichocarpa*, and *O. sativa* were performed using the “One Step MCScanX” function within the Synteny Visualization module of TBtools, employing default parameters [57].

### 4.6. Cis-Acting Element Analysis of NsCDPK Promoters

To investigate the *cis*-acting regulatory elements within *NsCDPK* promoter regions, TBtools was employed to extract 3000 bp sequences upstream of the open reading frames (ORFs) of *NsCDPK* genes from the *N. sibirica* genome (Supplementary Data S2). The retrieved promoter sequences were subsequently analyzed for *cis*-acting elements using the PlantCARE database (http://bioinformatics.psb.ugent.be/webtools/plantcare/html/, accessed on 31 January 2023) [44]. The distribution of identified *cis*-acting elements within the promoter regions was visualized using TBtools, while the frequency of each element type was summarized and plotted using Microsoft Excel.

### 4.7. Transcriptome Data Analysis

To investigate the transcriptional responses of *NsCDPK* and *NsMYB* genes to salt stress, transcriptome data from *N. sibirica* leaves subjected to 100 mM or 400 mM NaCl treatment for three days were obtained from the NCBI Gene Expression Omnibus (accession number: GSE113246) [58]. Gene expression levels were quantified as transcripts per million (TPM) using Kallisto [70]. The expression profiles of *NsCDPK* and *NsMYB* genes under both salt stress and control conditions were visualized as heatmaps, illustrating the dynamic changes in gene expression in response to salt treatment.

### 4.8. Quantitative Real-Time PCR Analyses

Quantitative real-time PCR (qPCR) was performed to evaluate the transcriptional responses of *NsCDPK* genes to various abiotic stresses. Total RNA was isolated from one-month-old *N. sibirica* seedlings subjected to 400 mM NaCl, 15% polyethylene glycol (PEG 6000), or 4 °C for 6 h, using the Eastep^®^ Super Total RNA Purification Kit (Promega, Shanghai, China) according to the manufacturer’s protocol. Genomic DNA contamination was eliminated by DNase I treatment included in the extraction kit. RNA integrity was assessed by agarose gel electrophoresis, and concentrations were determined using ultraviolet spectrophotometry. High-quality RNA free of DNA contamination was used for first-strand cDNA synthesis with the HiScript III 1st Strand cDNA Synthesis Kit (+gDNA wiper) (Vazyme Biotech, Nanjing, China). qPCR assays were conducted on a LightCycler^®^ 480 system (Roche, Basel, Switzerland) using TB Green^®^ Premix Ex Taq™ (Takara, Dalian, China), following the manufacturer’s instructions. Gene expression levels were normalized against the *N. sibirica* actin gene [71]. Each target gene was analyzed in three independent biological replicates using gene-specific primers listed in Supplementary Table S1. Plants with consistent growth were selected for all treatments, and three plants were used for each treatment as biological replicates. Each biological replicate included two technical replicates.

### 4.9. The Prediction for the Binding of NsMYB to NsCDPK Promoter

To investigate the potential regulatory interactions between salt-responsive *N. sibirica* MYB transcription factors and *NsCDPK* gene promoters under salt stress, we employed a structure-based prediction approach using the AlphaFold 3 Server (https://alphafoldserver.com/, accessed on 14 August 2025) [72,73,74]. Protein sequences of 15 differentially expressed NsMYBs, identified through transcriptomic analysis, were retrieved and used for subsequent interaction prediction. The promoter sequence of a representative salt-responsive *NsCDPK* gene, NISI02G1090, was selected based on its significant transcriptional upregulation under salt stress conditions.

Protein-DNA interaction predictions were performed by submitting the full-length amino acid sequences of the 15 NsMYBs and the promoter sequence of NISI02G1090 to the AlphaFold Server. The server utilizes advanced deep-learning algorithms to model protein structures and predict their potential binding interfaces with nucleic acids. The predicted interactions were visualized and analyzed to assess the binding affinities and specificity of each MYB protein toward the *NsCDPK* promoter.

## 5. Conclusions

In summary, this study presents the comprehensive analysis of the *CDPK* gene family in the halophyte *N. sibirica*, revealing its structural diversity, evolutionary conservation, and multifaceted regulatory roles in abiotic stress adaptation. The identification of a potential regulatory module involving MYB transcription factors and *CDPK*s might offer new insights into the molecular mechanisms underlying extreme salt tolerance. These findings provide a foundation for future functional studies and highlight the potential of *N. sibirica* as a model for investigating stress signaling pathways in halophytes.

## Figures and Tables

**Table 1:** Characteristics of NsCDPK proteins.

Gene ID	AA	MW (kDa)	pI	II	AI	GRAVY
NISI02G1090	979	110.78	5.90	32.41	83.09	−0.356
NISI04G1685	532	59.77	6.02	37.06	77.74	−0.474
NISI09G0177	530	58.77	5.72	37.15	76.75	−0.493
NISI02G1128	509	57.04	6.18	31.45	81.45	−0.412
NISI06G0419	500	56.20	5.51	39.71	88.14	−0.296
NISI06G0414	501	56.23	5.51	39.80	88.34	−0.296
NISI10G1251	547	61.59	6.35	42.80	74.41	−0.559
NISI03G0127	608	68.41	5.68	44.52	78.87	−0.460
NISI03G0133	608	68.41	5.68	44.52	78.87	−0.460
NISI07G2891	498	56.00	5.35	39.43	83.39	−0.374
NISI08G1156	568	63.71	5.72	41.85	80.18	−0.448
NISI04G0955	554	63.00	6.99	39.95	85.00	−0.459
NISI03G0956	527	59.53	6.03	37.86	83.23	−0.449
NISI06G1911	880	98.07	6.86	42.00	89.07	−0.311
NISI09G0294	531	59.66	6.40	32.95	81.90	−0.470
NISI09G0307	531	59.66	6.40	32.95	81.90	−0.470
NISI07G3121	574	65.28	9.25	42.30	76.11	−0.695
NISI01G0188	542	61.95	5.89	38.26	79.67	−0.491
NISI03G2595	532	60.62	6.78	36.20	81.35	−0.536

The identities of NsCDPK proteins were identified on the ExPASy website (https://web.expasy.org/protparam/, accessed on 17 January 2023). AA: Amino acid; MW: Molecular weight; pI: Isoelectric point; II: Instability index; AI: Aliphatic index; GRAVY: Grand average of hydropathicity.

**Table 2:** Secondary structure prediction of NsCDPK proteins.

Gene ID	Alpha Helix%	Extended Strand%	Beta Turn%	Random Coil%
NISI02G1090	48.31	11.85	9.70	30.13
NISI04G1685	41.35	9.40	7.89	41.35
NISI09G0177	43.02	9.43	8.30	39.25
NISI02G1128	44.60	10.02	9.23	36.15
NISI06G0419	47.00	10.60	9.40	33.00
NISI06G0414	46.91	10.18	8.78	34.13
NISI10G1251	40.77	8.96	8.59	41.68
NISI03G0127	40.30	9.54	7.57	42.60
NISI03G0133	40.30	9.54	7.57	42.60
NISI07G2891	45.58	13.25	9.44	31.73
NISI08G1156	40.14	10.56	8.10	41.20
NISI04G0955	43.50	10.83	8.66	37.00
NISI03G0956	47.63	10.63	8.35	33.40
NISI06G1911	36.25	15.45	8.98	39.32
NISI09G0294	45.76	10.17	8.85	35.22
NISI09G0307	45.76	10.17	8.85	35.22
NISI07G3121	40.42	12.20	7.14	40.24
NISI01G0188	43.54	10.89	8.86	36.72
NISI03G2595	47.74	10.53	8.83	32.89

The secondary structures of NsCDPK proteins were predicted Via the SOPMA database (http://npsa-pbil.ibcp.fr/cgi-bin/npsa_automat.pl?page=npsa_sopma.html, accessed on 17 January 2023).

## Data Availability

The transcriptome data of *N. sibirica* leaves subjected to 100 mM or 400 mM NaCl treatment for three days were obtained from the NCBI Gene Expression Omnibus (accession number: GSE113246) [58] (https://www.ncbi.nlm.nih.gov/bioproject/PRJNA679101/, accessed on 7 August 2022).

## Supplementary Materials

The following supporting information can be downloaded at: https://www.mdpi.com/article/10.3390/plants14193091/s1, Supplementary Table S1: Specific primers of *NsCDPK*s for quantitative real-time PCR analysis. Supplementary Data S1: Accession number and protein sequences of the NsCDPKs identified from *N. sibirica* genome in this study. Supplementary Data S2: The promoter sequences (3 kb) of the *NsCDPK*s isolated from *N. Sibirica* genome. Supplementary Figure S1: Heatmaps illustrating differential expression patterns of *NsMYB* genes in response to salt stress in *N. sibirica*. Supplementary Figure S2: Predicted binding of salt-responsive NsMYB on *NsCDPK* promoter.
