# Supplementary material for: Classification of Calcium-Dependent Protein Kinases and Their Transcriptional Response to Abiotic Stresses in Halophyte Nitraria sibirica
Source: Plants (Basel). 2025 Oct 7;14(19):3091. doi: 10.3390/plants14193091 (PMC12526309; doi:10.3390/plants14193091)
Supplement: Supplementary file 1 [file plants-14-03091-s001.zip › Supplementary data.pdf]

Supplementary Table S1. Specific primers of *NsCDPK*s for quantitative real-time PCR analysis

Accession number	Primer name	Sequence (5'-3')
NISI02G1090	NISI02G1090F	AAAACCCTTTGAAGATGTTAAGGCGTA
	NISI02G1090R	AAAGATATGTTACCCCAAATTGTCCC
NISI02G1128	NISI02G1128F	AACTTGCTCTTAAGGTTATCGCAGA
	NISI02G1128R	TCCTGCCTTCAGTTCATCATAGGTG
NISI03G0127	NISI03G0127F	GCAGCCCTCTAGTTATAACAATGTGGA
	NISI03G0127R	TTTCCTAGAATGTGCCCGCCTA
NISI03G2595	NISI03G2595F	CGCCTTATCTGATGAAATCGACACC
	NISI03G2595R	CCTCATAACTTATTCTTCCGTCCT
NISI07G3121	NISI07G3121F	TACATCAAATGGAGGAACACGACT
	NISI07G3121R	TCAAACCCGTATGCATTCTAAGCTC
NISI08G1156	NISI08G1156 F	TAGCTTGCGCCGAACATAATATGACA
	NISI08G1156 R	GCATCATAGCGACAAATTCACCGTA
NISI10G1251	NISI10G1251F	CGTCAGACAAGCCGATTGACA
	NISI10G1251R	TTTCAGCAATCACCTTTAACGCAAG
NISI07G0821	NISI07G0821F	TACAGTGTCATCACAATCGAGAGGC
	NISI07G0821R	AACTCCAGATGACACAAGGCAAG

NISI07G0821, the *N.sibirica* actin gene, was taken as the reference gene.

Supplementary Data S1. **Accession number and protein sequences of the NsCDPKs identified from *N. sibirica* genome in this study**

>NISI02G1090

MGNCLIGDSKATGNVHHVRTQERPTTAASHPAASPPSKPAAHHHRHDSILGKPFEDVKA
YYSLGKELGRGQFGVTYLCTEISTGKKLACKSISKRKLVTRSDKEDMRREIQIMQHLSG
QPNIVEFKGTYEDKDSVNLVMELCVGGELFDRIIAKGHYSERAAASICRAIVNVVNVCH
FMGVMHRDLKPENFLLSSKDENAVLKAIDFGLSVFIEEGKVYKDIVGSAYYVAPEVLRR
KYDKEIDVWSAGVILYILLSGVPPFWAETERGIFDAIMKGKIDFESQPWPSISNSAKDLVR
KMLTQDPKKRITSAQVLEHPWLREGGEASDKPIDSAVLSRMKQFRAMNKLKKLALKVI
AENLSSDEIQGLKAMFTNMDTDNSGTITYDELKAGLARLGSKLTEAEVQQLMEAADVD
GNGTIDYIEFITATMHRHRLERDEHLYKAFQHFDKDNSGFITVDELKTAMKEYGMGDD
DTIREIIAEVDTDNDGRINYDEFCAMMKGGNPQQAKLFYGQFGVTYLCTDISTGKKLAC
KSISKRKLVIRSDKEYMRREMQIMQHEIIAKGHYSERAAASICRAIVNVVNVCHFMGVM
HRDLKPENFLLSSKDENAVLKATDFRLSMFIDEDKFSCEICFWLKQSKELSTAETNSWIC
PYLSDYTLVLSGKVYKDIVGSAYYVAPEVLRRKYDKEIDVWSAGVILYILLSGVPPFWA
ETERGIFDAIMKGEIDFESQPWPSISNSAKNLVRKMLTQDPKNRITFAQVLEHPWLREGG
EALDKPIDSDVLSRMKQFWAMNKLKKLALKVIAENLSSDEIQGLKAMFMNMDTDNSD
TITYDELKAGLARLGSKLTEAEVQQLMEAADVDGNGTIDYIEFITATMHRHRLERDEHL
YKAFQHFDKDNSGFITVDELKTAMKEYGMGNDDTIREIIAEVNIDNVRDSEVFSFFGSLL
QHHFSNMLCLQDGRINYDEFCAMVKGGNPQQAKLF

>NISI04G1685

MGNCNGLPSSENELHSTVPATTATAPASASKTPTDSHTAPQHKSNEHRIHRPAPSTNTGA
ATSAPIGRVLGKPVEDVRSTYVFGRELGRGQFGVTYLVTHKETKQQFACKSIATRKLIN
RDDVEDVRREVQIMHHLTGHRNIVELKAAYEDRHSVNLIMELCGGGELFDRIIAKGHYS
ERAAANLCRQMVTVVHNCHSMGVMHRDLKPENFLFLSSDENSPLKATDFGLSVFFKPG
DVFKDLVGSAYYVAPEVLRRSYGAEADIWSAGVILYILLSGVPPFWAENEQGIFDAILRG
HIDFSTDPWPSISTSAKDLVKKMLRADARERLSANEVLNHPWVREDGDASDKPLDIAVL
SRMKQFRAMNKLKKVALKVIAENLSEEEIVGLKEMFKSMDTDNSGTITFEELKAGLPKL
GTKISESEVRQIMEAADVDGNGTIDYIEFITATMHMNRMEREEHLYKAFEYFDKDKSGY
ITMEELEHALKEYNMGDEKTIKEIIAEVDTDHDGRINYDEFVAMMRKGNPDMVKNRRR
K

>NISI09G0177

MGNCCSKGSEDDKGEATTGNGPNNRAGTGGNEGSTAHQKAAPTASPPGTSTKPAKTGP
IGPVLGRPMEDVKATYSIGKELGRGQFGITHSCTHKGTGEHFACKTIAKRKLVNKEDIED
VRREVQIMHHLTGQPNIVELKGAYEDKQSVHLVMELCAGGELFDRIIAKGHYTERAAAS
LLRTIVQIVHTCHSMGVIHRDLKPENFLLLNKEENSPLKATDFGLSVFYKPGEVFKDIVG
SAYYIAPEVLKRRYGPEADIWSVGVMLYILLSGVPPFWAESEHGIFNAILRGHVDFSSDP
WPQISPQAKDLVKKMLNSDPKQRLTAYQVLNHPWIKEDGEAPDVPLDNAVLSRLKQFK
AMNKFKKVALRVIAGCLSEEEIMGLKEMFRAMDTDNSGTITLEELKTGLAKQGTKLSEF

EVKQLMEAADADGNGTIDYDEFIAATMHLNRMDKEEHLYTAFQHFDKDNSGFITTEEL
EQVLREYGMNDGKDIMEIISEVDGDNDGRINYDEFVAMMRKGNPEANPKKRRDVEINI

>NISI02G1128

MGNCLIGDSKATGNVHHVRTQERPTTAASHPAASPPSKPAAHHHRHDSILGKPFEDVKA
YYSLGKELGRGQFGVTYLCTEISTGKKLACKSISKRKLVTRSDKEDMRREIQIMQHLSG
QPNIVEFKGTYEDKDSVNLVMELCVGGELFDRIIAKGHYSERAAASICRAIVNVVNVCH
FMGVMHRDLKPENFLLSSKDENAVLKAIDFGLSVFIEEGKVYKDIVGSAYYVAPEVLRR
KYDKEIDVWSAGVILYILLSGVPPFWAETERGIFDAIMKGKIDFESQPWPSISNSAKDLVR
KMLTQDPKKRITSAQVLEHPWLREGGEASDKPIDSAVLSRMKQFRAMNKLKKLALKVI
AENLSSDEIQGLKAMFTNMDTDNSGTITYDELKAGLARLGSKLTEAEVQQLMEAADVD
GNGTIDYIEFITATMHRHRLERDEHLYKAFQHFDKDNSGFITVDELKTAMKEYGMGDD
DTIREIIAEVDTDNDGRINYDEFCAMMKGGNPQQAKLF

>NISI06G0419

MKKQTTGASLSTTKPSNTVLPYQTPRLRDHYLLGKKLGQGQFGTTYLCTHKVTNALFA
CKSIPKRKLLCREDYDDVWREIQIMHHLSEHPHVVRIQGTYEDSVFVHLVMELCGGGEL
FDRIVAKGHYSERQAAELIKTIVGVVEACHSLGVMHRDLKPENFLFDSPADDSKLKATD
FGLSVFYKPGQKFSDVVGSPYYVAPEVLLKHYGPEIDVWSAGVILYILLSGVPPFWAETE
SGIFKQILKGKLDFESEPWPSISDSAKDLIRKMLERDPRSRISAHEVLCHPWIVDDTVAPD
QPLDSAVLSRLKHFSAMNKLKKMALRVIAERLSEEEIGGLKELFKMIDTDNSGTITFEEL
KVALKRVGSELMESEIKSLMEAADVDNSGTIDYGEFLAATLHLNKIEREDNLVAAFSYF
DKDGSGYITIDEIQHACKEFGLGDVHLDEVIKEIDQDNDGRIDYGEFAAMMRKGDPGIG
RNRTMRSNLNFNIADAFAEKTTITE

>NISI06G0414

MKKQTTGASLSTTKPSNTVLPYQTPRLRDHYLLGKKLGQGQFGTTYLCTHKVTNALFA
CKSIPKRKLLCREDYDDVWREIQIMHHLSEHPHVVRIQGTYEDSVFVHLVMELCGGGEL
FDRIVAKGHYSERQAAELIKTIVGVVEACHSLGVMHRDLKPENFLFDSPADDSKLKATD
FGLSVFYKPGQKFSDVVGSPYYVAPEVLLKHYGPEIDVWSAGVILYILLSGVPPFGQVET
ESGIFKQILKGKLDFESEPWPSISDSAKDLIRKMLERDPRSRISAHEVLCHPWIVDDTVAP
DQPLDSAVLSRLKHFSAMNKLKKMALRVIAERLSEEEIGGLKELFKMIDTDNSGTITFEE
LKVALKRVGSELMESEIKSLMEAADVDNSGTIDYGEFLAATLHLNKIEREDNLVAAFSY
FDKDGSGYITIDEIQHACKEFGLGDVHLDEVIKEIDQDNDGRIDYGEFAAMMRKGDPGI
GRNRTMRSNLNFNIADAFAEKTTITE

>NISI10G1251

MGCWSSTQKAPPSDFNGYRSTGAPRPTHQTQHHQQQQQQVAQPPKMSVPQNYTTPQA
QKPPPAAAAPPRSAAPATARPAQNPETILGKPFEDIKKHYTLGKELGRGQFGITYLCTEN
STGNTYACKSILKRKLVSKQDKDDMKREIQIMQHLSGQHNIVEFRGAFEDRQSVHLVM
ELCSGGELFDKIIAQGHYTERAAAGICRAIVNVVHSCHFMGVMHRDLKPENFLLSSKDK
NAKLKATDFGLSVFIEERTVYRDIVGSAYYVAPEVLRRSYGKEIDIWSAGVILYILLSGVP
PFWAETEKEIFNEILKGQIDFTSEPWPSISESAKDLVRKMLTQDPRKRITSVQVLEHPWIK

EGGEASDKPIDSAVLSRMKQFRAMNKLKKLALKVIAENLPDEEIKGLKTMFANLDTDNS
GTITYEELKSGLQRLGSRLSEAEVKQLMEAADVDGNGTIDYIEFISATMHRYRLERDEHL
YKAFQYFDKDNSGNITTDELEMAMKEHGIGDETCIKEIIAEVDTDNDGKINYDEFCAMM
RSGTPQPAKLRFD

>NISI03G0127

MGNVCIGSCRTSKDGFFQSISNSFWWSKTSKEVLTHTNKEVISGSKSVNKEAETFTSVQK
SPPEPIKIVKDEVKPPEPTKVKEETKPAQPSSYNNVEFKPSSGQKAEPRRAHSRKPHNVK
RLLSAGLRKESVLQTKTGNLKDYYSLGRKLGHGQFGTTFLCLEKGTGKEYACKSIAKRK
LISDEDVNDVRREIQIMHHLSGHANVVSIKEAYEDSVAVHVVMELCAGGELFDRIIQKG
HYTERKAAELTKTIVGFIESCHALGVMHRDLKPENFLFVSEEEDSPLKAIDFGLSVFFKPG
DIFNDVVGSPYYVAPEVLRKHYGPQADVWSAGVILYILLCGVPPFWGESENEIFNEVLH
GELDFTSDPWPHISESAKDLVRKMLVRDPKKRLTAHEVLCHPWIQIDGVATDKPLDSAV
LTRLKQFSAMNKLKKMALRVIAETLSEEEIAGLKEMFKMIDTDNSGQITFEELKVGLRKF
GSNLEESEIHNLMQAADVDNSGTIDYGEFIAATLHLNKVEREDNLFAAFSYFDKDGSGYI
TQDELQQACEEFGIEDIRLEDMIREVDQDNDGRIDYNEFVAMMQTGNNEFGKKGQQNR
SFSIGFREALPVC

>NISI03G0133

MGNVCIGSCRTSKDGFFQSISNSFWWSKTSKEVLTHTNKEVISGSKSVNKEAETFTSVQK
SPPEPIKIVKDEVKPPEPTKVKEETKPAQPSSYNNVEFKPSSGQKAEPRRAHSRKPHNVK
RLLSAGLRKESVLQTKTGNLKDYYSLGRKLGHGQFGTTFLCLEKGTGKEYACKSIAKRK
LISDEDVNDVRREIQIMHHLSGHANVVSIKEAYEDSVAVHVVMELCAGGELFDRIIQKG
HYTERKAAELTKTIVGFIESCHALGVMHRDLKPENFLFVSEEEDSPLKAIDFGLSVFFKPG
DIFNDVVGSPYYVAPEVLRKHYGPQADVWSAGVILYILLCGVPPFWGESENEIFNEVLH
GELDFTSDPWPHISESAKDLVRKMLVRDPKKRLTAHEVLCHPWIQIDGVATDKPLDSAV
LTRLKQFSAMNKLKKMALRVIAETLSEEEIAGLKEMFKMIDTDNSGQITFEELKVGLRKF
GSNLEESEIHNLMQAADVDNSGTIDYGEFIAATLHLNKVEREDNLFAAFSYFDKDGSGYI
TQDELQQACEEFGIEDIRLEDMIREVDQDNDGRIDYNEFVAMMQTGNNEFGKKGQQNR
SFSIGFREALPVC

>NISI07G2891

MSKFGTGHQRPTRVLPHETPNLTDQYTMGKTLGQGQFGTTYLCTEKTTGRNYACKTIP
KRKLFCQEDYDDVWREIQIMHHLSEHPHVVRIHGTYEDKSCVHIVMELCQGGELFDRIV
KKGHYSEREAAKLIKTIVGVIECCHSLGVMHRDLKPENFLFLSNDEDSSLKATDFGLSVF
YKPGETFSDVVGSPYYVAPEVLCKHYGPESDVWSAGVILYILLSGVPPFWAETETGIFRQ
ILQGKLDLETEPWPSVSDSAKDLIQKMLVRNPKARLSAHDVLCHPWIVDDKVAPDKPL
DSAVLSRLKQFSAMNKLKKMALRVIAERLSEEEIGGLKELFKMIDTDNSGTITFDELKEG
LRRVGSTLMESEIKHLMEAADIDNNGTIDYGEFLAATLHLNKLEREENLVGAFSFFDKD
GSGYITIDELQQACKDFGLDDLHLDEMIKEIDQDNDGQIDYGEFAAMMRKGNGGIGRRT
MRRTINLGEAFGLGALAVTSDNSNQ

>NISI08G1156

MGNTCRGSYRGKFDQGFNQPEDNLSISKPKSQSDHSNSDHSPTTLNSQQPNTPESTKENP
KKDNPLPPPSSKPARKDTIMRRSVDNQTYYVLGHRTPNIRDLYMLGRKLGQGQFGTTYL
CTEISTGNEYACKSISKRKLISKEDVEDVRREIQIMHHLAGHKHIVTIKGAYEDSLYVHIV
MELCGGGELFDRIIQRGHYSERKAAELTRIIVGVVEACHSLGVMHRDLKPENFLLVNKD
DDFSLKAIDFGLSVFFKPGQIFTDVVGSPYYVAPEVLLKHYGPEADVWTAGVILYILLSG
VPPFWAETQQGIFDAVLKGHIDFDSDPWPVISDSAKDLIRKMLCSQPSQRLTAHEVLCHP
WICENGVAPDRALDPAVLSRLKQFSAMNKLKKMALRVIAESLSEEEIAGLREMFTAMD
TDNSGAITFDELKAGLRKYGSTLKDTEIRDLMDAADVDNSGTIDYGEFIAATVHLNKLE
REEHLMAAFQYFDKDGSGYITVDELQLACAEHNMTDVFLEDIIREVDQDNDGRIDYGEF
VAMMQKGNAGIGRRTMRNSLNMSMRGGPNPHRL

>NISI04G0955

MGNCNTCVRPAETAKTDPKNHHHNQQRRKKRSKERKPNPYTVSTAPIHSPAPIRVLKDII
PLSHRTRITDKYILGRELGRGEFGITYLCTDRETKEDLACKSISKRKLRTAVDIEDVRREV
MIMSQLPHHPNIIKLKASYEDSENVHLVMELCGGGELFDRIVARGHYSERAAAAVARTI
MEVVRMCHENGVIHRDLKPENFLFANKKENSPLKAIDFGLSVFFKPGERFSEIVGSPYY
MAPEVLKRNYGPEVDVWSAGVILYILLCGVPPFWAETEQGVALAILRGVLDFKRDPWP
QISDSAKSLVRQMLEPDPKKRLTAQQVLEHPWLQNSKKASNVPLGDIVRTRLKQFSVM
NRFKKRALRVIAEHLSLQEVEVIKDMFTLMDADNDGKVSYEELKAGLRKVGSQLAEPEI
KMLMEVADVDGNGVLDYGEFVAVTIHLQKMENDEHFRRAFMFFDQDGSGYIELDELR
EALADEAGETDVDVLNEILREVDTDKDGRISYEEFAAMMKAGTDWRKASRQYSRERFK
SLSLNLMKDGSLQLHDAATGQAVPV

>NISI03G0956

MGNCCRSPAAVAREDVKSNFSGHDHGRKDAHSSKKQPITVLPGVQKENIEERYLVDRE
LGRGEFGVTYLCIERNTRELLACKSISKRKLRTAVDVDDVRREVAIMKHLPKSSSIVSLK
EACEDDNAVHLVMELCEGGELFDRIVARGHYTERAAAAVTRTIVEVVQLCHKHGVIHR
DLKPENFLFANKKENSPLKAIDFGLSIFFKPGERFSEIVGSPYYMAPEVLKRNYGPEIDIW
SAGVILYILLCGVPPFWAESEQGVAQAILRGQIDFKRDPWPNVSENAKSLVKQMLEPDP
KLRLTAKQVLEHPWLQNAKKAPNVPLGDVVKSRLKQFSMMNRFKRKALKVIADFFSV
EEVEDIKEMFKKIDTDNDGVVSIEELKSGLRNFGSQLAESDVQMLVEAVDTSGKGTLDY
GEFLAVSLHLRRMANDEHLHKAFSYFDKDGNGYIEPDELRESLMEDGADDCTDVANDI
FQEVDTDKDGRISYDEFVAMMKTGTDWRKASRHYSRGRFNSLSIKLMKDGSLNLGNE

>NISI06G1911

MGLCFSRSRSHDIPISSSSDSSPIHRPQPVKRTQQPPSPPPPTLSYSYPVRYSSSSAHIGPILG
KPYVDIKTIYDLDRELGKGQFGITYLCKEKQTGLKYACKSISRRNLVNQKDMDDVRREI
MILQHLTGQPNIVEFKGAYEDKQNLHLVMELCSGGELFDRIIAKGSYSEREAASICRQIV
NVVHVCHFMGVMHRDLKPENFLMVSKDRDAPLKATDFGVSVFIEPGKVYKDIVGSAY
YVAPEVLHRNYGKEIDVWSAGIILYFLLCGVPPFWAENEEGIFEAILEGKLDLQSSPWPSI
STPAKDLIRKMLTREPKKRITAADALAHPWLKDVSETSDKPIDSAVLIRMKQFRAMNKL
KKLALKVIAENLSEEEIKGLKQMFNNMDTDGSGSITFEELRDGLFRLGSKLTEAEIRQLM
DAADVDKNGTIDYSEFITATMHRHKLEKEETLYKAFHYFDKDDSGFITRDEIKQAMNQY

GMGDEATVDEIIEDVDTDEDGRINYEEFVAMMRRGTVKPIANTIPSRVLSNLTFRCYCSG
DSNLDVKEEPITNFAGVKLDDAVDVKAAKLRLDSWISSRVRGISRARVQSSIRSGLVTV
NGQMVNKVSYNVKYGDKISCTISELQPLRAEPEDIPLDIVYEDDHLLVVNKPAHMVVHP
APGNATGTLVNGILHHCSLPTVAYSDDEALSDVDHTSDNESSCLSICAASVRPGIVHRLD
KGTSGLLVVAKDEHSHAHLSKQFKLHTIQRVYVSLSSGVPYPYTGRVEVPIGRDVNNRI
RMAAIPGSSKNGQARYAASRYKVIEILACGGSALVEWRLETGRTHQIRAHAKYVGIPLL
GDELYGGTKNMAMSLLQPRTSPSCHGKLLKLISGIERPCLHALAL

>NISI09G0294

MGNCCASPGSVAEKQTKGRNNKSNPLFGDDYSLTNGSGASKKLWVLKNPTGRDINAQ
YDLGRELGRGEFGITYLCTDANTGERFACKSISKKKLRTAVDIDDVRREVEIMKHLPKHP
NIVTLKDTYEDDSAVHIVMELCEGGELFDRIVARGHYTERAAANVMRTIVEVVQVCHK
HGVMHRDLKPENFLYANKKETSALKTIDFGLSIFFKPGERFNEIVGSPYYMAPEVLKRN
YGPEVDVWSAGVILYILLCGVPPFWAETEQGVAQAIIRSVIDFKRDPWPKVSDNAKDLV
RKMLNPDPKQRLTAQEVLEHPWLLNAKKAPNVPLGETVKARLKQFSVMNKLKKRALR
VIADHLSVEEVAGLKEAFEAMDTGKRGKINLEELKVGLQKFGQQIPDADLQILMEAADV
DGDGTLNYGEFVAVSVHLKKMGNDEHLHKAFAFFDQNRSGYIEVEELRSALNDEDDTN
SEEVINAIMHDVDTDKDGRISYDEFAAMMKAGTDWRKASRQYSRERFNSLSLKLMRDG
SLTTPA

>NISI09G0307

MGNCCASPGSVAEKQTKGRNNKSNPLFGDDYSLTNGSGASKKLWVLKNPTGRDINAQ
YDLGRELGRGEFGITYLCTDANTGERFACKSISKKKLRTAVDIDDVRREVEIMKHLPKHP
NIVTLKDTYEDDSAVHIVMELCEGGELFDRIVARGHYTERAAANVMRTIVEVVQVCHK
HGVMHRDLKPENFLYANKKETSALKTIDFGLSIFFKPGERFNEIVGSPYYMAPEVLKRN
YGPEVDVWSAGVILYILLCGVPPFWAETEQGVAQAIIRSVIDFKRDPWPKVSDNAKDLV
RKMLNPDPKQRLTAQEVLEHPWLLNAKKAPNVPLGETVKARLKQFSVMNKLKKRALR
VIADHLSVEEVAGLKEAFEAMDTGKRGKINLEELKVGLQKFGQQIPDADLQILMEAADV
DGDGTLNYGEFVAVSVHLKKMGNDEHLHKAFAFFDQNRSGYIEVEELRSALNDEDDTN
SEEVINAIMHDVDTDKDGRISYDEFAAMMKAGTDWRKASRQYSRERFNSLSLKLMRDG
SLTTPA

>NISI07G3121

MGVCLSTSKVSGSNSSNNNNRNNANHRRKEVAKPRSPPPKNKDETTRHVHVRYDTKH
RQEQHNQGRNNNQQQQQQLRAKKDKATSRRQTGVIPCGKRTDFGYDKDFDKKYSIGK
LLGHGQFGYTYVAIDKSTGDRVAVKKIDKSKMVLPIAVEDVKREVKILCALAGHENVV
QFYNAFEDDSYVYIAMELCEGGELLDRILAKKDSRYTEKDAAVVVRQMLRVAAECHLH
GLVHRDMKPENFLFKSTKEDSPLKATDFGLSDFIKPGKKFQDIVGSAYYVAPEVLKRKS
GPESDVWSIGVITYILLCGRRPFWDKTEDGIFKEVLRNKPDFRRKPWPTISNSAKDFVKK
LLVKDPRARLTAAQALSHPWVREGGSASDIPIDISVLNNMRQFVKYSRLKQFALRALAS
TLDEEEIADLKDQFAAIDVDKNGSISLEEMRQALAKDLPWKMKDSRVLEILQAIDCNTD
GLVDFTEFVAATLHVHQMEEHDSDKWQKRSQAAFEKFDLDKDGFITPEELRMHTGLKG
SIDPLLEEADIDKDGKISLSEFRRLLRTASISSRNGPSSPATYRRNSRK

>NISI01G0188

MGSCISTGRGSMIFKKTNFRKNHSRRRLNENSKDHLSSKNTTNSNIACVLPNPNEGNIHE
KYKLGRELGRGEFGITHQCFHVDSGEEYACKIIAKQNLRTEIDREDVKREVEIMRRLPKH
HNIVTFKEAYEDKEAIYLVMELCGGGELFDRIVARGHYTERAAAKVTKTILEIVKVCHE
NGVIHRDLKPENFLFADASENSQLKAIDFGLSITFSPGQRFSEIVGSPYYMAPEVLRRDYG
AEVDVWSTGVILYILLCGVPPFWAETEEGIAHAIVKGHIDFERDPWPKVSDEAKKLVKS
MLDPNPYSRLTVKEVLEHPWIQNENHAPNISLGENVRTKIKQFSLMNKFKKKVLRVVA
DHLPTDQVAGIKQMFYMMDTNKTGELTFEELKDGLQMIGHKVSDPDVQMLMDAADID
ENGTLDCEEFVTMSVHLLKKIGNDENLSQAFGYFDKNQTGFIEFDELREALVDENGHNS
EQMIQDIIFDVDLDKDGRICYEEFKAMMQKGMDWKMSSRQYSRAMLNALSIKLFKDKS
MPLSDKSMPLS

>NISI03G2595

MGNCCVTLKPGDHEGKKKHKKKKQNPFSLDYGNHQNGHKLCVLKEPTGSEIGERYEL
GRELGRGEFGVTYLCTDKSSRDTFACKSISKKKLRTAVDIEDVRREVEIMRNLPKHPNIV
TLKDTYEDDNAVHLVMELCEGGELFDRIVARGHYTERAAAAVTKTIVEVVQMCHKNG
VMHRDLKPENFLFANKKETAPLKAIDFGLSVFFKPGERFSEIVGSPYYMAPEVLKRNYG
PEVDVWSAGVILYILLCGVPPFWAETEQGVAQAIIRSVIDFKRDPWPKVSDNAKDLVRK
MLDPDPQRRLTAQQVLEHPWLQNAKKAPNVSLGETVRARLKQFSVMNKLKKRALRVI
AEHLSVEEVAGIKEGFEMMDIAKRGKINIDELRSGLHKLGHQIPDTDLQILMEAGDVDR
DGYLDYGEFVAISVHLRKMGNDEHLRKAFQFFDQNQTGYIEMEELADALSDEIDTSEEV
INAIMHDVDTDKDGRISYEEFAAMMKAGTDWRKASRQYSRERFNSLSLKLMRDGSLQQ
LNKMEVR

Supplementary Data S2. The promoter sequences（3 kb）of the *NsCDPKs* isolated from *N. Sibirica* genome

>NISI01G0188

AATCTCCAGGCTGCAATAACGGAGTTCTTTTGTTGGCTCAAGATTCCGATTGGATTC
CTTGGTCGGTTACGCAAAGAAGATCGTCTGCGGCGGTGGCGCCGCCCTGCTTGGTTG
ATTGTGACGGAGGACTTTGAGATAGAATAAACTTTTTTTTTTTTTTTTTTGAAAACC
GACGTCGGAAATCTACTCGCATCTGTGAAACAAAATTTACAGGACGACCCTAGAGTT
TTATTCTAATAAAATGTAGTTCAGAGGCTCCAATTGTATAGTTATAACTTATTTCTCA
ATTGTATAGTTATAACTTATAATGTCAATGAATTATTTTACCATATTTTCTTTATTATT
CACAGGTGTTTAGATCAATTCCCGTTTATTTCGCAAATATATATGTCCACAACACAA
CTGACTTTTGCATCTTTGGTCTGACTAAGGTTTGAGTTCTTCGATCATTGTAATATGT
AAGGGCCGAATTATCTGTGTAGGACGTCACATTTCCCCTCAGTAACTTTTCAAAAAA
AAAATAATAATAATAAAAAATCTCATATATCAACTAATCTCTATTTGCAGTGCATGA
CTGTCGTTGGCCAATAATTATTTTGAAAACGTTAATAAGTAAACTAGAAGTTACTTA
ATCGACTCAAAGTCTACTCCAGTGATAACCTTATATCATTACGCCAGAAGGCTTGCT
TATTGTTTTGTCACTTTTAGTTTTGATCCAAAGTACTTAAATTTTGTTTCATGTTAATA
ATACAAAAAATAAAGGTAAATTATAACTTTGATTTGTCATATTTGAAGTATTGTTAA
CTTGATCACTTCTTTTTCAAACGCTAACAGTTTGGACACTACTTTTTTGAATAATTAA
ACTTCAGTCATACTTTTGTCATGTCATCATCGCCAATTATCTTAATACTACAATAATA
CTACGGTTGGATTTGTTTTGAACTTACGAGTCTAACCATATAACTTTATACCGGAAA
CGATGCCAAAATATGATCAAAATGGCCAAAAATCGTCTGGCGCCTACATGAGTGTG
GGACACAACCACCAAAGGTGATTCAAATATCGTTGCTAATATGTTAAGGATCCCACA
TTGAAAAGATAAAGGGACCTTACCATATTAAGATTCTATGGGGTTCCATCACAAAAC
CAATTGGCAATGTGAGGAGTAGCCCATAGACCTTATAAACCTTGCAAGGTCCCTTTA
TCTTTTCAATGTGGGATCCTTAACATGCCCCCTCAAGATGGTGGCTCTTTTTTTCTTTT
TAGCTCACCAATCTTGAGCCGGATCCCATGGATCTCAGATTCACCATTTTTGGGTCG
GATGCTCGTTTGAGCTTAGTGGGCTCTGATACCATATTAAGATTCTATGGGGTTCCAT
CACAAAACCAATTGGCAATGTGAGGAGTAGCCCATAGACCTTATAAACCTTGCAAG
GTCCCTTTATCTTTTCAATGTGGGATCCTTAACATAATACCAGTAGATTCGTCGAAGT
TCATTGAGAATCATGATAGTTTCATTTTTTGCGTTTAGTGAGCCAAATCATATATATC
TAAGTTTAAACTATATACACACAAACACACACATATAAGTATGTATGTGTATATGTA
TGTAATTATATAAATACTTTTAAATGTTATAATTACTGAAATAAAAAATATTAAAAA
TTAGACAAAATCTTAAGTACATGTATGTATTAGTGCATGCATTATCCTTTAATAAAA
TAATATGTATATTCATCCACATGGTATTGGATGGGGTAGTTTTTAGTGAGTATAATAT
GCTTTATCATACATTAGAGCTAAGTTTTATTAAATATAATATTTACAAAAAAAAAATG
ACAAATAAGGGAGATTAATAAAAAGTGAGTAAATAAATAAATAAAGAAGAGAGCA
ATCCACCGTTGAGGTCTCCTTAAACCTCTCCTACCATGTCGGAGGGGACCTTTACGG
CAATGGAGGAGATAAGATCAGGTCGAAGGAGGCTGAGCTTCGGTATCCTCCGACAC
AGTCTGTATTTTCCTCTAAATTCAATATCTTCTGACTTGGAAGAATCTGAATCATATG
TCATTTGACACTATAGACGTCTTTTTTTGTGTCAAATCAGACCAATCTGGTTGCCTCC
ATCATCAAAGAGACCAAATCTCTCCTCCCTGACGTGAAGGAGACCGTTTGCCTCTAC
TAGTCATCCCCGTATGTAATACATGTTCTCCGACACGATAACTCACGGTGGAGACCT

CCACCGTGAGTTTAGATCTATTTTTTTTATAATTACTTTAATAGTTTATTTGATTTTCA
ATAATTAAAGTGTGAGGTAATTATTAATGTTATTTGTTTTGAGATTAGAGGATAAGT
AGTTACTGATTTTATTTTTATTTTCTTACTTGTACTCAAGTGATTGATTCCATTAGATC
AGTCTTAAAATAAAATAAAATAAAAAAATCTAAAGGTTAAAATTTTTTATTTGTATT
CCTATATGCATGTAGTGTAGACATACTCTAAAAATTATATTTAAATATTTATACACAT
TAAAAACACGTATTCAAAGCCTAATAAAATAAAAACTTCGGTGCATCCAGCATTATT
ATCATAAATAAATTAAATGTATTTGACTTTCCTTTATAGATTATTTTACAAGTTATCA
ACTGAACTACATTGGAGAATTTGTCCTAAAAAGTTGGCAAGACAGGGCAAACATGT
AATAAAAATATAACAAAATTTCCAACTTATAAACTTCCCAATTGCCAAATTTAGCGT
CACTTTTTCCTCTCATCTTTCGAAATTCTATTTCGGTATTAGGGTTTCCATTTTCTTTT
TGGGGAAAAAGAGAAGGGAAAAAGAAAAAGAAATCGGTTCGCGATCTCTTTCGCA
GAACTCGATTTTTCATTTACTTTTTCAGCTTCCCATGAGATGTTAATCTACGAAATAA
GATCATACTGAAGAAATTAAAAGGAAAAAAAAAATAAAAAAAAATCAAAGGATAT
TAGAGTTTCGGAAACATCTC

>NISI02G1128

GGAGGTATCAAAGTGTAATTAACCCCTATTTCGAGATACCATAATGTAGAGTAACTT
TCGAGAAGGTACTAAAATGTAATTATATCCTATTTTGAGGTACCAAAGTGTAATTTG
GCCATATTGATAATTTAAATATTACAACCAAGAGGTGTATAAAATTGAAATTACGAA
CGCGTTTGGACGCACACCCACCCAGTAACAATCAATCAAGACATCCATGTGGCGAC
GTGTCAATGAATTTTCATGTGGGACCCACAGGACATGTCTGAAAAGTTGAATTTGGT
CCCAGGAGCTTCACAGCATCAGCCTACTCTTTTGATTAATCTTTCAATCCAATGACA
AATATGAAAGCCCCAATGAACATACTTTTGTGGACTCTATCAATGCCGGAATTAAAC
ATACATAAATGATGGCATACATACACATGTGCCTACATACATACATACATACATTAC
TACATGCATGCATGGCTTTATGTATAAAAAAAAAACATCCCTTACTTGTCTATCTACA
AAAACGAATTTAATAAGTTATTTAAACAGAATAAACTTACAAAATTAAAAATAAAA
AAATTCATTTATGTTTCTATACAGATTGTTATAATATCAAATTTAATTAGAAGATTTA
ATAATATCATCTATGTCCTTATTGTAAAGTAGAAGTTTGTTAATAAAAACAGATATA
TTCCGCAGTAATTTTTTTATCACTACTGGACTCATCAATATTAGAAAAATTGTTACCT
TTTGAGAAGATGGAGAGTTTCCTGACTTGCAACATATATCCAAAACTAGATATAATC
TTGTTTTTTTTTTTTTTTTTTTTCTTTTTTTTTCTAAGTTTTAATTTTATTCAATGGTAA
AATGATTATCATTTATATTTTGTTTCACACGTTAGTAAAAATTAATAAAAAATACTA
ACAGTCGAGTCATAAATGATATTATTAAATCTTCTAACTATATTTGATAGTATAACA
ATTCGTAAGGATGTTAATAAAATTATCCCTTCAAAAATTCAAATAAAAAATCCCTTT
ATGTTTTTATGGGTAGATTATATAATAAATCATATTAAATTGAGTTATATATATATAT
ATAATTTTTTTTGTTGGTATTTTTAGGAATTACATTATTGTATTAAATAATGCTAACT
CTTATTTTTCAAAATCACAGAAATGAAAATTGAACTTTGGCTAATAGCAGGGACTAT
GTTCCATACCCTTGCTTACCTGCAGCAATACAAGAAGGAAAAAGGAGTCAGAGAAC
AAGTTAGCAAAAGAAAACAAACTCCGAAAAAGGAACTTCCTTTCACCTCTTCCAGA
TCTAAGAACCTAAAGCAAGCACACGTTCTCGATTTATTTTTATATGATGGATTGGAG
AAGATGTAAAATAAAAATAAAATTTATTAATGATAATTTATGAAAATATAAAGACA
AAGAGCATCGATTAATCTCATGCTACTATATACCTTGATTATTTTATATGATATATTA
AAATCAAACATATAAATGATGATTCATATCACTCGGATTTTTGACACGAAGTATCTA

AAAATATCAAATCACAATATTTAAGATTATCATCCATATGGGAGACAATAATTTGGG
ATAAGATTAGTTGACTTAGAAGGAGTATTATTGTTTTTATTAATATTACTTATCTCAG
ACTGTGAAAGGTTTTTTTTTTTTTTTTTTTTTTTTTTTTTTTTTTTTAAGAAAAGAATAAAAA
TAAACTCTTATTACTGGAAGTATGTTGAGTCTGAAATGTTTTAAACAAAAAGTTATC
GGTTTGAAAATATAAAATTATTAAGAAAAATTTACTGTTATTTTTTGTGTTTTTTGTA
AAGACTAGGCTTATACAGATACGAAAATACGCCGAATGTATAGAAAAAAAGAAAAT
AAGTATCGATGATGTATGCAAATATAATGAAACAAGATATTATCCAATTTACATGTT
CAACTTCCAAAAGTTCAGCTATTTTTCATTACATAAAGCAATTAACAAAACCAAAAA
GTTTAAACAAGGACTGGTTTGAAAATTCGATTTTAAATATTACATCTAAATATCAAA
TTACTTTTGACAAAAATATTTTTAGTAAAAGTACAACACTCTAAATCGTCAATCAAA
GTTATCTCTCGGTAAATTATAAAAAAAAATAATATTATTCATTCTATTTTGTATTATAT
TTTTTTATCTCATATTTGATATGTTAAAACATGATCGATAGTTTTATATACATTAATT
ATATTTTGATACATCAAATATAAAATAAAAAAAATATAATATAAGATAAAGTAAATA
ATATTATATTTATAAAAAAAATGGATAGTGACACGTAAAGGAGAACACCTGCGATG
CGAGAATGCAAATGCATATTCAACTTGACGCGCTTTAATTAATCGGATTTAAAATAT
GAGGAAACCAATTAGATGGACACGTGTTTTTTTTTTTTGAACTTGACACGTGTTGAA
ATAGAAAGGGTATATACCAAATACCAGAATTTTATATTACTAGTTAGAATCAATAGT
GATTTGCTTTTGTCCGTATAGTGGATATTTTCCATTCGTAATTGCATTAATTAAATAT
TAAATTATCTTCTAATAATCTTCAAAAAAATGGAAACTATAAAAAAATGAAAAAAAATG
AAAAAAATAAAAAATAAAAATGGAAAATCTGTTGACAGCTTTGTTTCATTACCTACT
CCACTGCTGTGTTTCAGCTACAAAGAATTCTCTTTCGTTCTCTCTGGTTCAAAGGTTG
ACCAAATAATTTTCAATTTTTTTCTTTCTATATGGCTATTCAGTATCAAACTTTTCCGG
GAAAATTTCATCCTTGAAAAGCCAAAATCTTTGATCATCTTTCCCAAAACTCTTCTAT
ATAATATTATTGCTTCTTCTCTCGATTCCTTCATCTGGGTTTCATATTTTTCCATCGTT
TTTACTTCCCTTTTTCTGAAATTCTCACAAACCCCACTTCAATCACTGGATGTTTTGA
TCCAATTTGCAGTCTTTCAGAG

>NISI02G1090

GTATTTCAAAAATTGGAGGTATCAAAGTGTAATTAACCCCTATTTCGAGGTACCATA
ATGTAGAGTAACTTTCGAGGAGGTACTAAAATGTAATTATATCCTATTTCGAGGTAC
CAAAGTGTAATTTGACCATATTGATAATTTAAATATTACAACCAAGAGGTGTATAAA
ATTGAAATTACGAACGCGTTTGGACGCACACCCACCCAGTAACAATCAATCAAGAC
ATCCATGTGGCGACGTGTCAATGAATTTTCATGTGGGACCCACAGGACATGTCTGAA
AAGTTGAATTTGGTCCCAGGAGCTTCACAGCATCAGCCTACTCTTTTGATTAATCTTT
CAATCCAATGACAAATATGAAAGCCCCAATGAACATACTTTTGTGGACTCTATCAAT
GCCGGAATTAAACATACATAAATGATGGCATACATACACATGTGCCTACATACATAC
ATACATACATTACTACATGCATGCATGGCTTTATGTATAAAAAAAACATCCCTTACT
TGTCTATCTACAAAAACGAATTTAATAAGTTATTTAAACAGAATAAACTTACAAAAT
TAAAAATAAAAAAATTCATTTATGCTTCTATACAGATTGTTATAATATCAAATTTAA
TTAGAAGATTTAATAATATCATCTATGTCCTTATTGTAAGTAGAAGTTTGTTAATAAA
AACAGATATATTCCGCAGTAATTTTTTTATCACTACTGGACTCATCAATATTAGAAA
AATTGTTACCTTTTGAGAAGATGGAGAGTTTCCTGACTTGCAACATATATCCAAAAC
TAGATATAATCTTGTTTTTTTTTTTTTTTTTTTTTTTTTTTTTTTTTTTCTAAGTTTAATTTTAT

TCAATGGTAAAATGATTATCATTTATATTTTGTTTCACACGTTAGTAAAAATTAATAA
AAAATACTAACAGTCGAGTCATAAATGATATTATTAAATCTTCTAACTATATTTGAT
AGTATAACAATTCGTAAGGATGTTAATAAAATTATCCCTTCAAAAATTCAAATAAAA
AATCCCTTTATGTTTTTATGGGTAGATTATATAATAAATCATATTAAATTGAGTTATA
TATATATATAATTTTTTTTGTTGGTATTTTTAGGAATTACATTATTATATTAAATAATG
CTAACTCTTATTTTTCAAAATCACAGAAATGAAAATTGAACTTTGGCTAATAGCAGG
GACTATGTTCCATACCCTTGCTTACCTGCAGCAATACAAGAAGGAAAAAGGAGTCA
GAGAACAAGTTAGCAAAAGAAAACAAACTCCGAAAAAGGAACTTTCTTTCACCTCT
TCCAGATCTAAGAACCTAAAGCAAGCACACGTTCTCGATTTATTTTTATATGATGGA
TTGGAGAAGATGTAAAATAAAAATAAAATTTATTAATGATAATTTATGAAAATATA
AAGACAAAGAGCATCGATTAATCTCATGCTACTATATACCTTGATTATTTTATATGA
TATATTAAAATCAAACATATAAATGATGATTCATATCACTCGGATTTTTGACACGAA
GTATCTAAAAATATCAAATCACAATATTTAAGATTATCATCCATATGGGAGACAATA
ATTTGGGATAAGATTAGTTGACTTAGAAGGAGTATTATTGTTTTTATTAATATTACTT
ATCTCAGACTGTTTTTTTTTTTTTTTTTTTTTTTTTTTAAGAAAAGAATAAAAATAAAC
TCTTATTACTGGAAGTATGTTGAGTCTGAAATGTTTTAAACAAAAAAGTTATCGGTT
TGAAAATATAAAATTATTAAGAAAAATTTACGGTTATTTTTTGTGTTTTTTGTAAAGA
CTAGGCTTATACAGATACGAAAATACGCCGAATGTATAGAAAAAAAAGAAAATAAGT
ATCGATGATGTATGCAAATATAATGAAACAAGATATTATCCAATTTACATGTTCAAC
TTCCAAAAGTTCAGCTATTTTTCATTACATAAAGCAATTAACAAAACCAAAAAGTTT
AAACAAGGACTGGTTTGAAAATTCGATTTTAAATATTACATCTAAATATCAAATTAC
TTTCGACAAAAATATTTTTAGTAAAAGTACAACACTCTAAATCGTCAATCAAAGTTA
TCTCTCGGTAAATTATAAAAAAAAATAATATTATTCATTCTATTTTGTATTATATTTTTT
TATCTCATATTTGATATGTTAAAATATGATCGATAGTTTTATATACATTAATTATATT
TTGATACATCAAATATAAAATAAAAAAATATAATATAAGATAAGGTAAATAATATT
ATATTTATAAAAAAAATCGATAGTGACACGTAAAGGAGTTCCTGCGATGCGAGAAT
GCAAATGCATATTCAACTTGACGCGCTTTAATTAATCGGATTTAAAATATGAGGAAA
CCAATTAGATGGACACGTGTTTTTTTTTTTTTGTTGAACTTGACACGTGTTGAAATAGA
AAGGGTATATACCAAATACCAGAATTTTATATTACTAGTTAGAATCAATAGTGATTT
GCTTTTGTCCGTATAGTGGATATTTTTCCATTCGTAATTGCATTAATTAAATATTAAA
TTATCTTCTAATAATCTTCAAAAAATGGAAACTATAAAAAATGAAAAAAATGAAAA
AAATAAAAAATAAAAATGGAAAATCTGTTGACAGCTTTGTTTCATTACCTACTCCAC
TGCTGTGTTTCAGCTACAAAGAATTCTCTTTCGTTCTCTCTGGTTCAAAGGTTGACCA
AATAATTTTCAATTTTTTTCTTTCTATATGGCTATTCAGTATCAAACTTTTCCGGGAA
AATTTCATCCTTGAAAAGCCAAAATCTTTGATCATCTTTCCCAAAACTCTTCTATATA
ATATTATTGCTTCTTCTCTCGATTCCTTCATCTGGGTTTCATATTTTTCCATCGTTTTT
ACTTCCCTTTTTCTGAAATTCTCACAAACCCCACTTCAATCACTGGATGTTTTGATCC
AATTTGCAGTCTTTCAGAG

>NISI03G0127

CCATATATTATTCTAACACCTGCCACTATACAAATTTAAAATTAAGAAGAAAAAAAT
ATATTTCTTTCTTATTATCTATTGTGACATAATCTACTATACATTTACATCTATACGG
AGTGTGTTTAACCGTTGGATTGTAGGGAAAAGATTCAATGGTCGACACATTAATAAA

ATAATCATTTACAGGGATTAATCTCAAGGGAAAAAAAAAAAAAAAAAATAGAGATTTA
CCATGATTTGAATCCTTTGAGTGGGTAGTATTACAAGAGAAGCAAACGTCAAACAA
AAGAAAGGGAAAATTTTACATGGTTCTTTTAGGTTTTATCTAAATTTTATATAGTCCT
ACAAATCTTAATAAACCTTTAGCAAACGTCAAACAAAAGAAAGGGAAAATTTTACA
TGGTTCTTTTAGGTTTTATCTAAATTTTATATAGTCCTACAAATCTTAATAAACCTTT
AGCAAACGTCAAACAAAAGAAAGGGAAAATTTTACATGGTTCTTTTAGGTTTTATCT
AAATTTTATATAGTCCTACAAATCTTAATAAACCTTTAGCAAACGTCAAACAAAAGA
AAGGGAAAATTTTACATGGTTCTTTTAGGTTTTATCTAAATTTTATATAGTCCTATAA
ATCTTAATAAACCTTTACTTGGCCCCTCTGCCTTCAATTCAAGTAACCATGATTAAAG
ATTAATCCTATAAGAATGTCCAAACATATATAGGAATTAGTTTGGACAACTGATAAG
AACAGTTTTGGGATACAAGAATATTCAAGGACAATATATATGCCAACAAAAAACAA
AAATTCTAAATAGGGGTAGACACAAATCATATTTTGTGAAATTTACGTGAATCCAAA
TCCAATTCTTATAATGGATTAAAGAAATCTTGATCCGTACCAATCCACTCAAGACAT
TGGATCGGATCAGATTGATATTTTACCAGATTGGACTGAATTCTTTCCATATATCGAT
ATATGTGTATCTAACAAACCAATGTGATAATTTCAACTAGTTTTTATGTTTGTCAAAA
CCCCAGTCTCATATGTTTTAGAAATGTTCACAAAGTCACTTTTTCGTGAAAATATTTC
GTTAATGTTCACCTAATAGGAATAGGAGAAATTTTTCTCTTTAAAAAAAAAAAAAAA
AAAAAAAAAAAAAAAAAGAGAGAGAGAGAGAGAGAGAGAGAGAAAAGGAAGAACA
AGAGGAAAGCTATGAATCAGATAATGCTTAAAATAATATTTCATTTATCCACAAGAT
TCTTATAATAGTAAACTAATTAGAAAAACAACTTATTTAATCCCTGACTTTGTCAAG
ATAAAATCTGTTAATACAAACAAATATATCATTAAAATGATTTTTTACAATGGTTGG
ACTCGTCGATGGCTGCATAAATTTTGTCTTGAGGGAGGAAGGGAGCTCTTTTGCTCA
TGGCAGATTTACAAAACAAAGTCGAATATTTTTTTTTTTTTTATTGGATTTAAGAGTA
AGACGACGATCCTTATAATATTTTTTCCATATTAGTAATCATTAATTAAAAAATATAT
ATATATTGTAAACGGTAATATTACATGTAATATTTAATAATCTTCTAATTATATTTTA
TGTTATGACAATATATGGAGTTATAAATAAAATTATCTCAGACTAAAAGAAAGAAA
CAACATCCTTACCCAAAAAAAAAATTAAAAAATAAAAACAACATATTAAATTTTGT
AATTTAGGGAAAAAATTGAGAATCATAAATAACTATAAAATACCTATTTAATAATCT
AATTTTAATTTATTTTATTTTAAAGAGTTAGATGTATGAACAATAATCGGACTGTTGA
AAAACATTCAGTTTTACATGAAGAAAACCCTCAATTTTTGTTTGTTGTTTAATTTTTG
TTCTTGTTTTACAAATATATGACTGTTTATAAATTAGAACCGGCCTAGGACGGCAGC
GGACTTAAGTAGTAAGCCGGCGCAATCAAGTAAAACAGAAGATCACGAGAAGATCA
TGGCCGTCGATCTCCACTGGCCCAAATCAAGCACTTTAATCGGACGGTATAAATTCA
TCCAAAGCAAAAACGACTTATACCGTTTTTACACATACGCGTTTTCCTTTTTATTATA
TTTGTTTAAAAGAAAAATATATATATATATTTTTTTTATTTTAAATATTTGAAATCTTT
TCTCTAACAGAAAAACCTGTCTCTCCAATACTCTTTGGTTTTTCCCTCAAATTCCTTT
CCCTTTTCGTGCGCGTAGCGAAATGGGGGAGAAATTCCGAATCTTTCCAAAACCTAA
AAGATTTTACAATGAAATCAAAACCCTAATCAGTTTCGTATAATATAAAGACAAAAT
CCTCCTCGTTCGTTCATTTACATTCTCTCACGCAGACCTGGTAAACTCAAAACCACAT
TGTGTTTATTCTACTAACTTGAGACTTCTTCTCTTTTTGTCGATTTTCCCTCTGTTTCCT
GGAAATTTACATTGTTTCTTTCGTGGGGTCATTATCTCAATAAATTTTTATTTTAGGC
TCTAAAAGTCAATTGTTTATCGTTGTTCTCGGCAACTTGCGGCTTGTTTATGGTTCAG
CGAAGGTTCCAATTCTGGGAATATTTATTGGAATAAGAGTATCCAGGGTTTGACGAG

CTTGTTGGATAATTGCTTTTGTGAAATAGGGAGTTTAGCCATGTACGTTGACATTTGA
TAATATCTGAATCATTCTCTTGTTGAGCATCCATCATCGTTAATGCTGTGTCTGGATG
CTTTTATCATTGAATTCTCAAACTTTGCTTGTTATAAATGTTCATATTGAAAAGTAAG
TCCCGTCATAGTGTGGATATATTTATAAGTTTGAGTTATATGCTGCTGCAAGGATCC
ATCACGATATTGACAACTTAAATTTGGGGGGAGGGGAAATGTTTGTGGTTTGTGTGT
ATTATATAAAACCATTTAAGAGACAATACTAGAGAGAAGTGTCCATAATAGATTAT
ATTTAGGGTTTGTAGTTTTAG

>NISI03G0133

AAATAAAATATCACTATGTATATTATATCATATACCCCATAACGATAGAATCCAAGA
AAATCATATAAAATATTCTAAAAATTAATAGAAAATATTATTCCTTTCATATTTCTAT
GTCTAGAGTTAAAGTTTGATTCTAATTAGAACCATAAAACATGTAACATTATCAACT
AGCGACTTTTCTACAAATAGATTATGACAATAATTAGATTATTAATAATAATCTTAC
CTAACATGCACTATTTCCATATATTATTCTAACACCTGCCACTATACAAATTTAAAAT
TAAGAAGAAAAAAAAATATTTCTTTCTTATTATCTATTGTGACATAATCTACTATACAT
TTACATCTATACGGAGTGTGTTTAACCGTTGGATTGTAGGGAAAAGATTCAATGGTC
GACACATTAATAAAATAATCATTTACAGGGATTAATCTCAAGGGAAAAAAAAAAAA
AAAAAAAAAAAAAAAAAAAAGAGATTTACCATGATTTGAATCCTTTGAGTGGGTAG
TATTACAAGAGAAGCAAACGTCAAACAAAAGAAAGGGAAAATTTTACATGGTTCTT
TTAGGTTTTATCTAAATTTTATATAGTCCTATAAATCTTAATAAACCTTTACTTAGCC
CCTCTGCCTTCAATTCAAGTAACCATGATTAAAGATTAATCCTATAAGAATGTCCAA
ACATATATAGGAATTAGTTTGGACAACTGATAAGAACAGTTTTGGGATACAAGAAT
ATTCAAGGACAATATATATGCCAACGAAAAACAAAAATTCTAAATAGGGGTAGACA
CAAATCATATTTTGTGAAATTTACATGAATCCAAATCCAATTCTTATAATGGATCAA
AGAAATCTTGATCCGTACCAATCCCTCAAGACATTGGATCGGATCAGATTGATATTT
TACCAGATTGGACTGAATTCTTTCCATATATCGATATATGTGTATCTAACAAGCCAA
TGTGATAATTTCAGCTAGTTTTTATGTTTGTCAAAACCCCAGTCTCATATGTTTTAGA
AATGTTCACAAAGTCACTTTTTCGTGAAAATATTTCGTCAATGTTCACCTAATAGGA
ATAGGAGAAATTTTTCTCTTTAAAAAAAAAAAAAAAAAAAAGAGAGAGAGAGAGAG
AAAAGGAAGAACGAGAGGAAAGCTATGAATCAGATAATGCTTAAAATAATATTTCA
TTTATCCACAAGATTCTTATAATAGTAAACTAATTAGAAAAACAACTTATTTAATCC
CTGACTTTGTCAAGATAAAATCTGTTAATACAAACAAATATATCATTAAAATGATTT
TTTACAATGGTTGGACTCGTCGATGGCTGCATAAATTTTGTCTTGAGGGAGGAAGGG
AGCTCTTTTGCTCATGGCAGATTTACAAAACAAAGTTGAATATTTTTTTTTTTTTATTT
ATTTATTGGATTTAAGAGTAAGACGACGATCCTTATAATATTTTTTCCATATTAGTAA
TCATTAATTAAAAATATATATATATATATATATATATATATATATATATATATATATA
TATATATTGTAAACGGTAAGATTACATGTAATATTTAATAATCTTCTAATTATATTTT
ATATTATGACAATATATGGAGTTATAAATAAAATTATCTCAGACTAAAAGAAAGAA
ACAACATCCTTACCCAAAAAAAAAAAAAAATTAAAAAATAAAAACAACATATTAAAT
TTTGTAATTTAGGGAAAAAATTGAGAATCATAAATAACTATAAAATACCTATTTAAT
AATCTAATTTTAATTTATTTTATTTTAAAGAGTTAGATGTATGAACAATAATCGGACT
GTTGAAAAACATTCAGTTTTACATGAAGAAAACCCTCAATTTTTGTTTGTTGTTTAAT
TTTTGTTCTTGTTTTACAAATATATGACTGTTTATAAATTAGAACCGGCCTAGGACGG

CAGCGGACTTAAGTAGTAAGCCGGCGCATTCAAGTAAAACAGAAGATCACGAGAAG
ATCATGGCCGTCGATCTCTACTGGCCTAAATCAAGCACTTTAATCGGACGGTATAAA
TTCATCCAAAGCAAAAACGACTTATACCGTTTTTACACATACGCGTTTTCCTTTTTAT
TATATTTGTTTAAAAGAAAAATATATTTTTTTTTTTTTATTTTAAATATTTGAAATCTT
TTCTCTAACAGAAAAACCTGTCTCTCCAATACTCTTTGGTTTTTCCCTCAAATTCCTTT
CCCTTTTCGTGCGCGTAGCGAAATGGGGGAGAAATTCCGAATCTTTCCAAAACCTAA
AAGATTTTACAATGAAATCAAAACCCTAATCAGTTTCGTATAATATAAAGACAAAAT
CCTCCTCGTTCGTTCATTTACATTCTCTCACGCAGACGTGGTAAACTCAAAACCACAT
TGTGTTTATTCTACTAACTTGAGACTTCTTCTCTTTTTGTCGATTTTCCCTCTGTTTCCT
GGAAATTTACATTGTTTCTTTCGTGGGGTCATTATCTCAATAAATTTTTATTTTAGGC
TCTAAAAGTCAATTGTTTATCGTTGTTCTCGGCAACTTGCGGCTTGTTTATGGTTCAG
CGAAGGTTCCAATTCTGGGAATATTTATTGGAATAAGAGTATCCAGGGTTTGACGAG
CTTGTTGGATAATTGCTTTTGTGAAATAGGGAGTTTAGCCATGTACGTTGACATTTGA
TAATATCTGAATCATTCTCTTGTTGAGCATCCATCATCGTTAATGCTGTGTCTGGATG
CTTTTATCATTGAATTCTCAAACTTCGCTTGTTATAAATGTTCATATTGAAAAGTAAG
TCCCGTCATAGTGTGGATATATTTATAAGTTTGAGTTATATGCTGCTGCAAGGATCC
ATCACGATGTTGACAACTTAAATTTGGGGGGAGGGGAAATGTTTGTGGTTTGTGTGT
ATTATATAAAACCATTTAAGAGACAATACTAGAGAGAAGTGTGCATAATAGATTAT
ATTTAGGGTTTGTAGTTTTAG

>NISI03G2595

AGACGTCTTCGCTATGTAATCATAAGTCATTATTTATCATCTGTTTCAAACGGTCATG
ACAAAAAAAAAAAAAAAAAAAAAAAAAAAAAAAAAGAAAAAGTCTGTGACAAATA
AAGATTGATTTTATTTCTTTTACATACTGAGAATGATGATTATAGATTTATAGTAAAT
ATTCAGGTAAAAAAAAAAAAAAAAAATTACAGCGTCGGTCCAACTAAAATCCTTTTC
TTCCCTTTAAAGATCAAAGATGATTGATTTATTAATTATTATTGTTATTATTTTCCAC
AGCTATAATTGTTTTTTTTTTTTTTAATTTTATTTATTTTAAGACAAATTTACCATCCC
AAATAAAATATCAAAAAATAGTATCTTAAATGATATGAAAGTAATTAATCAATTTTT
TGAATTTAATTTTATTTGACCAATTACTTTTGATTATATCATTTGAAATTTTACTTTTT
TAAAATTTCATTTGAGATGTGTGTTTTTCTTTTTTTTTTAGTCAACAAAGTAAATAAA
GATTGGGTCATGTTTGCTTGCCTTTCTTGCATATGCCTCATCAATTTGTTATCACAAC
TTATCAAATTTTTGCCCATCAAATTTCGTCCACTTTGCCTTGCTAGCCTTTTGTCCTTT
ATGGGGTTTTTATAGATTTAGACAAAATATAGTGTGATTCAGATGATTACTCATACA
AAAATAAAATGTTTATATAATTTGAGAGGAATATTATTATTATTCTAAGAAAAGTTT
ATCCCAAAGTTAATGTTTTAAAGTTTATTTTACTGCGAGGTGAATCGATCATTTATCC
TCCTTACCTATACTTGTTCAATTATCGTTTTATATAAAAATCAAATGCTATGAATTAT
TAATAAGGCCAATTATAACTTTGGTCACTCGAGTTTGACAAAATGTCAGTTTGGTCA
CTCTTTTTTTTTTTCAGATTTTAACTTTGGTCACTCGAAATTTAAAAAATATAACTTTG
GTCACTGTCAACGAAAAGTCAACGTTAATTTTATTGAAAAGACCAAAATACCCTTCT
ACTAATATTATTATTACATTTTATATTTTAAATTAAAATAAAAAATCAATAAAATAA
TAATTTTAAAGAATTAAAAAAATATAAATAATATCAAAACTCACCTCCACCGCCGCC
GGCCCCCGACCACCACCGTCTCCACCATCCGGTCTCCGTTTTTCAATAAAAAGGTAT
GGTTGGACTCTTTACGACCTCACGAGTCCAACCGTACCTTTTTTTTTTGAAAAATACA

CCGAATCCGGCCGAAACGGCCGGGAAAAGGTCCGGCCTCGCCGGCGCGTGGGCCCC
ACGCGCCACCTCCGGCGTCGATTCGTCCGGCGCCGTTGGTACTACTAAACTCGAAAT
GTCTCACTGAGTCTATGGTGGTCTTATTTTTCCCATTTGAGGTCCTGTAGCTCGTAGA
TCTGAGTTTTATTTTTGGAAGGTTTTGAAATTTCAAACTCAGATCTACGAGCTATAGG
ACCTCAAATGGGAAAAATAATACCACCATGAGACTCAGTGAGACTTTTCGAGTCCA
GTAGTACCAACTTGGCCGGACGAATCGACGCCGGAGGTGGCGCGTGGGGCCCACGC
GCCGGCGAGGCCGGACCTTTTCCCGGCCGTTTCGGCCGGATTCCGGTGTATTTTTCA
AAAAAAAAAAAAGGTACGGTTGGATTCGTGAGGTCGTAAAGAGTCCAACCATACCTT
TTTTGTGAAAAACGGAGACCGGATGGTGGAGACGGTGGTGGTTGGTGGCCGACGGC
GGTGGAGGTGAGTTTTGATATACAATGACTATATTATCCCTTGACTAACGTTGACCG
TTAACATAGTAACGTTGAACGTTAGTTGACTGTTAGTTGACAGTGACCAAAGTTATA
TTTTTTAAATTTCGAGTGACCAAAGTTAAAATCTGAAAAAAAAAGAGTGACCAAACTG
ACATTTTGTCAAACTCGAATGACCAAAGTTATAATTGGCCTATTAATAATGCTATTT
GTAACATAACATTCATCTCATTCATGATAGAGATAAATGATGATTATAAATTTATGA
TATATTTTTAGCTGTCATTTGTAACTCTCTCGTATCATAAGTGAGATGAAAAATCATA
CCTTTTTTTTTTTTTTAAGGATTGCATGTCATTGACTCATTTGCTAATATAAGTTTAGGT
AATTTTAGAATTTGAGTAAAGGGGTTAGTTGTGTAATTTCAAACCCTGGATGCATAG
GGATTGATTGGCAATAAATGTAATTAAATTAAAATATCGGGGCAGTTTTAAAAAACT
AATCTCATGAACCTTTTTCAGTTATCCAAAAACTCTAATTATTATTATTATTAATATT
CTTTTTTTTTTTTGTGTTGATGTTTTAGGTGAAAATTCAATAAGAGAACCCTCTCTTTC
TCTCTCCCTCTCTCTCTAGATTGCAGGAAAAGCAGAATTAATTTTAATTTTCTAGAGA
GAGAAAGAAAAAGACAGAGAAAAAAAAAAAAAAAAAAAAAGAGAGAGAAAGGGTG
GTGGTGTTGTTAGGAGGGAAGTCTCTGGGAAAGAGCTTTGACCCTTTCTCTCTTTTGT
TTTAATCCAATTTCTTCTTCTTCTTCTTCGTTTTCAATCTTTTCACACGTTAAATTAAT
CCTCTTCATCCCTTTTCAACCCAATATATATCCTCTCCTTTCTCCCAACATCCGGCCA
TCTCATTTCAACTGTTTCGTCCTGATTTTTCCTTACATTTTTCTTTGACTTTCCCGGAA
AATTGAAGGATACTCCTGCTATCCAGAAGCTTTTTCATTTTCGGCACGAAGATTGGT
CACCTGGGTTTCAGTCAAAATCGGAGATTGGAATCTAGATCTTGAGATTTTGTAATT
TTTGTGCAAACATTAATTTCCAAAAATTTTGTGAAAAAAAAAGGAAAAGATATTTGA
TTGGTTTTGATCATTTTTGCGCTTTGAATATCAAAACCCATCTGCGTATTAAACTAAT
TGTTTGATTATACGTT

>NISI03G0956

TTTTTCTTGTAATGTCTTGTAGTTTCTTTTGATCATAATTTATTATAGAGATTAGCAGA
CTAAAAAATACATGTTTATTCTTCATAATTTAAATATCTCTAAACAATTATTTTAAGG
ACGAGTAGACGCGTGACACATAAAAGACCCTACACACGCACATACACACGTGGCAC
ATAAAAGACCACACGCAAAAACACAAAAAAATAAAAAATTACATGTTTACTCTTTA
TAATTTAAATGTATTTGAATAATTATTTTAGGGATGTGTAGACATGTGACACATAAA
AAACTATTCACACAATTTTAGAGATGAATAGACATGTGGCACATAAAAGACTACAC
GCACGCATGTGCACACACCCATCCACCCACACATATATAATAGGGTTAATTTAATTT
TAATAATTGTTAGACAGATCTAATTAAATTTAATCCATGCTAGTAAGATAATATAAC
TCTAATTCACGTTAGATAGGTTTAATTAATTTTAGTCTTTATTAGTAAGATAATATAG
CTTTAGTCTCTAAATCACAAAATAAATATAACTTTAACATTTGAATATATATATATAT

ATATAGTGTGTGTGTTTAAATCTAATCTCTTATTGTTTTTGTTTTTTACTTTAGTCAGT
TTTCTCCCACAAAATACATATCTGAAATCATATATCTAAATTTCTAATCTTTGAATTA
TTTTTCACCTCTTACTCGAAGAGAACCAGTCTAACGCATTATTAGTCACGGATTATAC
CATTCATAAGAACTTAATTAATTGAATAAGATTTGAAGTCTACCTACTATTTCATTAT
CAATTAATAAATACGGGTTGGTTTTTATTTAATTTTTTTTTTAAATAAAGAAAATAAA
ACAAACAAATATGGGTTTCCAAACATTACATTGTGACCCAATTCCATCTTATTCCAA
TCAAGTTATCATCAAATAGTCAAATGTAAGCATCAGGCAAAAGGAAGAAAGCTGGC
TGTTGGAGAGCTAGATGAGACCCACTTTGGTAATTAAGCAAACAAACACAGACACT
ACAATACATACAGGCCCCTCCTTATGATTCCAAAATTTTTTTGTACGTTATTCAAGAG
ACAACAAACACCTTGCATCTCTTTCTCTTCTTAATTCTCACTTCTCAAAAATCTTTTTT
TTTTTTTTTTTGGTTATTTGGTTTGATTATTTTTGGCTTCTTTCATGCCATTCCTAGAA
AGCTATGCTTTTAATTTCAACAAGTTCCTTTTACTTTTATTTATTTATTTATTTTTGAA
ATATACTTTTATTTATTTAATATATCATATCATTTTTTTTTAATACTTCAGATCCTTGTA
ATCTTGAGAAAGATCGTATAAATTAACAATCTGTCGCACATTCCTACGATAACATTA
TGATATATATTTGTTTAATATATGTGATGGTATTATCTAATAGAAGTAAATGTTAATT
TAATCAAGATCATTTTCGGTGGTTTATTGTTTATTCATGTAATAACATATAAATATTA
CTAAATTTACATGTAATGACAACAGTATTCTCTTCCCCTACTTTTATTTTTATTTTTTT
AATTTTTTGTACAAATGAATTTACCTTTTAACAAATTAAATTACTAAATAGGACATA
ATTTGTCTTAAAGCGGAAAAATTTACTCTATTTAATTTGAAAGAATTTTTTTTATAGT
AAAATAAACTATATATTTTAACACAGTAAAGCTCTAATGTACTTTTTTTTTTTTTTTTT
TTTAATGCAATGAGTTGTTCAAGTTAGAAGGTTGTGTCTTCTGGGTTGTGTGAATTAA
ATACATATTCTAAATGTAAATTGCAAGACGGCAAGGCGTATATTATTGGCTAATACG
TGACAAATACTTGTACAAATTTCAAGATTATCTTGGACCACTGGTGAGTTTTATAGA
CAACGATTAAGAGTAAGTTGCGAATATACACACAAAAGTATGGATACTAGGGTAAT
CCATTGTCCCGGAAGTATAATTACTTGCGGGTTGAATGGGATTAATCAAAAGTAAAA
AGGGTAAATATTTACGCCAGTTAATAAAATTATCCATTGTTTTTCATCGAAGCCTTCT
ACATTCTCTATAATATTCTTGATGTGTTGGATATGAATTCTAAGAGAAACACAACAG
ATAATTTTACCTATTTCTGTAAAAAGAAAAATTAATATTTTTTTAATTTATTTGAAAG
ATGCAACATGAAAAAAATAGAAAGAAAAAAAAAAAAAACCTATTTTTTAATTTATTT
TAAAGACCAATTATATAAGAATATCTATTTTTCAGAGAGTTTGTAAGGACGTACTAT
ATATTTATTCCAACGAGACTTCTGATAATCTTTTTGGAATTTTGGTGGAGAGTTACTA
AGACGGTTAAGTTTGGAGTTTAAAATTTAAATTAAAAATAAACATTTGGGAACAAA
CGAGAAATTGCAGAAAGTTACTTGGTGAATAATAGAATTTTTGCACACCCCGAGGG
GCTAAAAGTAAATCGAATAATTTAAATTAAGACCTGTTATCATTCTGTCCCTTCACT
AGGTGTTCGTTAAAAGACCCCAAGAAGAATTTTTTTTTTTTTTTTTTTTTTTTTTT
TTTTTTTTTTTATATTTTATTTTTACAAAATCCAAAAATAAATCCTCTAATCTAATCCC
TCCATTCTTTCCGCCTCTGTCATAATTCTTCCCCGTCCCGTTCCGACTGCTCAACCCT
GTCGACGAGCCGACAAAATTTACAGAGCCAAAGAAGACGACAATAAATTATTCAAA
ATCAGAAAACCCACTACCTAAAAATATATCAAAAAACTCATCACTCTTTTCAAAAGC
ATCGCTTCCCTCAACGTACATCCACACTCTACTTGCTCTTACATTGCCACTTTCATCT
CTTATGTTTCTCTCTTGCTTTTGCATCTTCTGCTCAGCTGTTAGTAGAATGTGAAACC
CTAACTAACTAAAATTAGTTAGGGTTTACTAGATCCTGTGTGAAAAAGTACAGCATA
CTTTAGCA

>NISI04G1685

ATACCATAGTTTAGCTATTGCAGCAGCTGAAGTTGCTTGGTTTGAATCTCTTTTCAAA
GAATTACATTTTTCGCTTACTAAGTCTCCCATTATTTGGTCTGATAACCAAAGTGCTA
TAGCATTAGCTCATAATCCTTTTTATCATGCACGCACTCGTCATGTTGAGATTGATCT
CCAATTTGTTCGTGATAGAGTTATTGCTAAAGATCTCAGTGTTCATTATGTCCCTTCG
TTGGAGCAACTCGCTGATATCTTTACTAAGGCCTTACCTCAACCACGTTTCTACACCT
TACGGTCCAAGCTGAGTGTTGTTACTCCATCTACTCAGCTTGAGGGGGAGTATGACA
GGAAGCTTACAGATTGAGTTTATGCTTTATGAGTTTACTCTGTTTGTTTTTCTGTTTTC
TGGTTTGGTTGTAATTTTTCTGCTTTTGTTTTTCCCTGTATTTCTCTTTTGTGCTCTTTG
TGTTGCTGGCTGTAAATCTCCTTCGAGCAAACTTATTCCTACTGACAGTTTCAGCAGT
AATAAGTTTGAGAGGGAGTATTGAGATAGTTGTTGTACTGGTACTCAGGAGGTTCTG
AATAGTATTATAAAATCTCTCCCACTAATCTGAATGATGTTGGATTAGGCTTAAATT
AGTTAATTAACAGAGATTTGGTTAATTAACTAATTTACTATAAAAGTCTACTCTAAC
TAATCATTTAGGTCATTCTAGAGAGTAAGCATTTCTTCATCTCTTTGCTTCTCTTCCTT
TCCAAAATCTCTTCTTCTTTCTACCTATGATTATACCATTCTAATAATTAAATACAAT
GAACATTTTTTTTGCCCATCACTTCTTATTTATTTATTTTTTAATCCTCCCACGTTTGC
CGACATCACCTCCTTCGCCACCGTTCATGCTTATGATTTGCTCCCTATTAAGTGAGAG
TGAAATTGTCAACCTCTACCTGCTTTGGGTTGTAACTGAGAGCTGTCTTATTGGGCAT
GTTCGGTTCGATACAAAATCATCAAAAATAACCAAGAAAAACCCAGCAAATTACAA
GAAGTAAGCAGAGGGTATAACAATCATGGTTGAAATCAACACCCAAGATAAGCAAT
TAAGATCAGATTGAGATGTGAAGAAAGATTTTACTTCACCGACTTAATTTTCGTTTT
AGCTTTTCGAGGTTCCTACGAGCTCGTCTAACCCGACAACGGCTACTAGCGAGCTTG
TGCAAAATCGTAGTTACGGCAAAACGTTTTGTCCAGTCCCCCCAGTTTTCGCCCATCT
CCTCCACCTCCCTTTTGCTCCTATGATAATTGGGAATGCGTTCGCACCATCTCGCCGT
CAAACATAAGACTTTTCCTAACGTCTTTCCCCATAGACAATGCCAACTGTTGCAGGT
CAACAGTAACCGTTCATCCACGTATGCTAGTGTTGACTCTTCAACCTCTTCATGTACC
ATTAACATACCTGAAAATCCAACAGAAAACAAGAACATCGTTACGGGGAATGCCGA
TGGGGTGGGTTGCCAATGAACCCCCTCCGATGCCTAAGTCAGTGTCGCAGTAAATTA
ATATTGCAAAGATCAAAAAAGATGAGAATTAGGGTTAGTTCAACTCTCTTTATATAG
CCTGGCCTTTGGTGTTTGAGAGGAGATAAAATCCTTCTTGTTTAGGATTCTCTCTTGA
TTTTGAATAATATTTGAATATCGCGTAATATCTAGTGAATATTTGGCTAGATATCCTC
TCTTTAATGCCTATCCGGGTCGGATCTTTGTTGGGTTTTATGGACCTCAGTGAGCTTA
ATACACTGAAGATTATATAAATACCCCTATCATATATATATATATATAAGATTCAATTA
GATGATAAATGCAAGCTATTAATGTATGAGGTATAGAATATCCATATTAAAATAATA
GGGACCTCTCTAACTTAAATATCTCAACACATTGAGAGTGGATTTGGAGAACCATTC
AAAGCATTTGATTAAAGCAATACACTAAATAGGTTGAGATAATAATTAAATTGTTTA
ATATTATTCTTAAAGTAAATCTTGACATAGTCCTTCTTGGGTCAAAGCAAACCTATA
ACTATTTTAATAGGAGCAGATTAAATCATATCGTTTCAGGTCAAAAAATAATAAACA
ATTGAGTTTAAATATTAAAATCGCTATTATTAAAAGAAATTTACTATTATTATTCGTT
TTGTGAGTAAAAATACAAACCAAAAGTCGTAGAAATAACTTAGATAAAAAGGAAAA
AAAAAATGAGCGTATTTATATTGTGACTGAACTATTGATAAATGTTGTTTTACTTGCC
AATCGGATGAGAGAAACAAGAAAGAGAATTCTAGTTTTTTTCTCTTACTTTTACTGT
CTCTCTCTCAAACTGATACACTTTGGGTATCATATTATTAGTGAGAAATACAAAAGC

TTGGTTTCATTGCACATCCAAGCGTTTGTAGTCCAACGGTTAGGATAATTGCCTTCCA
AGCAATAGACCCGGGTTCGACTCCCGGCAAACGCATTTGTTTTTTTAATTTATTTTAC
AGTCTGGCTGACAGATCACATACGGAAGCCCTATTCATTTATTTTCCGCCCAATATG
ACAGATCACGTTTACATCTTCTTTTTTTTTTTTTTTTTTTGAAATTACGTTTCTATTTT
TTTATCATGAATTCAAAGTCAAATTTTATTTCATCACCAAATATATCTTGCTTTCTTCT
CCCCTTTTTTAAGGACAAAAAAGCAGAAAAATGTAGTAAAATAATACTGCATGTAA
ATTGTAGAGAAGAGACAGGTTAACTCAAGACAGCAGCATAATCTTACTTTATTATCC
GCTAATAGCAAAAGGTTCCGTACTTATTTTCTCCATGTCCAATTTGGTCATTTCTCAA
TCTCCTCCTCCTTCTCTTTCTTCGTTCAGACAGCACCAACTGGTACGTGTGTAATAAT
AACTCCACTTTTGACTTCGTGAATCTCTCTCCCCTGTATTGTCTTGACTTTCCTTAACC
AATA

>NISI04G0955

TGATAATCAACCGTGAGTTATCAAGATATGATTGATTGTAAAATTAAATAAATAAGT
AAAACCAAAATGATTCAACCAATCAAATTTTGATACAGTTAGATACTAAAGTTGACT
AAGATATGCAAGTTAAAATTTAGAATCTAAGCAGAATTAATATCACACATCTTAGTT
ATATATTTATAGTTGAAATTACAAAGCGTATTCATTTAATTTATGTTACGAGTTTTAA
CTGGTTTGAATATTGCATGTTTCTATGAATCCGACCATGTATGTGATAAGAAAATGA
ATCGATCTCTTCTGTAATTTTTGAGTAAAGAAAAAAGAATATTATAATTTGAGATAA
TATATGAAACGCAGTCAATTAGAAAACATGGTCATTCGAAGATTTTTTGACATAGTG
AACAAAATATTTTTAATAGAAGTAGCGTTCCATTAGTTATTGTCATCTTTTTCAATTT
TAAAGTTAATCACAAGCGTGCATCTTAAATAGATTTGAGGTTCTAAATAAATCATAT
TTAAACAATTAAATCATCAAGTTAAGAAAAAAAAATAAAAATGAATTCCTTCTAAA
ATTTGAAGACGTGTTTTGATTGTTTGAATATTTGAAAAAAAAAAAAATGATAGAGATT
AATATATATATATATATAAATTTAAACCACCAAACATATCGTTAATCTAATTCAGTG
GAAGGAAATAGGAACAAAAATGAGAAGTGATAGTATTTGTTTAGGTCATCAGAATC
TCAAAAAGTCAAAGGTTGACTAGTTAAATAAAAAGGGTTTTTGGGAGATCAACTTTC
GTAGATAATATTTCAGGTATGTGGGTGGAAGTTGGCGTTGGTATAATTAATATAAGC
TTCGAACAAGTCAATGCGGCGGAGTTGTTGATAATATTCACATTGAACAAATCCATT
TCAATTCTTTTTTCTTCTCAAAAGAAATAAAAAATATAAATTTACGTTTCAACTTTTT
TTTTTCTCTCAATTCTATATATCAAGTAATTTAATGTTTCTCAGTCTAATAAGAATAT
TGGAAAATACAGACATAAATTTTTTTAACAAAAAATAACATTAGATAACCTCAATTG
AACTCCTAAAACAAATGATAAATACGTCAAACACTTTAATTCACTATTAGACTAGTT
TTAAATGTAAATAACGAGAAAATAAATAATTGTTCAACACGATCTTTTACAAATAAT
AATGAAAGATTAAATAAAAATATGAATTAACTTGAATTTTCACGTCAGAAAATAGT
AACAAATATACATCATTGACAATTGCAAAATATAATTTTAATAATTTTCTAGATGAT
CAAGGAGTTGTTAGACCAAATTGCAAACCAACGTATTTATTCTCGATAACGATAGAC
GTTAATTTCCTCTCAAATATCAGGGAATGATTTTTAATTTTTGTATTTTTATATTTTAT
ATTTCTTGATTTATTTATTTATTGTTATTTTTATTTTATTTCTTTTCATATAATAAAATT
TTTTATCTAACACTTGCTAAAACATCTGTATGCAAACATACGTATCAATATTAATCTA
TAAATATGTAATTTTTTTATGCATTTTTTTTTTGTTGAGAATATCTATTAATGTAAATT
GTTAAGTAGTTGAGTGAATACAATATATATATATGAAAATGTTATACATCCCAAATG
AAATTCCAAAACGTAAGATCCTAAATTACGTGGTTAATATGATGGTCAAATATAATA

AAAGTAAAATAAAATTCAAAATATTAACTAATCATTTTTCACCATATCATTTGAAAT
ATTATTTTTTAAGATTTTATTTGATATATATATATATATATATATATATATATATATAT
AAAGTGACCAACAAGAAATTGAGGAATTATAATTTACCTGTCACTCTAAAAGATTAT
ACAGCTAATGTAGCGTACAGAAATTGTCCTGTTTTGTGTGCTTGACACGTGTATTTTT
CACATTAAAATCAGGCTAAAAATTAAGTGGCGGTGTGGCGAAGCTACACACTACAC
CAATGTGGTGCTGTGGCATAACTACAAGCCTAAAAGAAAAAAAAAAAAAAAAAAAA
TTCGTGGCAATATGGTGAAACCAGAGGACCGCATCAAAAATATTAAGTGGCTAGTG
ATTTTTCAATAGCGTGATTTTGTCACACCTGATATTAATATGAATTTTCGAGAGATCT
AATCGGATAACTCTTATTACATTTCATTCATTAAGATTCTGAATTTTTTAACTAAACG
GTGTAATTTGTGACTTTTTAAACTATTGGATCAAAAACACAATCTAATGGTTATAAA
TTTTCGGTAGACTACAGCTAATGTATAATAGCAATTTCCAAATATCAATTATTTCTAA
GCAACTTTATTTGTTGGCATCTGTACCTAATTCTATCAAGTATCAATCATGGGTAATT
AAACAGGTAAACAACTTCATGAACTTGCGCCAAAATCATCCTCACCATTCCCTCATT
CCGTCAAATAGAATCAACGGTTAATATCTCCCCTAGGTCCACGTCATCATTACACTA
AGTTTCTCAAAATAAATTAATATTTTAAAATAATGTAAAAAAAAAGAAGAAATATT
ACGCAAATTTATTGGTAAATAAATAAGTAAGGATAATGATTAGCCCACAGTACCAA
TTGACCCAGTGTATGCTAGGGGAATGATGATATATAATATTCATCTTAATTAATTAA
TTAAATAATATACCAAATATTTATAAATAAATAAATTAAAAATAAAAATAATTACTC
ATAGCTTTACAGATATCTGAGGGGTATTATTGTAATATCAGTCCATCTAATTCAACTC
TCGAAAGTGGTTGATGACTATATTGCATCTGTCAGCCATTTTTTTCAAAATTCCCATT
TTTCTCAAACTGACCCGCGTTTTCTCTCCACCCGCCACCTTCCACCACCGTCAATGGC
AGCGGTCAGCACTAACTGCTGCATTCTCTAAAACGCACCGTCTGACCCTAAACTCTA
ACTGAAAACGTCATAGCTTAAGG

>NISI06G0414

GACAAAATATATTGTTATTGTAGTAGACACAGTTAGGAGCTTAAAAAGAGGGAGAA
AAAAAAAAAAGAATAAATAAATAAAACAATGCACCGCCGGCACCGATGGACAATA
GGCAACCAACGGGCTGTTTTAATCAGACGAATTGATGAACAGAGTAGCAGAAATAT
ATCCGACGATTCCAGCTACATATGAGAAACAACTAGTACAGTCGAACTCATCCCTTT
CTAAGGAATCTAATGTTATTCCCCGATTCTCCATCGGGGTCCTGATTCAATAGAGAA
TCGATTACTCCCTTTGGGTCGTTCCTTACCAAATGAGGCCAACCGCGGTTGTCGATCT
CTGATCATAGACGGGGATGCGGCCGTGGTTCCAGTTGGGCTGCCACATTAGTTTTTC
CTTTTTATTTTCAATTAAGTATTTGAAAAATAATAATAATAATTACCTAGTTTAAATG
AGTTTGTACGTAGGGATGGACGTTGGGTTAATTGGGTTAGTTTGGGTCCGATTTTATT
GACCCGTAAGTATTTTGGGTCTAAGAAAAATGATCTAAGAAGTCTGAGCTTTTTCAC
TTACCCAACTTGGGTTGGGTTAGTTTGGGCGGATCAATTGAGTTAACTTGGGTTAAG
ACTTAGTAACAGTAAAGTTGATATTGGCTAGAAGAAAAAAGAAAATACAAAAAAAA
AAAAAAAAGAAACTGTAGAAGAATTGGGTCAAAGAAAAAGAGCCCAATCTTGGGTT
TGGATAATACAGACCCGTCCTTACACAATCGGATTGGGTTGGGTTACTTGGGTCATA
ATTGGATCTGGGCATTTTGCCCACCCCTATTTGTATGTAAGGATAGATTTTTTTTTAA
CCGTTAGATTAGATCTAACGGTCAGTAACCATTCCGGTACCAAAACCTAGCGATACC
ATAGGCAGACCCAACTTAAGGGTTTTTTTTTTTTTTTTTTTTTTTGGGTTAATAACC
TAAAGTTGTAGCTAGGGATGACAATTTCTAGGGTTTGGGTTCAGGTTTAAGGGATCG

AAACTCGATCGGTTCAAAATTGAGAATTTCAAAACCCATTTTTTGACCTATGTTTATT
TTCGGGTTAGGATTCGGGTGACCTACCAAAATTTTTACGGACCCATGAGTTGCCCAA
AACCCACAGGTTCGGGTTGGTGAGTCAAATTTTCTGGATAACCCAGGCGTAATTTTT
TTTTTTTTTTAAATTTCATTATTTTTGTTATAATTTTATTATATTTGTTTAGTTATTTTA
CATTAGTGTTTAATTTATACATAATATATTATAATGTGTGTGTATGTATTTATGTATG
TACTAATAATAATAGTAATAATAATCAAATATATTAATATTTGAAAAAAAAAGGTGGT
GGATTCATTCAGGTTGGGTTTAAACTTCTAATCTACCATCCACTTATATACAATTTTG
GGTTTGAAATTTTAACCAAAAACTCTAAATTTTCATCCATCGGATCGGATGGATCAA
ATCTAAATTGCTCAGGATCGAGTAAAACCCACAGATTTTTAGATCCGCAGGTTTAAA
TTGTCATTCCTAGTTGTAGCACCCGTCTTACAATGTTATGTTGACGGTGTAAGCGGCG
AGTGGACTTTGATGGACCAAACTTGTCTTGATGATTTAGTACTGACGTGTAAAATAG
ATACTACTAAATAATATATAAAAAAGAAAAAGGAAAAGGCAGAGCAGAATCTAGA
GGAGAAATACCAAACCCATCCGTGTCCCCACTGGTTTCCACCCAATTAAAAAAAAA
AAAATATATATATATATATATATATAGTAATGATATATAGATAATCTACGTATTTAATTT
TGGTAGTCAATTATAACTTGATAATATATAATTAAAAAAAATTATTTTAATATTACTT
ATTTATCATTTATACAACCAATAAATAAGTAATATTAAAATAATTTATTCTAATCAC
ATATTATCAGGTCATAATTAACTACCAAATATATAGATTATCCATATATCATTACTAT
ATATATATATATATATATATATATATATATATATATATTATTCATCCACCTTCCTAAATT
AAATGAAGGGATAACTCATAACTACCTTTTAAACCCTTAAAAAACTTGATCACATTT
GATACTATATAACTTGAGAAATTATACTTTGATATTTCTTCTGACTTAAATTAATTAA
TTTATTATGACAAAGTTATTTTTCAGTATTATTAAAAGGAGCATGTACAAAAATAAA
AAAGACATAAATGTCATTTTCAGTCAATTGAAACCTTTTTATACTTATATCAATATAT
CAAAAAAAAAAATTATATACTAATATATCTACCAATTTTAATCGATATAAATATGAA
TTTGAATTTGAATTTGTTTCAAAAAAAAATATATATATGAATTTAAATTTAATTTTAAT
CTTTGTTTCATAACATAATGTTCGAACTCTAACAATAGATAATTAGAGGTATAGGGT
TCGAAGTATTTAAAAATTACTTTAATGAAGAGTTGTGTGATTGAATTTGTATTTGTTA
TTGTGGAGATTCTAAGAACAACAAACTTTAATTTTTGCTTTCAGCTATGTATTTGTTG
TAATACACTTAAAATCAAATCGATAACAGTAAGAATATAAGATTATTGATTTTAACA
GACAAAAAGGATTAAAATAAAATTTTGTAAATTACAAGAACTAAAATTGTAATTAA
GTTTATTTTTTTAGGGATCAAAGTGTAGTATCCCATAAAAATAAATAAAATAGATAA
ATAAAAATTCTTCTTCCTTAATTCCCTCATTAAACAAAACTAAAAAAAGACAACATT
CTTTCTCATTCTTCCATCTCACGACCAAAATCGAGAAAAGAACGACAAGATTAATTT
TCATAACAATAATCCACCAACAACATCTATCTCCATTTTCATTTGAAATTCTAAGGA
GAATTTCCAGTTGCTACAGCTAAAAGAAAACCTTCATAATAGTAAGGGATATCGGA
GTGTGATCTGAGATTATTGCTAGAA

>NISI06G0419

GGACAAAATATATTGTTATTGTAGTAGACAGAGTTAGGAGCTTAAAAAGAGGGAGA
AAAAAAAAAGAATAAATAAATAAAACAATGCACCGCCGGCACCGATGGACAATAG
GCAACCAACGGGCTGTTTTAATCAGACGAATTGATGAACAGAGTAGCAGAAATATA
TCCGACGATTCCAGCTACATATGAGAAACAACTAGTACAGTCGAACTCATCCCTTTC
TAAGGAATCTAATGTTATTCCCCGATTCTCCATCGGGGTCCTGATTCAATAGAGAAT
CGATTACTCCCTTTGGGTCGTTCCTTACCAAATGAGGCCAACCGCGGTTGTCGATCTC

TGATCATAGACGGGGATGCGGCCGTGGTTCCAGTTGGGCTGCCACATTAGTTCTTCC
TTTTTATTTTCAATTAAGTATTTGAAAAATAATAATAATTACCTAGTTTAAATGAGTT
TGTACGTAGGGATGGGCGTTAGGTTAATTGGGTTGGTTTGGATCCGATTTTATTGAT
CCGTAAGTATTTTGGGTCTAAGAAAAATGATTTAAGAAGTCTGAGCTTTTTCAATGA
CCTAACTTGGGTTGGGTTAGTTTGGGCGGGTCAATTGAGTTAACTTGGGTTAAGACT
TGGTAACAGTAAGGTTGATATTGGCTAGAAAAAAAAAAAAAAAAAAAACTGTAGAAG
AATTGGGTCAAAGAAAAAGAGCCCAATCTTGGGTTTGGATAATACAGACCCGTCCTT
ACACAACCGGATTGGGTTGGGTTACTTGGGTCATAATTGGATCTGGGCATTTTGCCC
ACCCCTATTTGTACGTAAGGATAGATTTTTTTCAACCGTTAGATTAGATCTAACGGTT
AGTAACCATTCCAGTACCAAAACCCAGCGATACCATAGGCAGACCCAACTTAAGGT
TTTTTTTTTTTTTTTTTTTTTTTTTTTGGGGTTAATAACCTAAAGTTGTAGCTAGGGA
TGACAATTTCTAGGGTTTGGGTTTGGGTTTAAGGGATCGAAACTCGATCGGTTCAAA
ATTGAGAATTTCAAAACCGACTTTTTGACCTATGTTTATTTTCGGGTTAGGATTCGGG
TGACCTACCAAAATTTTTACGGACCCATGAGTTGCCCAAAACCCACAGGTTCGGGTT
GGTGAGTCAAATTTTCTGGATAACCCAGGTGTAATTTTTTTTTTTTTTTTTAAATTTCA
TTATTTTGTTATAATTTTATTATATTTGTTTAGTTATTTTACATTAGTGTTTAATTTAT
ACATAATATATTATAATGTGTGTGTATGTATTTATGTATGTACTAATAATAATAGTAA
TAATAATAATAATAGTAATAATAATCAAATATATTAATATTTGAAAAAACCAGGTGG
TGGATTCATTCAGGTCGGGTTTAAACTTCTAATCTACCATCCACTTATATACAATTTT
GGGTTTGAAATTTTAACCAAAAACTCTAAATTTTCATCCATCGAATCGGAAGGATCA
AATCTAAATTGCTCAAGATCGAGTGAAACCCACAGATTTTTAGATCCGCGGATTTAA
ATTGTCATTCCTAGTTGTAGCACCCGTCTTACAATGTTATGTTGACGGTGTAAACGGC
GAGTGGACTTTGATGGACCAAACTTGTCTTGATGATTTAGTACTGACGTGTAAAATA
GATACTACTAAATAATATATAAAAAAGAAAAAGCAAAAGGCAGAGCAGAATCTAG
AGGAGAAATACCAAACCCATCCGTGTCCCCACTGGTTTCCACCCAATTAAATATATA
TATATATATAGTAATGATATATATAGATAATCTACGTATTTAATTTTGGTAGTTAATT
ATAACTTGATAATATATGATTAAAACAAATTATTTTAATATTATTTATTTATCATTTA
TATAACCAATAAATAAGTAATATTAAAATAATTTATTCTAATCACATATTATCAGAT
CATAATTAACTACCAAAATTGAATACATAGATTATCCATGTATCTTTACTATATATAT
ATATATATATATATATATATATATATATATATATATTATTCATCCACCTTCCTAAATTAA
ATGAAGGGATAACTCATAACTACCTTTTTTAAGCCCTTAAAAAACTTGATCACACTT
GATACTATATAATTGAGAAATTATACTTTGATATTTCTTCTGACTTAAAATTAATTAA
TTTATTATGACAAAGTTATTTTTCAGTATTATTAAAAGGAGCATGTACAAAAATAAA
AAAAGACATAAATGTCATTTTCAGTCAATTGAAACCTTTTTATACTTATATCAATATA
TCAAAAAAAAATTATATACTAATATATCTACCAATTTTAATCGATATAAATATGAAT
TTGAATTTGAATTTGTTTCAAAAAAAAAAAAATATATGAATTTAAATTTAATTTTAATC
TTTGTTTCATAACATAATGTTCGAACTCTAACAATAGATAATTAGAGGTATAGGGTT
CGAAGTATTTAAAAATTACTTTAATGAAGAGTTGTGTGATTGAATTTGTATTTGTTAT
TGTGGAGATTCTAAGAACAACAAACTTTAATTTTTGCTTTCAGCTATGTATTTGTTGT
AGTACACTTAAAATCAAATCGATAACAGTAAGAATATAAGATTATTGATTTTAACAG
ACAAAAAGGATTAAAATAAAATTTTGTAAATTACAAGAACTAAAATTGTAATTAAG
TTTATTTTTTTAGGGATCAAAGTGTAGTTATCCCATAAAAATAAATAAAATAGATAA
ATAAAAATTCTTCTTCCTTAATTCCCTCATTAAACAAAACTAAAAAAAGACAACATT

CTTTCTCATTCTTCCATCTCACGACCAAAATCGAGAAAAGAACGACAAGATTAATTT
TCATAACAATAATCCACCAACAACATCTATCTCCATTTTCATTTGAAATTCTAAGGA
GAATTTCCAGTTGCTACAGCTAAAAGAAAACCTTCATAATAGTAAGGATATCGGA
GTGTGATCTGAGATTATTGCTAGAA

>NISI06G1911

TTCTACACTTCTTGCTACTTTATACTTTACGCATGTGTTTACTTGATGTATGCTAGCTA
GCCTCCGAATTAGGCATTTGTGTTGTAGCGCATTCTTGGTAGGCTGGTCAAGTGCGA
CTTGGCACCGCACATCCACTCGCTGTCAATAGAGGGCTTTGAGGTTGGCGGTGTGAC
ACCTTCCATGCCTGCTGGGCAGAAACAGTAGCAGTCCTCGGGCAAGCAGCAGCAGC
CAAAAGACCCTCCAAAAAGGCCATTGACCAGTCGTGGCAATAAAAGTTTCTGAAGA
TGTTTGAATTTGAGTTCTGATCACCATAAATGTACAAGTTTCTAAAAAACCAAACAG
TTCGTAGCCTGCAAAAATGTCAAGTGATTTTGCAGGGCAAATCAACCCCCCTTTCTT
TTCTCCTTCCGTTCAAGCACGGATTAGCAAATGGGGTATTTTCTCAAAAAGGAAAAA
ACTGTGGAGTATCCAGGAATGCATAGAAATTCTATTCTAGGCTCGCGTTACACCGGG
GTCAAATTGGATTAACGTCCCTGCCACAAGAATAGTCTTGCATAGGTAGAGAAGAT
ACCTACAAACCTTCCATGTAAACAACTTGATCTTATTTGGAACAGTTAAGTACAAAA
CCAAATTCCACAACTCCGCCAAACTAGAAGAACCTGCCTTCTCCAACCTTTCTACAA
ATGGCGTCAAATTATAAGCACTCCGCACCGAAAATAATCCTTTTATTTTATTTTTTTA
AAATCAAAGAATTAGTATAAACTATCTTCCCGAGAGGAAAAACCCAAAGACCCAGG
CTGGCCAGGCCCAAAAAAAAATTTATATAAAAGAAAAGGAAGAAAAAGAAAAAAG
AAGGAAAAAAAAAAAGAAGGGAAAAATCCTACCCGGAAAAAAAAGAAGGCCTGATT
TTTTGCTACTTTATTTGTATTCTGATTGTCTTATTGGACTTCCTTCTTAGAGCTTGTTT
TTTGTTGGTTTGGGCTTTTGCCCCTTTTATTAACAAAAAAAAAAAATAATAATAATAAT
AAAATAAAGATAAAGGATTTATGTAAAATGATTTATTTTGTATAAAATATGTCTAAA
AGTAAGATTTATTACGTCTTCCTAACAAGAAAAACAATCAAATTTCTCTTATTTTTTA
TATTCAACCAAAAAAATTTTACCCAAAAAAATTTACAGCCCAGGCTGAAAAAAATTT
ATGGTTCCGCCCCTGATGGAACGGATAGACTAAATATTAGTTGAGTATAAATATTCA
ATAAACAACGGTAGATTCCATCTTCTAACCAAGGAAATATACGAGCAACATTAGGA
TCGGAGTGTTGAAATTAACAATCGACTAAAAGAAGAGGGGTCTTGGGATTCATCTGT
GAGATTTCTCATTAACAAGCAGAGCAACCCCAATCCTCCATATGAATCTTCCACCAC
AAACTTCTTTCTCCAAAAAAAGACTCCACATAAAGGACGGAAAACTACCCAACACA
CCACGCAAACTAATCCTTTCTCTCTTGCTAATGTTGTATGTTCATATTAAGATGCTAA
TTCATATGATATCTTTATGTGAATTATCATATTGTTAATGTAAATATGTGGGCATATT
CATTTGGACATACATAGTTGAAGAAAAGAATTGAAAAAGATTGGTTTGTATGTTAGT
ATGTATACTTAATGTATGTACAAATTTTCTTTTTATGTAGCACAGTTCGCATGTCTTG
TCTTATTTTTGGACATATCTACTCATATTTTTAAATTTTGTTACTCAATATAAGAAAA
CAACTGACCACTTCTCCGCTTTTTAGAGTCTATGAATAATAAAAATTAAACTTTAAA
CTTTTATATAGAAGTAAAAATGTCTCATCTGTTAGACCGTTGTCATTGGCTCTTTAAC
AGTTAAATTGAAAATGCAAAGCATTCCGGTCATGATTTTAATACATTATTGGATGAT
AGAGTGCACAACATTCTCATTATGATAAGAACAAGGAACGACGATATCATGTCCTTT
AACCTGAAAGTGTGTCCCCTTGGAAATTGTATGAAATGCTTTGCTTGGGGACTTTAT
TGGAATTAAAATACTATAAAAAAAGGGGAGGGGGGGGGGGGGGGGGGTGGAAT

TCCACTTTAGAAACTATTTTCTTGTCTAATCTCACTTTGAGATTCTTTCTTATTTATTT
ATTTTGATGACAAACACATGTTTTGCTCTAAAAAAAGGGACCTCAAATCAGTATATT
GACCAGAACACCCTTTGTTAATTATATTTTCTTTAAAAAAATCACATCTTTGTCCCTT
TCCAACATCAACACCGGCCACCACTAATAATTGACGAGCCACCGTCGAAATCCGAC
GATTATTTTTTTCAAAAAAATATCCGCAAATACCAGCTTATTCGCGTGGAGGAGACT
ATTCTGGTGAGGAAACTATTTTTTATTTCTTTCACGTGGAGGTGACAGTTTTTGATCT
CCTCCAGGTGGAGGAGATGTTCTTCGCCTAAAGGAGACCGCTTGCTTTCCTCTATGA
CAGAGGAGATCGATCTCCTCTATTTTTTTAATAAATATTATTATTAAATTACATAATT
ATTTTTTTGAGGTTTCATTTTGTATCTTTCTGGATCAAAATATATATATTTTTTTAAAA
AAAATCTCAAATTAAGATTAAGTTAGAAAATAAGTGCCAAAGTGAAATTTCTAAAA
GCTTGCTCCTCACTACACACGAAATATGAACCAAAATTCCTCCTTCCCTCCTTATCTC
TCCCTCCAAAAGCAAAGCGTGTGAAAACAGAAAGTGAAGGAATAATACCCAGACCA
TCCATTAGGCAAAGGTAAAGCACCCAAAAAATACGAAAATAAAAAATAAATTTCAT
TTTATTCCAATCAACATCTAAAAGTTTCTCTTCTCGTTATGATTTCTGCAACACCCTA
TCACCACCGCCCTGGAAATTTTTTTAATTCATTTTCATTTTACAAGAGTTTTTGATTGT
AAGAATCGACCAATCATTCAGCTCTATCA

>NISI07G3121

CGTATGAAATCAACCACGACCTGGTCATCGTGACTTCACATCATCCAGAATATGCCC
TTTGATCGAATCCAACCTTTTTTTTTCTCTTTTTTTAAATTTCTATGAGAAGAAAATA
ACATCATGGGACTTTGAATTATAACAATCAACTTAATACTGCTATCACATTATTAGTT
GTCCTGATGGTTTTAGTTTTCTTCATATTAAATTTTTTGATATTACAGCGAGATGTCTT
GATTACTTTTCGTATATATTAATTAACTTTTTTCAAGATAAAAATAGTAACGAGAAA
TTCTTTCTAATAATAAAAAATTTAAATTTTATACTCTAAATTTTTAGGTTCGAAGAAC
AAATGAGACTCGATATGTTATTACTGAGTTAATACGTTGTTCTCCATATTAAATTTTC
TTGTTAAAAGAAATAAGAATAATTAATTAAGTTATAACAAGGTTAATTTTTAAAGAT
CACTATTACATAAAGTAGACTCAATCTTATTAAGACGAAAGATTTAATTTCATTTAT
GTATTTTATTTAATATTATTATGCACTATTTAGTTAACTATTTATCGTTTTTGTTTAGT
TTAGTCGTAAAAAAGGGGACTAAAATAATAGCTTTTAAAGGGTGATGGATCACATA
AGGAGAATTTTTCTTGACGAAATAAGCCAATGTAATATTCTTATTGATAAGACACAT
ACTAGTTAGTTCTTTTTTCTTTTTTCTTTTTTTATGTGTATTGAACCAAGAAGTATATT
CCTAATTGGGCTACAAGCTTATCAACGTAAATATATATTTTATAAGTTCATTTATTAC
TAAACATATATTATGAATTTTATCTATAACTTTAATCATATGAGAGAAAACTTATAG
TTTTATACAAAATATAAATACTTTCAGAGAAAGTCTATACATATTTTATTTTATTTTA
ACTTTTTTTTTTTTTAAATTGACACTTCTAAGTTACAAATCTATTGAGATACGTCAAA
TAATAACTAAAATATAAATCTTATATAAAACGCTCTATTTCAACGAAATCCTCAACA
CACAAAAGATAATAAGAAATATCATTCTAGTTAGATTAGTATTTTGTTGACATTTTT
ATTTATAAATTGATTTATTATTACATTGATGTTTATGTATCACAAATTTAATGAGATC
TTATCTAAAAATTTAATATATTTTTTTTTTGAGGTTATTAATTACAAGCTATTACAAC
TACTGGGTCCTAGGCTAGTGATAGATTTACACACCGACACACACAACCCAGAAGCT
ACTATCCAGATAGGAAAGGTTGTCCACTCGACTGCTTTTAAAAATACGACATTCGAA
AGAACTGCTCATTCGATCGTTTTAAAACTCGGGACAGATACCTTTGATCTTCACGTT
GAGAGAACTAGAGGTAAACATTTGGAGCAAGCTCCAAGATTCATCTAAAAATTCAA

TCTTATAATAGTAAACGTGTACTTTTATAGAAAATAATAACAATTTGGAGAGGATAA
AGTTGTAAACATACAAATTAATGTGAACACAAATAACTTAAAATAGTGGGGTTGGT
AAATTTAACTCCAATAACTCGCATAGGTTTTTCAAGTTTTTACAACTTTACAACAGCT
AGCCAAATATAGTTGAAAAGTCAGTGCAGCTTTTCAGACAAACACTAACACATAAA
GTGGGGTACCGGACAGCCGACCTATAAGAATTTAGTACCCACACTGTCGTTTCAACA
CTAGAAAACGCAAATACTTGTCGTTTTAGTCATGACCAGACAGCAACAGCTTTCTTT
TGGTTTTCTCAGTTTAATTTGTTTTTCATAATAACTAAAGAACTTTCTCTTGGCTACA
AACGTAAGCACAATTATAGAGAGCCACTCACTGTTTTTATTATATTAATTTCATACA
ATAATAGAATAAAATAGGACAAATTATTACATCTAATTTTGTTAATCAGTGAACAAT
TATAGTTTTTCTCCTTGCATAAATTAATCTAGTTTATATATATATATATATATATATAT
ATATATATATACACATATATATATATATATATATATATATATATATATATATATATAT
ACACATATATTTATTTTTTTATTTTTTTATTTTTTTTTATATGTTACTTGGGAATAAAA
TGTTAATACATGCATAAATATATATAATTTTCTTCGACAACAGTTGTATTTTGAATAT
TGTATTAGAATTCATGTAAAATAATTTAATCAGTGTGAAGTTATTACCATATAGTTA
GTACAACAAATGAGCAAAAAACTGTTTTCCTTGTTAGTTAGCATTCCCAAATCTTAT
TGGGATTTTGTCAATAATTTTTCAAAAAATAAAAAATAATTTTTTATAATGCGTTCTG
CATTTAATAAACAACTATAGTTATATATAATATATATATTATAAAATATATGATATA
AAAAAAAAAAAAAAAAAAAAAAAAAACACGTTAGAGCAACTGTGTTAGGCTCAAA
GGACCATCTCAAGTTCTCAACTACTTAGCTATCCGATAATTACTTTTCCATCGCTAGA
TTAAAAATCAAGACATGTGTTTTGTGTTTTTTTTTTTTATTTTTATTTTATTTTAATTTT
TAATATTTTTCCCCCACAATGACAATTGTCTATGTCAAAACGGAAAAAAATATTTAT
TTCAGAAAACGAATAAAAAAACTGAAAAAAGAAGAACATAAAATAAAATAAGATG
CAATAAAATTAAGGAAACAGGGGGTGGTGTATGGGTTCTGTCTTACAATTTTTCAAT
CTCTTGTAAAAAAATAAAAAAGAAAAAAAAGTCTCTTTTGACCACCACCACCACCA
CCCTTCCAATCTCTATTTCTTCCCTTCTTCCTCCTATCTGCTACCTCCTTTCCTTGTTCT
CATTCCAATCTTCATATCTGCAATAATTTATCTTCCCTATTTATGATTTTTCCATGGAT
TTCTTCTCTTTATAAACCTCAATCCTCCTTTTGACCCCTTCCCCTTTTAACTTAAACAA
ATCCCATAAAC

>NISI07G1055

TTCAATAAGATAAAATGTTAAACTAGATTTTGAAGTTCAATTATGGACATTTAAATA
ATTTAAAACTATATAAAACACTTTATTAGAATTAAAACCAAACACCTTAAACATAAT
CGCTTTCCTAAAAATATAATATTACATCCAAACATCAAACTGGTACAAAGTGTATTG
CTTCAAATAAAAATGCTACACAAAAAAACACTTTTAAATGGACCCTTAGTTTCTAAA
AAAAAAATAATAATAATAAAACTTTAATCCATTCAGTTGAAAAATTAAATTTTGATC
TTTATTATCAGTTAAAATTAATATATTGGAAGTTGTAGAGATGTTTTTAAATGATTAA
AGTTTAATTGTTCAAACTATATAGAGTAAAATTTTATTAATTTTTTTTTTTCAGGTAC
TGAAGTGTAATTATCTCTCAATAAATAAATTTCCTCAAAAAAAAAAAAAAAAAAAA
ATCCCTCAATAAATTGGCTCTGGTTATTTGTTCAAAAAAGAAAGTACTTGTAACAGG
TCCATGAAAGGGTGGCTTCTTGATAACGGATATTGGATTGGGCCCGGGGGCGGATCT
ACCTTCGGCTAGGTGGAAGAAGAAGCCTTTTTTTTTTAAAAATAAAAATAAAATTCA
AAACGAGTTATTAAACATTATTAAAGAAAAAAAAAAAAACTCAAATCTCTTTCCCTGC
AACCTTTAATGATTCGAATTTTGTTCTTCATAAAAGAGAAACAGAAGATTATACTAC

CTGACTGAACAATTAAATGTATGATATTGAGCTTGGAGAGTCATACCTAAGTCCAAT
ATCATTTTAAGGCTATCATGATAGAAAGATCAGGGGATCATCAGAGCAAAAAAAAA
AAAAAAAAGAGCCCTCAACTGGATTAGCCAGAATATTAGATGATTATTAGGCCAAG
CCTAAGCCTAATATCATTTACTAGAAAGGGTTCAAAGATACTTAGACGGATCAGCCA
AGAAGCTATATAGACCTTCAAAAGTATGTTCAAGGACGCTCAGACAAACCAGCCAG
TAAGTCAGATGAACCTGTAGAATCAAATTAAAGAACACTTGAGCAAATTGACTAAT
GAAGCAAGTGGATCTGAAGCTAAGGGCAAAAGGGAATTTACTTGGTGAGAGATTTA
GAATCTTCTAAAAAATTTTAACGGTAGACAATTAGGATAGGGACTTATAACTAAAAT
CAAAGGACCAAAAGATACTATTTTGGGGAATTAAGGGGTATTATTTTGTAAGTTTTA
TTGGTATATAAATATCACGTCAACTGAGGGAAAAAGGATGGCTTTTTGCAAGTTTCA
CCATGTTAACATATCCCTCATAACCTTTGTATCTAATTTTACACCATTAATAGTATTT
TCCTCTTAATTATTTTGATTTTTCAGTTATTCTTCTTAATTTTTAAATATGTGAAGATA
ATAGAATCTTTTACAAAATCTTTTACATTAAGGGTTTTTTTGCTAAAAAAAGCCTCAA
TCTTTTAAAAAGTGTCTAACAATAGGAGACAGGGACGTAGCCAGGAATTTTTTTTAG
GGGGTAGACTTTTTATTTCAAAAATTTCAAATATGATAAATTGAACTTTTAAAATTTT
TTAGGGAGTAAATTGAAATTTTTTTAGAACTTCAGTGGTAAATTGGTAATTTTAAGA
TACTTTAGAGGGGGTAAACTAAATTTTTTTAAAATTTGTTAAGTAATTTTGTAGTTTT
TAGAAGGTGAGGAGGGGCAATCACATATTTTTCAAAAATTTGAAGGGAACAAACTG
AAAATTTTTTAAAATTTTTTAGATAATTTTACAATTTTAAAAAATTGAGGGAGGGCA
AAGGTCCCCCTTGCTCCCATGTGGCACCGTCCCTGTAAGGAGACAATGTGTCTAATA
AAAGGGATAGAAAAGTTTCATACATGGAAAAAAATCTATTATCATATATTATAACG
ACGGGAAAACTCGATTTTTAGATTTTTAAGGAGGCTAGAAATATATTTCCCATTTAC
CACTGTTATACTGTTAACTTGTAGGCTTAAAAAAAGAAGAAAAAAAAAAAAAA
CACTTCACACGCCGGTTGTACTGATACACCACCCCTTTATACCCTTCGATCATTCCAA
ACAAAAGTCGGACGGTTTAAATTCATTATATAGTTTTAAAATCGTCAAACTTTCCTA
CATTCACTTCAAGCGCGTGGCTACCGGAGCTCCCACCATCGCCTGTCGGCCCTGTCC
CAATCCTGAAACCACCATCCGAAAATCACACCGCCACCCATCCCAATCTCAAAACCC
ATCCGGAAATAACGCTGCGGCCCATTCCGATCTCAAAACCAACCGGAATCACACCA
CCGTTGCCAAACGACCCCGCCGCTTCTACCACCATGATAGCACCGCAACTGCTACCA
ATATTCACCCCAAAACCAACACCCCTGTCAGCACCGGCAAAAAGAAAATTGGCCAA
AAAGCAAATGAAATCCAACTTGGAAATCAGATCTCAGAAAGACAACCCGGTTAATT
ATTTATTTACAGAGACTCTAAAGCAGTCGCAAGATTCTTCAAATGATGCGAAAATGA
AGAGCAGGCTAGGTAAATCTGATCAAAACTCCAGTATATTTTTCAGTTTTCAATTTT
ATTAAATAAAATTATGTATAAAGTGATTTGCATTTACTAGTATATATCCTTGTCAAG
AAGTGTATATTGGATCTTTAAAACTACAAGAATTATTTGTGAATTATTCTGATGTGG
GAGCTAGAGTACAGATGATATAAAGACAAAGTAGGCGGTTAGAATTGAAATTATTA
ATTAGTCATAATTACTTGCTCTTATAACAAAAATGGAGGGAGGAATAGTTGACTATG
TCTTTAGTCTTTGTCTCTTGTTTCTCAGCTCTATCCACCAAATTATATTCCCTTTGACT
ATATTAATATCGTAAACTTGTCTGGAAAGAAAGAAAAGCCGGTTTCAACTATCAGTT
GTGCTTCTCATTCTCATAATCAAGGTTTCTTCTAGCTG

>NISI07G2891

TAATAATTATTTTTATATGTTTGGATAATGATGGTCCTTATAGATATAGATATTTATG
TGGTCCTCAAGGAGAAGGAGATCAATTAGTGATACTCGAATTTTGATTCTTAAGTTC
GGGTCTTGGTGGTTTGAATTATTAAAATTTTTCATGTCATTGTTTTTAAAAAAAAAA
AAAAAAAACTCATATTCTCTCCTATCTTCTCTCAGTCATATCCGAGTTTGATTTTTAT
TATAATTTTATAACATTGGAATAAATCTTTCTTGTAAAGTTCTTATATAAATTTCTCT
TTTTCTTCTTTAAGGAAATAGAGGTCAATCTCTAGAAACTATCCAATCTCCTAAAGG
ATGTTTAATTTGCTGGTAAATTGACCAAGTCTAATTGTCTCCACTTTAAAGATGATTG
GATCTTTTTTTTTTTTTTTTTCCGGTGGAAGAGATCGTATTTTGTTTCTTCCACTAGTA
AAACAAACTCAACTTTTTTTTTTTTTTTAATATTAAAAATAAGAGAGAATATCAATCC
CTTACTCGGCATTGTTGATATTATTCGCTTGGTGTCCGTCAGATCTTTTCAAAGAGAT
CTAACGTTCCAAATTTCATAGTGTACTAATACATCCATCGACTATAACCGAACAGAC
TAGAAATCTCCCATAAAGATCAGCCTGTTATTGACTGTTAGCGGCTGCCAATGCCCG
TACCTTCATTGTTGGCACTTCAAGAAAACGTGCGTTAATTATATAACCTCGAAATTCT
AAGGAAAAAAATAAAAATAAAAATTGATTCCTTAAAATTAAATGAATAAAAAATTC
AGTGAATTCTGGGGTGGGTTTGATTGGGCAAATTCTTGCTTATATTTCCATCATATCA
ATGTGTAACAAGTCACGATAAATAAAATAAGATAAGAAGACACATGTTGCATTAGC
AAGTTGATTGTTGAATGTTTGATTGGGGCGATTAATAAGAATAAACATGTAGCATAT
GTGCCTATTTGTACCGACAATTTTATATAAAATTTATTGTATTGAGATGTTTACATAA
TTTATTTATTTATTTTTTTTGCAGTTTGAGTGCGTTTAAATACAAAATTTTGAAGACA
AATGATTTTAAGTTATATTGAAAACTAATTGGTGACTAGTACGTAGGTATTAACCAA
TTTAATTTTATATTTTGTAATTTCAAGGGTCATCATTGACTTCTACTCCCATTAGATC
AATATTGTTGATATATGTATGACAAAGGTTAATGTGATGGATCCATCATCGGTCTCT
AGAACCGTTGGATTCATCGGTCCTTAAATCTATTGGATTCAAAAATGATATGGAAAT
TCCGATTTTGAATCTTGGTATGAGGTAGGAAAAGAAATGATATATATATTTTTTTTCA
AAAAAAAAAAAAAAAAAAACTCTTATTTGACATGCCTTAGTGCATATCTCATTTCTTA
ATGCCAGTTAGAGAATATTAAAACGAAATTTTGAGTGTAATTATTATTCTTTTAGGT
AATTAGGTGGACTTATTTAGCTGACTTGATCAACATGTTTTTTTATTATTTTTAAATT
ATTTTTTATTTTGATTGCAACATGTCATATTAATTTATTCTAAAATGACGATAGTACT
AGGGCAAGATAATTTTGTCATTTCCGAATTTCATAAAGTCAAAAGGAAAAAATTTCAA
TGACTTTAAGCAATTGTAATATATTAAGTTAATTATTATTTTTAAATGAGAGATAGAT
TAACTAACATGATATATTAATTATATTGTAAGTGTAAATCAATTATATATACATATTT
TTGTTTTATTGACTCTTTACGAAGATAGACCTCAAACCCTTTTAGGACACACGTCTAG
GATTAGTTCAACTCTCGAGGGAAACAAAATTGTATTAAGAAAACTGTTACCTTATCA
ACAATGCTTCGTCCGTAAGATTTAAAAACCTAAGAGTCCAATGCAGCTTATCCTACT
AAGCTAGTAGGTTATTTGAACATACCACAAGCAAATAAAAGTATGAAATAAAACGG
TAAAACAAATCATAACATTTTTTTCACTTTTAATGAAGTTTTATTTACCAAAGAAAA
ATTATACACTACCACCGAGTGCTTCGTCCAAGTGGTAGGGAAGATGCTCTTTCTCTC
CTGACGCTTGTGTTCGAACCTTGGCTTACGCATAGGCTAATATTGGGGGTAGAAATT
CTTCTTAAATGGGTTAGGCTCAATACGCTCGTGGTAGACCTCTAACTTTCACCCATTC
AGAGGACCGACTTAGAAAGTAGCGCGATTCGTGCCCGACAAAGCCGGTTGAGGGAC
CGAACGTGAACTTGGGTGGGGGCAAAGCCTCTCCCCCCAAAAAAAAAAAAAAAAATA
TACACAAAAATAAACACATAAGTGTAGGAAGACTAATATCAAGTATTTCTTATATAT
ATATATGCTAGAGAAATTTTATTAAGAAAAAAGAAAAAATTACATGAAAATAAACG

AAAAAATTTATAAGAAATTTTTGACTATATACATATATATTGTGTTGAATTATATAA
GAAATTTTAATTTCGGGTCAAATACTGTATTATTTGTTAAAAAAGAAAAAGGAAAAT
GGGATTTACCAAAGAAGGAAAAATGTAAAAAAGAACCGCGCAGACTGATTTTCAAT
TTTCATTTTTAATAATTTCAGTAGATACTGACATCATTTATTTATCCAGCTCCAATCT
GATAAAACTGAGTTAAAAGGGGGAAAAATACTGTGTCAAAACCAGCCCACAGGAA
AAAAAAAAAAAATTAAAAAAAGGAAAGAGAAAATAAAGGAAAGGAAGAAGGAACA
GGGAAGGGAAGATTTTTCAGCAGCAGACCCAATCAAGAACTCGTTTTTGGTTGAATA
GAAAAAGAAAAAAGATTCTTGGCCATGACAGACTCCATTTTTCTTGCCTTAAAAGCT
TGACTCAGTCCATCTCCTCTTCTCTCTTCTCGTCTTTTCTCTTTTCTTGAATCCTTTCTA
GCTCAAACCCAAATCAAACAACA

>NISI08G1156

AATATTCTATAGATTTCGTAATGAAACAAAGTAACATAACAGAATGAACAAAAACA
CCGTTCGAAAACGACGTCGTTGGCGTTCAAACTGAGGAATGACTACTACTAGATTCA
TCTCAGACAACCTTTGACCCAATGTCTCTTAGCAACGCGTGCTTAACACGTGCCATT
CCGCCACGTGCAAATGTTCCTTCTTTGGATACCCGCTATCCACTCTCTTCCTCAACAA
AGGTCAATGGTGACGCCGAATTGCTTATTGGGTAACTTTTTATATTTTCAGAATGTTC
TACAAACTTATATATTTATCATTTTTACCTTCTCTTTTGAAAGTCATTCAGAAACCCC
CTTGTTAATAACTTATTTGGATAATGGTCATTTCGCTAATGGTTTATTAAGTTACATG
CACTATATAATACTAAAGTTGGAATCTTAATATTAAAATTTTTAGTATAGCATTATTT
TTGTACTATTTACAAGGTTGTCTTTGTTAATTTTCACACATTTCGATCAATATCCACTT
GCAGGTAGGTAGGGATGGTAATATGGATCCAGGATTTGTTTATCCAAACTAGATCCG
ACACAAAACGGATATTTTTAACCCACATAAAATGAACCCTTAAGTGGGAAACGAAG
CCCACATCCGAATTAAAAAATGAGTCGGTGGCAAATCTAAGATTCAATATCCGTTTA
CCCATTTTTATCCTGCTACAAGAGGTTAAGTGAAATAAATTTACATTTATTAAACAT
AGAATGAGAGAAATGAAAGAAATTTAAAGTTTTCATAAAAAATTTAGTTTTTTTCTTT
TTTGAAAATTATAAAATTTAGCTTAACATAACATTATTTTTTAAAATTACTTGATCAA
TACTATGATTTATTTACATTACTTATCATGTCGTATTATTTTAATTTTTCATATGTTTT
TCAATTTACTGTTGCCATTATCGTACTTGGTTCTATTTTTACTTTTATGAAATAATTAG
AAATTGTTAATATACCGCCATTGTACTATACCGCAAATGAGCCTTTTTCTTTTATTGG
CATCTGTCCCGAAAGCCACAAAAAAATGTAAAAGTGAGTGGCTATTTGGCAATGCC
AAAAAATACGAACAAAAAGATGGTGGCTAGGCCACACTCCACAAGAAGAAACTTTG
TGGTGTGATACCATAGCCACATTAAAGAGTGCAGACGGGTGTGTCTGAACCACAGC
CTTTGAAGGAGAAAAAATTGTTGTGATATCATAAAGTCACACCCCACATATATTATT
TAGGTGAGGTTGTGGTTGAACAACATGACCACATGAAAATATAGTGACTGTTGTTGT
GTGACAACATTTATATAAGAAAAAAGTAGTTTATTTATGACTTTGCCACACAGTCTC
AAAATAAGATAGGGATAGTAAGTGGCTTAGCCACATTCACTGTATTTGGATATGTGG
CTGTGTGGCTTAGCCACAGCTTTAAATTGAAGAGTTGTGTTGTTGTCCGATTGAATG
ACGAAGTGTAACCCTTACATTTTTAAGGATTGTAAATTTTAAATCTAACAGTCGAAA
CACCTTCGTCCTCAACCATGAGATCATTTCTCAATCCTAGGGCTGAGATTTTTCGGTA
TTATACATAAACGGTACATTAGCAATCTCCGAAATAATTTAATGGTTCAATTTCTCA
AACTTGAAAAGTGTAGACCAAATCAACATTTTTGTCAACCGAAAAATATGTATTTTG
ATTTCCAAATTAAATGAAAAATAAAGTCGCATCAGATTTTGATATTTTGTTATTGGTC

CACAGAGTCTACATTGACTTTTAAATATAACAAACCAAAGAAGGTGTTCTTTACAGT
TTCTAAAAGATACTTGCTAAAACGATAATTTAATCAAAACTGAAGGGCTTCCACGCA
ATTATCCACTTCGTAATGACAGTAAGAGAGAGAAAGAGAGAGACTTTTTGGGATCTT
TACTTGGATCATCTCATCAGTGACTTCCTCGAAGCCGTATAAATAATAATAATAATA
ATTAATTAATTATTATTCCTCTTTTTTTTTTTTCCTAATAAAAGTAAACAGAAAAAGA
ATTTAAAAAAGGAAACTTTTCCTTTTTCTATCAAAAATCATTTCCTTATTGATTGTTT
GATTCAGTGCATATGGCAATGATGATGACAACGACGATGACGACGAAGAAGGTGCT
GTTAATGGTGGTGGTCAGATGGTAAAAGCCGCGTTTTAAAGGAAAATCGAAAGAAG
GAATTTTGAGTTTTGAGCTGGTCAGCACTCTCTCTCCCTCCGATGTTTCTTGGCAGCT
GCTTGCCAAAAATAACTTCAATTCAATCCAATCATTCATCCTAATTTAATTCATTATT
AATGTTCGCTCGTTCAATTAATTATTTATTTAATTAAAGGCTGCTTGTTTTGGATTAT
TCTTCGTTCGAGATCGCTCCCATCCATTTCCATCTCCCTTTTTATTCTGATTTCCGCGT
CATTTCCGGTCCATTTCCATCCCCTTTTTCCCTCTTCTTTGAATAACTCCGATTGGTAA
TCATAGATCCGTCTGCTTCTCTTTTTCTGTTTCCTTTTTCCATTCGTCCGTGTTGTAAT
TGTATGTACGATTGTCTGTTTATTATTTGTTCTTGCATTTGCGTTTAATTTGGTTTTCT
AATTCGTGATCGTGTCATCGTGATGTATCGGTTTAAAATTAGGGTTTATTTTCGTTCG
ATAAATCCGTTTATTCCGTGTAAATTGTGCATGGCGATTAATAATTAGGGTTAATGA
TGATCTCTTTTCTTTGCATTAAGATTGTGAAAGGTATCGCTTTGTCTGAAAAAGGAA
AACCTAATATTGAAGTCTGATCTGATATGGATTGCTTTGATTCTCGGATTTGTAATTT
CTCAGTTTTTCTAATTTAGGGATTCTTGACTGTGAGTTGCTTTTTCAGTTTAATTTATG
AGCTATTGATTATTGGATGAGGAAAATATCGTTCAGTGAGGAGATATTGAGGAAAC
TCTAATTGTTTC

>NISI09G0307

AAAATTATATATAATTTCTTTTTTATTTTTTATTTTATTCAATAGTAAAATAATTATCA
TTTATATTATTTTTGCCACATTAACAAAACTTAATAGAAAATACTCATAGTAAGGGC
ATGGATGACATTACTGAATCTTCCAGTTATATTTGATATAATAGTTTGTAGGGGCAT
GAATAAAATTATCCCTTGAAATATTTATTTCATTAAAACCCTTCATCGTCAAGCTCAA
TTAGACATTCAGGGATCCAAGCCATATTTACACTTTGAGGTAGAAATTCTAAATCTA
ACCTCATATTAGACTAGCCAGGGAGAATAGGTGCTTGGTCACCATGGTTTCTCACTT
CCCCTGCCACCTTGATGGCCAGGGTTCAATTCCTACTCTCCGCAATTGTGAAACGTG
GGGATGTGGGCAGATGTCTGCTCTTGTAGGAATAGATTTGTCCCGATTTATGGGATT
GGTTATCTGAGCCGCGCTAAATAAGAAAATATCTAAGTGTTTGGTCCCAACAGTTGT
AATCATTCTTCAAAAAAAAAAAAGATTAGCCAGGTCAGTCCGTCCATAATCTGTGCAT
TGTGCACACTAATAGTTTATTATTTCATTCAAACTGGATGGTTGATTTTTATTTTTTTT
TAAATTTTTTGAAAAAAAAAAAACCTCCAAAATTCTCTCATATTCTCTTTCAGATATTC
GAATATGGTTTTCCTTAGAAGAATAAATGAAAAGTATACCACCATAATAAACTCATG
AAAGTTCCCAAGTGAGTGGATATGATCCTATATCTCATCGATTTGGATTCACCTAGA
TTCATTTTTCAATGGATTAAATATTATATATATTTTAAAATCTAATAATAGATTGGAT
AGGATATATATTTAACCAAATCTAGGACGATTCAATTCATGAGTACATCTATTAATG
AATTTCATAAATGATTAAATGTTAAGATCATTTATTCAGATTATCGATTGATTTCCAT
TCTTTAAGATGCAATTTCAAATTTTAATTTATTAAAAAAAAAAAAGGTAAAGTTAAGA
TTTTATATTGTTTTTTTTTTTTTTTTTTTTTTTTAAAAAAAAAAAACTGATATCAATGGA

TCTAAGATATGTTAAGATTTATCGAGTTAGATTGAGATATGAGTTTTATGTATCACA
ACATATGGACAGATGCTTACAAGAAAAGATGTGCACTATGACACTTGAATAAAAAT
GGATACTTCTAATTTGTATGAAAAATATTGTGTATCTCAAATAAAAAACATAAAGCG
AGATCCCAAATTATATGACTGTTTATGAGTCGATTATGTAAAATGTAATTAAAATAA
AAATGAAAATGATTTTTCAATCACAAATAATCATATTATTTGAGATTTCAATTTACG
ATTTTAATATAAGATAAATAAAAATTTCCCATTAATATAAGAGAAATGATGTTTACT
GAATCATTATAGTCACTTTTTATAACTAACTTTTGATATATCAAGATACGATTAGTTG
AATAATTATATATTTATTTATAAATAATCAATCAATTTTTATAAGATACTCGGATAGT
CAATATATGTTTTTTTTTTTTTAATGAATTTTCTATAGTGCGATTTAGATTTTTTTTCAT
AATCGAAAATTAAAGCCAAAGGGAAAGAGGATATATTATTTTTTCTTAAAAAAAAG
AAAAAGAAAAAGAAAAAAAAGGATATTTTAGTATATTCACTACAAACATTTGAAAA
GTTTTAAACTCACTTAATTAAGACACCCTCCACGTGGAAGATTTCTATTGGATGGGA
ACAAAATTTGAGAACTCTGAACCATACAGTTAAAAAAGTATGAAGATTGATAACAT
GGCAAAATCCAATAATTAAATGCATTAAAATATTTCCCTTGTAATAATGTCAGAAAA
ATACTCAAAATGAACATTACGTTACACTGAACACTACCAAAAATAATTATTATTGTT
ATATTCTCCACATTATTATTATTTTATTATTTAAAAAAAGGATAAAAAAGGGGAAAA
AATAAGATAAATTAAATAAACACGACCTTAACAATTGTTTTCTTTCAGTTTCAGATG
GCAGGCTTTTGTGTTTCTCATTACACCTGAATTTTTCTTATTAAATAAATAGATTAAG
GATTAGAATATTAGAAAAAAATTATTAAAATTAAACCGAAAATTATTTTTAAAAAA
AAAAGGAAAAAAGAGAAGAGAAAAAAGGGGGAAGGCAGAAGGGCTGTGTCGTTCT
TCTGTCTTACTTACATTTACAATTACAAGGACGTTGAATCTTAAGAGGAGAAAGAGA
AAAAGAAAAGCAAAAAAGAGAGAGAGAGAAGGAGAAAAATCCTTTTGCGAAAGAA
GCAAGCAAGCAAGCTTCCTTTGACTCTCTATCTCCCTCCCTCTCTCCTGGTCATTACC
AAATTTCCTTTCTTTTTCTATCTTAACATTAAATTCATCGTTCCATTTTTCATTTCTATT
TTCTTTTTAATTTTTTCTTTCTGTTCCGGACAGAAAAACCTTTTCTTTTCCCAAAACCC
CATCTGGGTCTTCTTCAAGATTTACTCTTTATCGTTATCAGCAATCCAGTCGTACAAA
TCCGAGGTGGGTTTTCTTTTTTCTTTTTTTCCTTGGCATTTCGTTTTTGAGCTCATTCTT
TTATCTGGGTTTTGTGGATTAATGGAAACTTGAGAAAATATTTGATAAATGTTCTTGT
TTGTATGGTTCCCGTCACACAAATTTTATTATTAGTTATAAGGGTATGTTGCTCTGGG
TTTCTGGATAATTGATTGTTTATGCTGCTTGATCTCGAGATATTTTAAACCTTCCAAA
TTTTATTGGAAAATCTTTTAGTAGGGTTAGATTGGGAAATTAGGGTTATGCAGCTGA
GTTTTTTATTTTCATTTCTGGGACTTACTTATTATGTATTGTTGGAAAAGAAACTGAA
ATTCTGATTTTTGATATCATGAACTAGGTCAAATTTGGAGGTTCCGTGATTTTGAGAA
TTTAAGCATTAATTC

>NISI09G0177

TAAAATAGTTCAGTCCAGTTCTGTATACCAAAAACAGCTTAAAAGTATTTGCGGTGA
TTTTTAAGAAAATTGATAGTCTCACTCAGCCTTTTATGCATGTTTACTAAAACCTTCA
CTTAATCTTGTATATTTCATAAACCTTAACTTAAGCACTTCATTGTTGTGGTGGATAT
ATTAGTCAGCTTGCATTAGTGAGTTAGTGCTCACGTGTGATGTACATGCAAGTTTATT
GTTAAGCTGATAATTAGTTGGTTAATGAGGGTAGTTAGTATAGTTAATATCAGCTGT
TATGTTAGCTATATAAACCCTGTTGTATCTTCATTTATAGTTGAGTGAGATTACACAA
TCGACATCTCCAATAGATCCTTCTCTCTTTCCATTGTTACTTCTTCACTTGATTTCATA

ATTTGTTATTCATGTTTAATAAAACTTTCATATAGCCTCGATTTATTTACTAAACCTT
CACATAGTCTTCTATGTTTATTAAAAGCCTTCATTTAGCTACTGTTTCGTCAACATTT
GTTAAGTTGACCGATAAAAAATGAGATTGCATAATTACCATTGATGACCTTATGACA
GATTTATTAACTTAAATATCTTGTAAAATTACAACAAGAAAAAAAAAAAAAAAAATT
AAAGGGAAAAATTATGGGTGTTTATAGTTCCTCAAAATGAGACTATTTTTGTTATTT
CACGAGATATTTAAAGCTAACAAATTTGTCTTGAGGTAGACAACAGTATTTATGTAA
TATCACATGATATGTTGAATATTTTTAACAAAAGGGCTAGATGAAATTTCAAATTAA
AGTTTATGAAACATACGAGACAAAGTAAATGTTTTGGTAATCATAGGTGGATTAGGT
GAAATTATCTCTTAAGGAAAATGAAGGTATTTGCTTTTTATTAGGAATTACATGTAT
CATATATCTTATAATATACATTGTATAATTACTGGGATCTATTATCTAGATATTTTTT
ATCTTATAGTGTGATTTAGATGTCTTGTCACATTTTTAGATAATTTTGGTCTCATTTTA
CGACATTTCTATGTCCATCGTAGGGAAACTAGTCTATTGTTCACATTCCCCCTCTTCC
CCCCCCCCCTCCCCCCCCCCTCTTTCTTTTTTTTTTTTAAACCTAAAATCACTATAAGA
GAGGTAGGAATGCCTTTTCTGCTAGACTACAAGGTCATTGTCAAAGAAAATGAAGG
TATTTGAAGACACCTTAACAGTAAGGCACATAACGACTTAACTAAAACGATGAATT
GAATGTTATCCCATGGTAGTTTTGGGTATGTAGTTCTAAGCATAGCTTGTCAATAAT
GCATGGTTTCTCATTAACAGACTTGGCATTTAGACTCCTTATGAAGTATAGGCTCGA
GCTCGACTGAAGATGCAATATTCTAGCAGCTGTATGAGTATGAGTAATAGCTGTAAA
AAGTAGAATATTAGTTTCAACAACTTGAACAAAAACTGCAATCCACAGTCAACACA
GAACGAAGGTTGGAGTGCCAAAAATCTATATGCATGGTTTTTTTTTTTGGTAGTAGT
AGTAGCTATCACTGATAGATAGACATCGCACTCAATATATAAGTCTTTACCAGACTC
TTATGAGGCATAATGTATCAGGCTACTCGATGAACACATGTTTTTTTGGATTATCATA
AGGTGTTCGGACCAGTTTTCGTGCACCTCGATTAATCAAGGTTTGAGGCGAGAACAC
TAACGATAAATCCCTTCTAATAATGGAGACTCTAAGTTTTAAATTCAAGACCTTTTG
GTATAGAGAATCATTTAATTTGATTGTAGCTACATCAATTCATCGTACGAGGAATTT
AAATTGTTTCGAATATATATAATGTTAATATTCGAACCAGTAAGTGCCTTCAGTTCA
AGTCTCAATCACCAAAACTACAATAGTCCTCCAAAGAAATAGATTTGAATTTTTCTG
TTGGAAAAATATATGTTATTCTTTGGTATTTTATCTACAGGGTGTCTGCACTAGTTTT
CTCACACCTTAATTAATATATATATCATGTTAATATTCGAATCAGTAAGTGCCTTCAG
TTCAAGTCTCAACCACCAAAACTGCAATAGTCCTCCAAAGAAATAGATATGAATTTT
TTTGTTGGAAAAATACATGTTATTCTTTGGTATTTTATCGATAGAGTGTCCGCACTAG
TTTTTGCGCACATTGATTAATCTCCACTTGCGATGCATGGATGCCTTCAAAACGAAA
AATTAATTCTATTTTACTGCTAACTTTGCAACATACCCAAAAAAAAGAAGGAAAAAA
AAAAAAAAAAAAAAAAAGGAAGGAAACAAGAGAGGTTAAATTGTTTGTGTAAGCT
CGGAAAGTCCATAGTTGGGTATAGTTAGCAACAGGCCAGCTTAGCATAGTAGCTGT
GTAGTCTAATGATAGGCTTCAAATGTTTTGCAAAACAAGTTATTTATATTCCAAATT
AAAAATGAGAAGAAAAAACAGTTAAGAAAAGAGGCGGAAAATGCTCTCAACCTTT
GTATATGAAAAAAAATGAGAAAATTTCAACAGAAAGGGACACGCATTGTCCTAAAA
TCCCTCACTATCAATCCTCTCCACTTTCTGCACCAAAAATCCAAGCTGTTGTTCTAAC
CCCCTCCCCTTCTGTCTCTTCTCTTAATTTTATTACTCTTTCTATACATGTCTTTCTTTG
TCTGAAGTACTCTTTCTTCAGCCCCTCATCAAGACAGAAAAAAGGAAAAAAAGAAG
AAGAAAAAAAAGAAAAAAAAAGAAAACTTTTGACAAAAAAAATAAAATAATCTTT
TTTTTTTTTGGGGAAAAATTAGGATAAAAGAAATTCAATAGGCCTTTTATCTCATTA

GGGCCATCACCCCTCCCCCTCCCCAACAAAGAAAATCTTTCTCTTTTTTTTTTTTTTC
TCAATTTCTGTCTTTTTCTCTATATTTTTTTTGAATATCTCCCTTGGAAAAAAAAAAA
AAAAAAGTAAAAGAAAAAGAGAA

>NISI09G0294

CCTAAAATTATATATAATTTCTTTTTTATTTTTTATTTTATTCAATAGTAAAATAATTA
TCATTTATATTATTTTTGCCACGTTAACAAAACTTAATAGAAAATACTCATAGTAAG
GGCATGGATGACATTACTGAATCTTCCAGTTATATTTGATATAATAGTTTGTAGGGG
CATGAATAAAATTATCCCTTGAAATATTTATTTCATTAAAACCCTTCATCGTCAAGCT
CAATTAGACATTCAGGGATCCAAGCCATATTTACACTTTGAGGTAGAAATTCTAAAT
CTAACCTCATATTAGACTAGCCAGGGAGAATAGGTGCTTGGTCACCATGGTTTCTCA
CTTCCCCTGCCACCTTGATGGCCAGGGTTCAATTCCTACTCTTCGCAATTGTGAAACG
TGGGGATGTGGGCAGATGTCTGCTCTTGTAGGAATAGATTTGTCCCGATTTATGGGA
TTGGTTATCTGAGCCGCGCTAAATAAGAAAATATCTAAGTGTTTGGTCCCAACAGTT
GTAATCATTCTTAAAAAAAAAAAAAAGATTAGCCAGGTCAGTCCGTCCATAATCTGTGC
ATTGTGCACACTAATAGTTTATTATTTCATTCAAACTGGATGGTTGATTTTTATTTTTT
TTTAAATTTTTTGAAAAAAAAAAAAACCTCCAAAATTCTCTCATATTCTCTTTCAGATA
TTCGAATATGGTTTTCCTTAGAAGAATAAATGAAAAGTATACCACCATAATAAACCC
ATGAAAGTTCCCAAGTGAGTGGATATGATCCTATATCTCATCGATTTGGATTCACCT
AGATTCATTTTTCAATGGATTAAATATTATATATATTTTAAAATATAATAATAGATTG
GATAGGATATATATTTAACCAAATCTAGGACGATTCAATTCATGAGTCCATCTATTA
ATGAATTTCATAAATGATTAAATGTTAAGATCATTTATTCAGATTATCGATTGATTTC
CATTCTTTAAGATGCAATTTCAAATTTTAATTTATTAAAAAAAAAAAAAAAGGTAAAG
TTAAGATTTTATATTGTTTTTTTTTTTAAAAAAAAAAAAAAACTGATATCAATGGATC
TAAGATATGTTAAGATTTATCGAGTTAGATTGAAATATGAGTTTTATGTATCACAAC
ATATGGACAGATGCTTACAAGAAAAGATGTGCACTATGACACTTGAATAAAAATGG
ATACTTCTAATTTGTATGAAAAATATTGTGTATCTCAAATAAAAAACATAAAGCGAG
ATCCCAAATTATATGACTGTTTATGAGTCGATTATGTAAAATATAATTAAAATAAAA
ATGAAAATGATTGTTCAATCACAAATAATCATATTATTTGAGATTTCAATTTACGATT
TTAATATAAGATAAATAAAAATTTCCCATTAATATAAGAGAAATGATGTTTACTGAA
TCATTATAGTCACTTTTTATAACTAACTTTTGATATATCAAGATACGATTAGTTGAAT
AATTATATATTTATTTATAAATAATCAATCAATTTTTATAAGATACTCGGATAGTCAA
TATATGTTTTTTTTTTTTTTTTTAATGAATTTTCTATAGTGCGATTTATATTTTTTTTCA
TAATCGAAAATTAAAGCCAAAGGGAAGAGGATATATTATTTTTTCTTAAAAAAAGA
AAAAGAAAAAGAAAAAAAGGATATTTTAGTATATTCACTACAAACATTTGAAAAGT
TTTAAACTCACTTAATTAAGACACCCTCCACGTGGAAGATTTCTATTGGATGGGAAC
AAATTTGAGAACTCTGAACCATACAGTTAAAAAAGTATGAAGATTGATAACATGGC
AAAATCCAATAATTAAATGCATTAAAATATTTCCCTTGTAATATTGTCAGAAAAATA
CTCAAAATGAACATTACGTTACACTGAACACTACCAAAAAAATAATTATTATTGTTA
TATTCTCCACATTATTACTATTTTATTATTTAAAAAAAAGGATAAAAAAGGGGAAAA
AATAAGATAAATTAAATAAACACGACCTTAACAATTGTTTTCTTTCAGTTTCAGATG
GCAGGCTTTTGTGTTTCTCATTACACCTGAATTTTTCTTATTAAATAAATAGATTAAG
GATTAGAAAATTAGAAAAAAATTATTAAAATTAAACCGAAAATTATTTTTAAAAAA

AAGGAAAAAAGAGAAGAGAAAAAAGGGGGAAGGCAGAAGGGCTGTGTCGTTCTTC
TGTCTTACTTACATTTACAATTACAAGGACGTTGAATCTTAAGAGGAGAAAGAGAAA
AAGAAAAGCAAAAAAGAGAGAGAGAGAAGGAGAAAAATCCTTTTGCGAAAGAAGC
AAGCAAGCAAGCTTCCTTTGACTCTCTATCTCCCTCCCTCTCTCCTGGTCATTACCAA
ATTTCCTTTCTTTTTCTATCTTAACATTAAATTCATCGTTCCATTTTTCATTTCTATTTT
CTTTTTAATTTTTTCTTTCTGTTCCGGACAGAAAAACCTTTTCTTTTCCCAAAACCCCA
TCTGGGTCTTCTTCAAGATTTACTCTTTATCGTTATCAGCAATCCAGTCGTACAAATC
CGAGGTGGGTTTTCTTTTTTCTTTTTTTCCTTGGCATTTCGTTTTTGAGCTCATTCTTTT
ATCTGGGTTTTGTGGATTAATGGAAACTTGAGAAAATATTTGATAAATGTTCTTGTTT
GTATGGTTCCCGTCACACAAATTTTATTATTAGTTATAAGGGTATGTTGCTCTGGGTT
TCTGGATAATTGATTGTTTATGCTGCTTGATCTCGAGATATTTTAAACCTTCCAAATT
TTATTGGAAAATCTTTTAGTAGGGTTAGATTGGGAAATTAGGGTTATGCAGCTGAGT
TTTTTATTTTCATTTCTGGGACTTACTTATTATGTATTGTTGGAAAAGAAACTGAAAT
TCTGATTTTTGATATCATGAACTAGGTCAAATTTGGAGGTTCCGTGATTTTGAGAATT
TAAGCATTAATTC
