# Supplementary figures and images for: Classification of Calcium-Dependent Protein Kinases and Their Transcriptional Response to Abiotic Stresses in Halophyte Nitraria sibirica
Source: Plants (Basel). 2025 Oct 7;14(19):3091. doi: 10.3390/plants14193091 (PMC12526309; doi:10.3390/plants14193091)

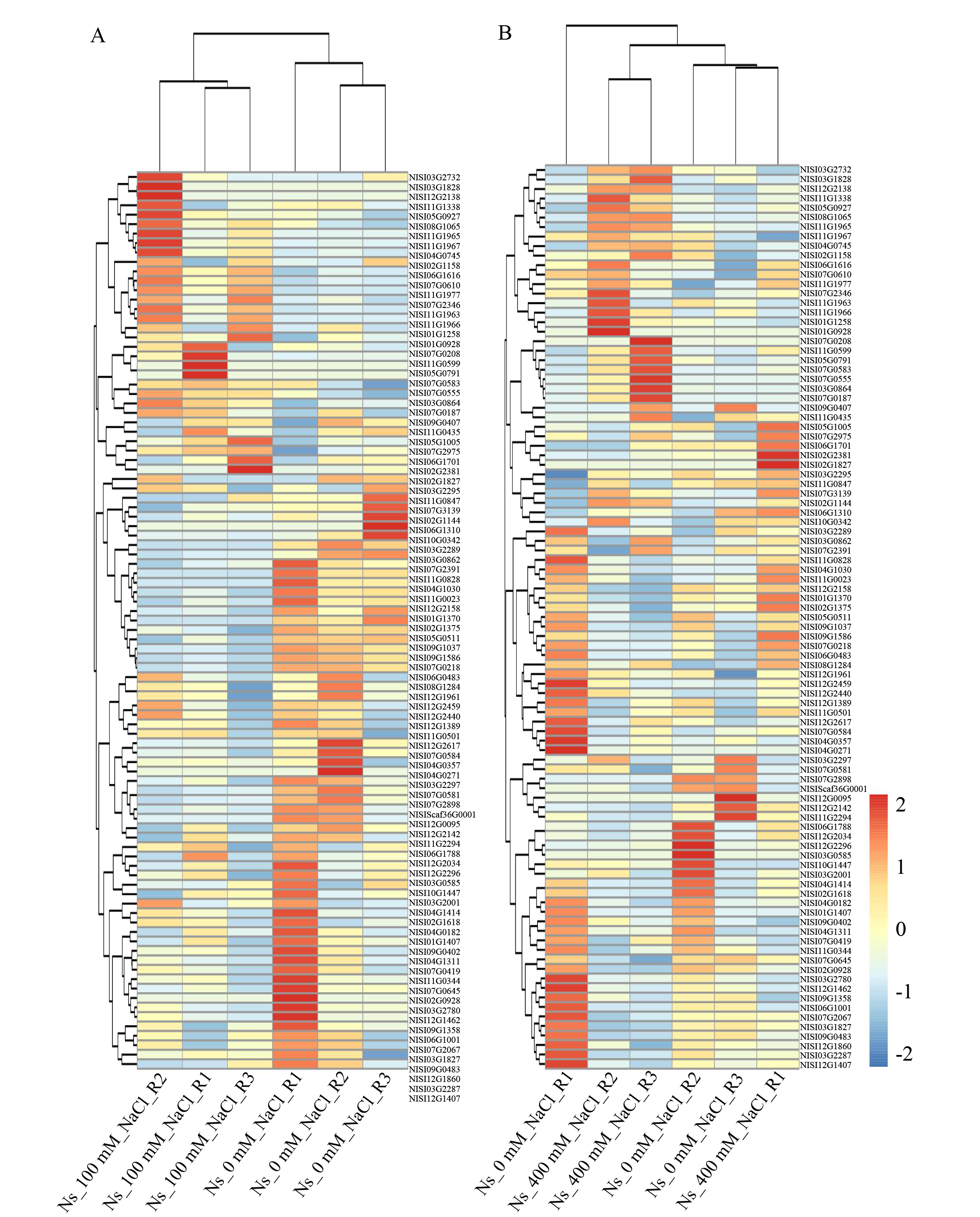

Supplement: Supplementary file 1 [file plants-14-03091-s001.zip › Supplementary Figure S1.tif]

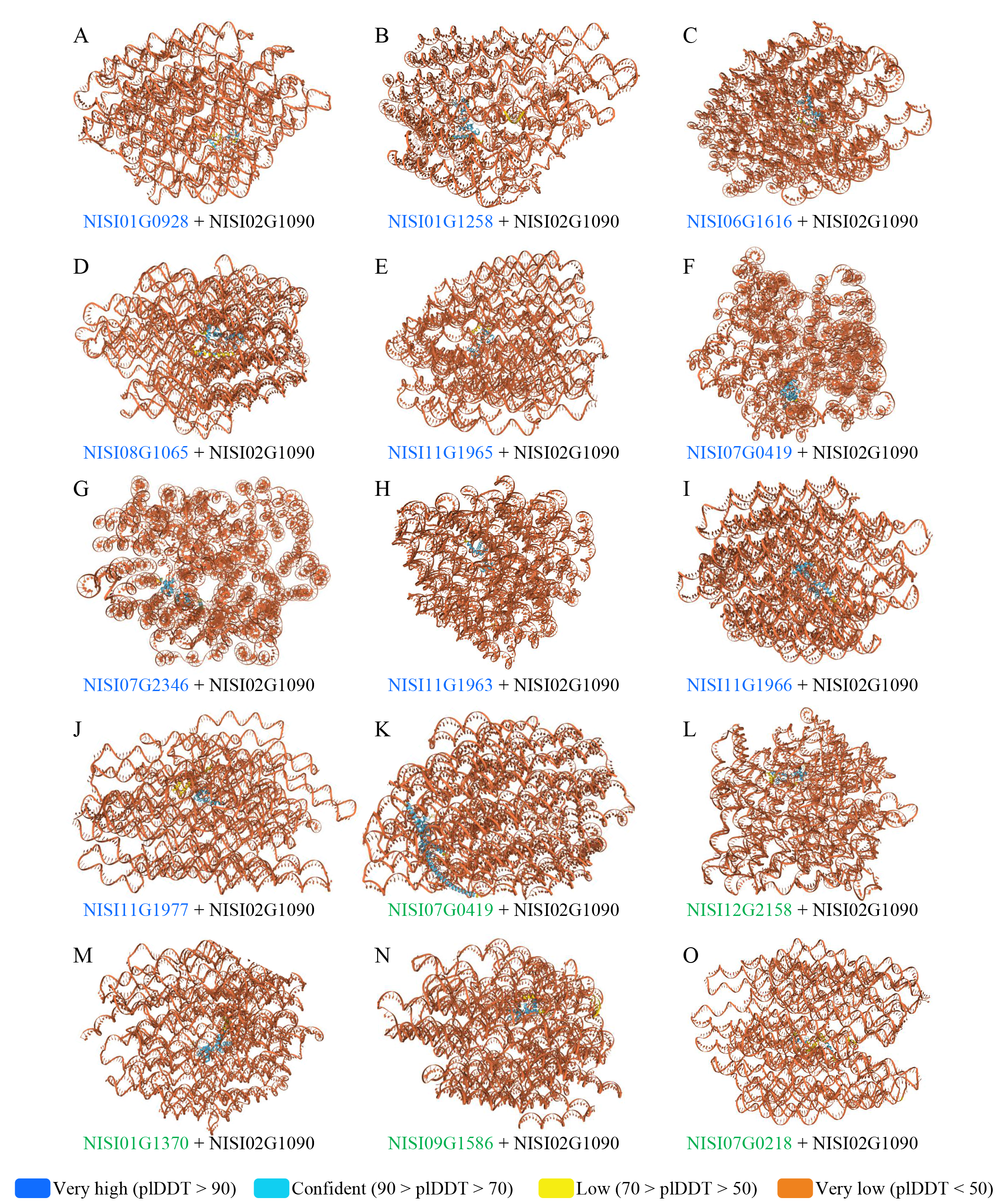

Supplement: Supplementary file 1 [file plants-14-03091-s001.zip › Supplementary Figure S2.tif]
